# Annual (2023) taxonomic update of RNA-directed RNA polymerase-encoding negative-sense RNA viruses (realm *Riboviria*: kingdom *Orthornavirae*: phylum *Negarnaviricota*)

**DOI:** 10.1099/jgv.0.001864

**Published:** 2023-08-25

**Authors:** Jens H. Kuhn, Junya Abe, Scott Adkins, Sergey V. Alkhovsky, Tatjana Avšič-Županc, María A. Ayllón, Justin Bahl, Anne Balkema-Buschmann, Matthew J. Ballinger, Virendra Kumar Baranwal, Martin Beer, Nicolas Bejerman, Éric Bergeron, Nadine Biedenkopf, Carol D. Blair, Kim R. Blasdell, Arnaud G. Blouin, Steven B. Bradfute, Thomas Briese, Paul A. Brown, Ursula J. Buchholz, Michael J. Buchmeier, Alexander Bukreyev, Felicity Burt, Carmen Büttner, Charles H. Calisher, Mengji Cao, Inmaculada Casas, Kartik Chandran, Rémi N. Charrel, Krishna Kumar Chaturvedi, Kar Mun Chooi, Anya Crane, Elena Dal Bó, Juan Carlos de la Torre, William M. de Souza, Rik L. de Swart, Humberto Debat, Nolwenn M. Dheilly, Nicholas Di Paola, Francesco Di Serio, Ralf G. Dietzgen, Michele Digiaro, J. Felix Drexler, W. Paul Duprex, Ralf Dürrwald, Andrew J. Easton, Toufic Elbeaino, Koray Ergünay, Guozhong Feng, Andrew E. Firth, Anthony R. Fooks, Pierre B. H. Formenty, Juliana Freitas-Astúa, Selma Gago-Zachert, María Laura García, Adolfo García-Sastre, Aura R. Garrison, Thomas R. Gaskin, Wenjie Gong, Jean-Paul J. Gonzalez, JoëlleGoüy de Bellocq, Anthony Griffiths, Martin H. Groschup, Ines Günther, Stephan Günther, John Hammond, Yusuke Hasegawa, Kazusa Hayashi, Jussi Hepojoki, Colleen M. Higgins, Seiji Hongō, Masayuki Horie, Holly R. Hughes, Adam J. Hume, Timothy H. Hyndman, Kenichi Ikeda, Dàohóng Jiāng, Gilda B. Jonson, Sandra Junglen, Boris Klempa, Jonas Klingström, Hideki Kondō, Eugene V. Koonin, Mart Krupovic, Kenji Kubota, Gael Kurath, Lies Laenen, Amy J. Lambert, Jiànróng Lǐ, Jun-Min Li, Ran Liu, Igor S. Lukashevich, Robin M. MacDiarmid, Piet Maes, Marco Marklewitz, Sergio H. Marshall, Shin-Yi L. Marzano, John W. McCauley, Ali Mirazimi, Elke Mühlberger, Tomoyuki Nabeshima, Rayapati Naidu, Tomohide Natsuaki, Beatriz Navarro, José A. Navarro, Yutaro Neriya, Sergey V. Netesov, Gabriele Neumann, Norbert Nowotny, Márcio R. T. Nunes, Francisco M. Ochoa-Corona, Tomoyuki Okada, Gustavo Palacios, Vicente Pallás, Anna Papa, Sofia Paraskevopoulou, Colin R. Parrish, Alex Pauvolid-Corrêa, Janusz T. Pawęska, Daniel R. Pérez, Florian Pfaff, Richard K. Plemper, Thomas S. Postler, Lee O. Rabbidge, Sheli R. Radoshitzky, Pedro L. Ramos-González, Marius Rehanek, Renato O. Resende, Carina A. Reyes, Thaís C. S. Rodrigues, Víctor Romanowski, Dennis Rubbenstroth, Luisa Rubino, Jonathan A. Runstadler, Sead Sabanadzovic, Sabrina Sadiq, Maria S. Salvato, Takahide Sasaya, Martin Schwemmle, Stephen R. Sharpe, Mang Shi, Yoshifumi Shimomoto, Venkidusamy Kavi Sidharthan, Manuela Sironi, Sophie Smither, Jin-Won Song, Kirsten M. Spann, Jessica R. Spengler, Mark D. Stenglein, Ayato Takada, Sawana Takeyama, Akio Tatara, Robert B. Tesh, Natalie J. Thornburg, Xin Tian, Nicole D. Tischler, Yasuhiro Tomitaka, Keizō Tomonaga, Noël Tordo, Changchun Tu, Massimo Turina, Ioannis E. Tzanetakis, Anna Maria Vaira, Bernadette van den Hoogen, Bert Vanmechelen, Nikos Vasilakis, Martin Verbeek, Susanne von Bargen, Jiro Wada, Victoria Wahl, Peter J. Walker, Thomas B. Waltzek, Anna E. Whitfield, Yuri I. Wolf, Han Xia, Evanthia Xylogianni, Hironobu Yanagisawa, Kazutaka Yano, Gongyin Ye, Zhiming Yuan, F. Murilo Zerbini, Guilin Zhang, Song Zhang, Yong-Zhen Zhang, Lu Zhao, Arnfinn Lodden Økland

**Affiliations:** ^1^​ Integrated Research Facility at Fort Detrick (IRF-Frederick), Division of Clinical Research (DCR), National Institute of Allergy and Infectious Diseases (NIAID), National Institutes of Health (NIH), B-8200 Research Plaza, Fort Detrick, Frederick, MD 21702, USA; ^2^​ Ornamental Plants and Vegetables Research Center, Agricultural Research Department, Hokkaido Research Organization, Takikawa, Hokkaido, Japan; ^3^​ United States Department of Agriculture, Agricultural Research Service, US Horticultural Research Laboratory, Fort Pierce, FL, USA; ^4^​ D.I. Ivanovsky Institute of Virology of N.F. Gamaleya National Center on Epidemiology and Microbiology of Ministry of Health of Russian Federation, Moscow, Russia; ^5^​ Institute of Microbiology and Immunology, Faculty of Medicine, University of Ljubljana, Ljubljana, Slovenia; ^6^​ Centro de Biotecnología y Genómica de Plantas; Departamento de Biotecnología-Biología Vegetal, Instituto Nacional de Investigación y Tecnología Agraria y Alimentaria (INIA/CSIC), Campus de Montegancedo, Pozuelo de Alarcón; Escuela Técnica Superior de Ingeniería Agronómica, Alimentaria y de Biosistemas, Universidad Politécnica de Madrid (UPM), Madrid, Spain; ^7^​ Center for Ecology of Infectious Diseases, Department of Infectious Diseases, Department of Epidemiology and Biostatistics, Insitute of Bioinformatics, University of Georgia, Athens, GA, USA; ^8^​ Friedrich-Loeffler-Institut, Institute of Novel and Emerging Infectious Diseases, Greifswald-Insel Riems, Greifswald, Germany; ^9^​ Department of Biological Sciences, Mississippi State University, Starkville, MS,, Mississippi State, USA; ^10^​ Division of Plant Pathology, ICAR-Indian Agricultural Research Institute, New Delhi, India; ^11^​ Institute of Diagnostic Virology, Friedrich-Loeffler-Institut, Greifswald-Insel Riems, Germany; ^12^​ UFyMA, INTA-CONICET, Córdoba, Argentina; ^13^​ Division of High-Consequence Pathogens and Pathology, Viral Special Pathogens Branch, Centers for Disease Control and Prevention, Atlanta, GA, USA; ^14^​ Institute of Virology, Philipps-University Marburg, Marburg, Germany; ^15^​ Department of Microbiology, Immunology and Pathology, Colorado State University, Fort Collins, CO, USA; ^16^​ Commonwealth Scientific and Industrial Research Organisation (CSIRO), Australian Centre for Disease Preparedness, Geelong, VIC, Australia; ^17^​ Virology-Phytoplasmology Laboratory, Agroscope, 1260 Nyon, Switzerland; ^18^​ University of New Mexico Health Sciences Center, Albuquerque, NM, USA; ^19^​ Center for Infection and Immunity, and Department of Epidemiology, Mailman School of Public Health, Columbia University, New York, USA; ^20^​ French Agency for Food, Environmental and Occupational Heath Safety ANSES, Laboratory of Ploufragan-Plouzané-Niort, Ploufragan, France; ^21^​ RNA Viruses Section, Laboratory of Infectious Diseases, National Institute of Allergy and Infectious Diseases, National Institutes of Health, Bethesda, MD, USA; ^22^​ Department of Molecular Biology and Biochemistry, University of California, Irvine, CA, USA; ^23^​ The University of Texas Medical Branch at Galveston, Galveston, TX, USA; ^24^​ Division of Virology, National Health Laboratory Service and Division of Virology, University of the Free State, Bloemfontein, Bloemfontein, South Africa; ^25^​ Division Phytomedicine, Faculty of Life Sciences, Humboldt-Universität zu Berlin, Berlin, Germany; ^26^​ Colorado State University, Fort Collins, CO, USA; ^27^​ National Citrus Engineering and Technology Research Center, Citrus Research Institute, Southwest University, Beibei, Chongqing, PR China; ^28^​ Respiratory Virus and Influenza Unit, National Microbiology Center, Instituto de Salud Carlos III, Madrid, Spain; ^29^​ Department of Microbiology and Immunology, Albert Einstein College of Medicine, Bronx, NY, USA; ^30^​ Unite des Virus Emergents (Aix-Marseille Univ-IRD 190-Inserm 1207), Marseille, France; ^31^​ Centre for Agricultural Bioinformatics, ICAR-Indian Agricultural Statistics Research Institute, New Delhi, India; ^32^​ The New Zealand Institute for Plant and Food Research Limited, Auckland, New Zealand; ^33^​ Integrated Research Facility at Fort Detrick, National Institute of Allergy and Infectious Diseases, National Institutes of Health, Frederick, MD, USA; ^34^​ CIDEFI. Facultad de Ciencias Agrarias y Forestales, Universidad de La Plata, La Plata, Argentina; ^35^​ Department of Immunology and Microbiology IMM-6, The Scripps Research Institute, La Jolla, CA, USA; ^36^​ World Reference Center for Emerging Viruses and Arboviruses and Department of Microbiology and Immunology, The University of Texas Medical Branch at Galveston, Galveston, TX, USA; ^37^​ Department of Virology, Wageningen Bioveterinary Research, Lelystad, Netherlands; ^38^​ Instituto de Patología Vegetal, Centro de Investigaciones Agropecuarias, Instituto Nacional de Tecnología Agropecuaria (IPAVE-CIAP-INTA), Consejo Nacional de Investigaciones Científicas y Técnicas, Unidad de Fitopatología y Modelización Agrícola, Córdoba, Argentina; ^39^​ UMR 1161 Virology ANSES/INRAE/ENVA, ANSES Animal Health Laboratory, Maisons-Alfort, France; ^40^​ United States Army Medical Research Institute of Infectious Diseases, Fort Detrick, Frederick, MD, USA; ^41^​ Istituto per la Protezione Sostenibile delle Piante, Consiglio Nazionale delle Ricerche, Bari, Italy; ^42^​ Queensland Alliance for Agriculture and Food Innovation, The University of Queensland, St. Lucia, QLD, Australia; ^43^​ CIHEAM, Istituto Agronomico Mediterraneo di Bari, Valenzano, Italy; ^44^​ Institute of Virology, Charité-Universitätsmedizin Berlin, corporate member of Freie Universität Berlin, Humboldt-Universität zu Berlin, Berlin, Germany; ^45^​ School of Medicine, University of Pittsburgh, Pittsburgh, PA, USA; ^46^​ Robert Koch Institut, Berlin, Germany; ^47^​ School of Life Sciences, University of Warwick, Coventry, UK; ^48^​ CIHEAM, Istituto Agronomico Mediterraneo di Bari, Valenzano, Italy; ^49^​ Department of Medical Microbiology, Virology Unit, Hacettepe University Faculty of Medicine, Ankara, Turkey; ^50^​ Walter Reed Biosystematics Unit (WRBU), Smithsonian Institution, Museum Support Center, Suitland, MD, USA; ^51^​ One Health Branch, Walter Reed Army Institute of Research (WRAIR), Silver Spring, MD, USA; ^52^​ Department of Entomology, Smithsonian Institution–National Museum of Natural History (NMNH), Washington, DC, USA; ^53^​ China National Rice Research Institute, Hangzhou, PR China; ^54^​ Department of Pathology, University of Cambridge, Cambridge, UK; ^55^​ Animal and Plant Health Agency, Weybridge, Surrey, UK; ^56^​ World Health Organization, Geneva, Switzerland; ^57^​ Embrapa Cassava and Fruits, Cruz das Almas, Bahia, Brazil; ^58^​ Institute of Biochemistry and Biotechnology, Martin Luther University Halle-Wittenberg, Halle/Saale, Germany; ^59^​ Instituto de Biotecnología y Biología Molecular, Facultad de Ciencias Exactas, CONICET UNLP, La Plata, Argentina; ^60^​ Icahn School of Medicine at Mount Sinai, New York, NY, USA; ^61^​ United States Army Medical Research Institute of Infectious Diseases, Fort Detrick, Frederick, MD, USA; ^62^​ Brandenburg State Office of Rural Development, Agriculture and Land Consolidation (LELF), Frankfurt, Germany; ^63^​ Division Phytomedicine, Thaer-Institute of Agricultural and Horticultural Sciences, Humboldt-Universität Zu Berlin, Berlin, Germany; ^64^​ State Key Laboratory for Zoonotic Diseases, Key Laboratory of Zoonoses Research, Ministry of Education, College of Veterinary Medicine, Jilin University, Changchun, PR China; ^65^​ Department of Microbiology and Immunology, Division of Biomedical Graduate Research Organization, School of Medicine, Georgetown University, Washington, DC, USA; ^66^​ Institute of Vertebrate Biology of the Czech Academy of Sciences, Brno, Czechia; ^67^​ Department of Virology, Immunology and Microbiology, Chobanian and Avedisian School of Medicine; National Emerging Infectious Diseases Laboratories, Boston University, Boston, MA, USA; ^68^​ Institute of Novel and Emerging Infectious Diseases, Friedrich-Loeffler-Institut, Greifswald-Insel Riems, Germany; ^69^​ Division Phytomedicine, Thaer-Institute of Agricultural and Horticultural Sciences, Humboldt-Universität Zu Berlin, Berlin, Germany; ^70^​ Department of Virology, WHO Collaborating Centre for Arboviruses and Hemorrhagic Fever Reference and Research, Bernhard-Nocht Institute for Tropical Medicine, Hamburg, Germany; ^71^​ United States Department of Agriculture, Agricultural Research Service, USNA, Floral and Nursery Plants Research Unit, Beltsville, MD, USA; ^72^​ Department of Clinical Plant Science, Hosei University, Koganei, Tokyo, 184-8584, Japan; ^73^​ Kochi Agricultural Research Center, Nankoku, Kochi, Japan; ^74^​ Department of Virology, University of Helsinki, Medicum, Helsinki, Finland; ^75^​ Institute of Veterinary Pathology, Vetsuisse Faculty, University of Zurich, Zurich, Switzerland; ^76^​ The School of Science, Auckland University of Technology, Auckland, New Zealand; ^77^​ Department of Infectious Diseases, Yamagata University Faculty of Medicine, Yamagata, Japan; ^78^​ Graduate School of Veterinary Science, Osaka Metropolitan University; International Research Center for Infectious Diseases, Osaka Metropolitan University, Izumisano, Osaka, Japan; ^79^​ Centers for Disease Control and Prevention, Fort Collins, CO, USA; ^80^​ Department of Virology, Immunology and Microbiology, Chobanian and Avedisian School of Medicine; National Emerging Infectious Diseases Laboratories, Boston University, Boston, MA, USA; ^81^​ School of Veterinary Medicine, Murdoch University, Murdoch, WA, Australia; ^82^​ Graduate School of Agricultural Science, Kobe University, Kobe, Hyogo, Japan; ^83^​ State Key Laboratory of Agricultural Microbiology, Huazhong Agricultural University, Wuhan, Hubei Province, PR China; ^84^​ International Rice Research Institute, College, Los Baños, 4032, Laguna, Philippines; ^85^​ Institute of Virology, Charité-Universitätsmedizin Berlin, Corporate Member of Freie Universität Berlin, Humboldt-Universität zu Berlin, and Berlin Institute of Health, Charitéplatz 1, Berlin 10117, Germany; ^86^​ Institute of Virology, Biomedical Research Center, Slovak Academy of Sciences, Bratislava, Slovakia; ^87^​ Department of Biomedical and Clinical Sciences, Linköping University, Linköping, Sweden; ^88^​ Institute of Plant Science and Resources, Okayama University, Kurashiki, Japan; ^89^​ National Center for Biotechnology Information, National Library of Medicine, National Institutes of Health, Bethesda, MD, USA; ^90^​ Institut Pasteur, Université Paris Cité, CNRS UMR6047, Archaeal Virology Unit, Paris, France; ^91^​ Institute for Plant Protection, NARO, Tsukuba, Ibaraki, Japan; ^92^​ US Geological Survey Western Fisheries Research Center, Seattle, Washington, USA; ^93^​ KU Leuven, Rega Institute, Zoonotic Infectious Diseases unit; Department of Laboratory Medicine, University Hospitals Leuven, Leuven, Belgium; ^94^​ Centers for Disease Control and Prevention, Fort Collins, CO, USA; ^95^​ Department of Veterinary Biosciences, College of Veterinary Medicine, The Ohio State University, Columbus, OH, USA; ^96^​ Institute of Plant Virology, Ningbo University, Ningbo, PR China; ^97^​ Illumina (China), Beijing, PR China; ^98^​ Department of Pharmacology and Toxicology, School of Medicine, and the Center for Predictive Medicine for Biodefense and Emerging Infectious Diseases, University of Louisville, Louisville, KY, USA; ^99^​ The New Zealand Institute for Plant and Food Research Limited; School of Biological Sciences, The University of Auckland, Auckland, New Zealand; ^100^​ KU Leuven, Rega Institute, Zoonotic Infectious Diseases unit, Leuven, Belgium; ^101^​ FIND, the global alliance for diagnostics, Geneva, Switzerland; ^102^​ Instituto de Biología-Laboratorio de Genética Molecular-Pontificia Universidad Católica de ValparaísoCampus Curauma, Valparaíso, Chile; ^103^​ United States Department of Agriculture, Agricultural Research Service, Toledo, OH, USA; ^104^​ Worldwide Influenza Centre, Francis Crick Institute, London, UK; ^105^​ Folkhalsomyndigheten, Stockholm, Sweden; ^106^​ Department of Virology, Immunology and Microbiology, Chobanian and Avedisian School of Medicine; National Emerging Infectious Diseases Laboratories, Boston University, Boston, MA, USA; ^107^​ Department of Agriculture, Yamagata University, Tsuruoka, Japan; ^108^​ Department of Plant Pathology, Irrigated Agricultural Research and Extension Center, Washington State University, Prosser, WA, USA; ^109^​ Utsunomiya University, Utsunomiya, Japan; ^110^​ Istituto per la Protezione Sostenibile delle Piante, Consiglio Nazionale delle Ricerche, Bari, Italy; ^111^​ Instituto de Biología Molecular y Celular de Plantas, Universitat Politècnica de València-Consejo Superior de Investigaciones Científicas, Valencia, Spain; ^112^​ School of Agriculture, Utsunomiya University, Utsunomiya, Japan; ^113^​ Novosibirsk State University, Novosibirsk Oblast, Russia; ^114^​ Department of Pathobiological Sciences, Influenza Research Institute, University of Wisconsin-Madison, Madison, USA; ^115^​ Institute of Virology, University of Veterinary Medicine Vienna, Vienna, Austria; ^116^​ College of Medicine, Mohammed Bin Rashid University of Medicine and Health Sciences, Dubai, United Arab Emirates; ^117^​ Evandro Chagas Institute, Ananindeua, Pará, Brazil; ^118^​ Institute for Biosecurity and Microbial Forensics. Stillwater, Oklahoma State University, Oklahoma, USA; ^119^​ Kochi Agricultural Research Center, Nankoku, Kochi, Japan; ^120^​ Department of Microbiology, Icahn School of Medicine at Mount Sinai, New York, NY, USA; ^121^​ Instituto de Biología Molecular y Celular de Plantas (IBMCP), Consejo Superior de Investigaciones Cientificas-Universitat Politècnica de Valencia, Valencia, Spain; ^122^​ National Reference Centre for Arboviruses and Haemorrhagic Fever viruses, Department of Microbiology, Medical School, Aristotle University of Thessaloniki, Thessaloniki, Greece; ^123^​ Genome Competence Center (MF1), Robert Koch Institute, 13353 Berlin, Germany; ^124^​ College of Veterinary Medicine, Baker Institute for Animal Health, Cornell University, Ithaca, NY, USA; ^125^​ Departamento de Veterinária, Universidade Federal de Viçosa, Viçosa, MG, Brazil; ^126^​ Center for Emerging Zoonotic and Parasitic Diseases, National Institute for Communicable Diseases of the National Health Laboratory Service, Sandringham-Johannesburg, Gauteng, South Africa; ^127^​ Department of Population Health, College of Veterinary Medicine, University of Georgia, Athens, GA, USA; ^128^​ Institute of Diagnostic Virology, Friedrich-Loeffler-Institut, Greifswald-Insel Riems, Germany; ^129^​ Center for Translational Antiviral Research, Georgia State University Institute for Biomedical Sciences, Atlanta, GA, USA; ^130^​ Vaccine Design and Development Laboratory, International AIDS Vaccine Initiative, Brooklyn, NY, USA; ^131^​ The New Zealand Institute for Plant and Food Research Limited; The School of Science, Auckland University of Technology, Auckland, New Zealand; ^132^​ Division of Antivirals, Office of Infectious Diseases, Center for Drug Evaluation and Research, Food and Drug Administration, Silver Spring, MD, USA; ^133^​ Instituto Biológico de São Paulo, São Paulo, SP, Brazil; ^134^​ Division Phytomedicine, Faculty of Life Sciences, Humboldt-Universität zu Berlin, Berlin, Germany; ^135^​ Departamento de Biologia Celular, Universidade de Brasília, Brasília, Brazil; ^136^​ Instituto de Biotecnología y Biología Molecular, CONICET-UNLP, Facultad de Ciencias Exactas, Universidad Nacional de La Plata, Buenos Aires, Argentina; ^137^​ Department of Infectious Diseases and Immunology, College of Veterinary Medicine, University of Florida, Gainesville, Florida, USA; ^138^​ Instituto de Biotecnología y Biología Molecular, CONICET-UNLP, Facultad de Ciencias Exactas, Universidad Nacional de La Plata, Buenos Aires, Argentina; ^139^​ Institute of Diagnostic Virology, Friedrich-Loeffler-Institut, Greifswald-Insel Riems, Germany; ^140^​ Istituto per la Protezione Sostenibile delle Piante, Consiglio Nazionale delle Ricerche, Bari, Italy; ^141^​ Department of Infectious Disease & Global Health, Tufts University Cummings School of Veterinary Medicine, North Grafton, MA, USA; ^142^​ Department of Biochemistry, Molecular Biology, Entomology and Plant Pathology, Mississippi State University, Mississippi, Mississippi State, USA; ^143^​ Sydney Institute for Infectious Diseases, School of Medical Sciences, The University of Sydney, Sydney, Australia; ^144^​ Department of Veterinary Medicine, University of Maryland, College Park, MD 20742, USA; ^145^​ Institute for Plant Protection, National Agriculture and Food Research Organization, Tsukuba, Japan; ^146^​ Faculty of Medicine, University Medical Center-University Freiburg, Freiburg, Germany; ^147^​ Hawkesbury Institute for the Environment, Western Sydney University, Sydney, NSW, Australia; ^148^​ Sun Yat-sen University, Shenzhen, PR China; ^149^​ Kochi Agricultural Research Center, Nankoku, Kochi, Japan; ^150^​ Division of Genetics and Tree Improvement, Institute of Forest Biodiversity (ICFRE), Hyderabad, India; ^151^​ Bioinformatics Unit, Scientific Institute IRCCS “E. Medea”, Bosisio Parini, Italy; ^152^​ CBR Division, Dstl, Porton Down, Salisbury, Wiltshire, UK; ^153^​ Department of Microbiology, College of Medicine, Korea University, Seoul, Republic of Korea; ^154^​ School of Biomedical Sciences, Faculty of Health, Queensland University of Technology, Brisbane, QLD, Australia; ^155^​ Viral Special Pathogens Branch, Division of High-Consequence Pathogens and Pathology, Centers for Disease Control and Prevention, Atlanta, GA, USA; ^156^​ Department of Microbiology, Immunology, and Pathology, College of Veterinary Medicine and Biomedical Sciences, Colorado State University, Fort Collins, CO, USA; ^157^​ Division of Global Epidemiology, International Institute for Zoonosis Control, Hokkaido University, Sapporo, Japan; ^158^​ Institute for Plant Protection, NARO, Tsukuba, Ibaraki, Japan; ^159^​ Faculty of Agricultural Production and Management, Shizuoka Professional University of Agriculture, Shizuoka, Japan; ^160^​ The University of Texas Medical Branch at Galveston, Galveston, TX, USA; ^161^​ Centers for Disease Control and Prevention, Atlanta, GA, USA; ^162^​ National Citrus Engineering and Technology Research Center, Citrus Research Institute, Southwest University, Beibei, Chongqing, PR China; ^163^​ Laboratorio de Virología Molecular, Centro Ciencia & Vida, Fundación Ciencia & Vida and Facultad de Medicina y Ciencia, Universidad San Sebastián, Santiago, Chile; ^164^​ Institute for Plant Protection, National Agriculture and Food Research Organization, Tsukuba, Japan; ^165^​ Institute for Life and Medical Sciences (LiMe), Kyoto University, Kyoto, Japan; ^166^​ Institut Pasteur de Guinée, BP 4416, Conakry, Guinea; ^167^​ College of Veterinary Medicine, Jiangsu Co-innovation Center for Prevention and Control of Important Animal Infectious Diseases and Zoonoses, Yangzhou University, Yangzhou, PR China; ^168^​ Changchun Veterinary Research Institute, Chinese Academy of Agricultural Sciences, Changchun, PR China; ^169^​ Institute for Sustainable Plant Protection, National Research Council of Italy (IPSP-CNR), Torino, Italy; ^170^​ Department of Entomology and Plant Pathology, Division of Agriculture, University of Arkansas System, Fayetteville, AR, USA; ^171^​ Institute for Sustainable Plant Protection, National Research Council of Italy (IPSP-CNR), Torino, Italy; ^172^​ Department of Viroscience, Erasmus MC, University Medical Centre Rotterdam, Rotterdam, Netherlands; ^173^​ Department of Microbiology, Immunology and Transplantation, KU Leuven, Leuven, Belgium; ^174^​ The University of Texas Medical Branch at Galveston, Galveston, TX,, USA; ^175^​ Wageningen University and Research, Biointeractions and Plant Health, Wageningen, Netherlands; ^176^​ Division Phytomedicine, Faculty of Life Sciences, Humboldt-Universität zu Berlin, Berlin, Germany; ^177^​ Integrated Research Facility at Fort Detrick, National Institute of Allergy and Infectious Diseases, National Institutes of Health, Frederick, MD, USA; ^178^​ National Biodefense Analysis and Countermeasures Center, Fort Detrick, Frederick, MD, USA; ^179^​ School of Chemistry and Molecular Biosciences, University of Queensland, St. Lucia, QLD, Australia; ^180^​ Department of Veterinary Microbiology and Pathology, Washington State University, Pullman, WA, USA; ^181^​ Department of Entomology and Plant Pathology, North Carolina State University, Raleigh, NC, USA; ^182^​ National Center for Biotechnology Information, National Library of Medicine, National Institutes of Health, Bethesda, MD, USA; ^183^​ Key Laboratory of Special Pathogens and Biosafety, Wuhan Institute of Virology, Chinese Academy of Sciences, Wuhan, PR China; ^184^​ Plant Pathology Laboratory, Department of Crop Science, School of Agricultural Production, Infrastructure and Environment, Agricultural University of Athens, Votanikos, Athens, Greece; ^185^​ Yokohama Plant Protection Station, Yokohama, Kanagawa, Japan; ^186^​ Kochi Agricultural Research Center, Nankoku, Kochi, Japan; ^187^​ Institute of Insect Sciences, Zhejiang University, Hangzhou, PR China; ^188^​ Key Laboratory of Special Pathogens and Biosafety, Wuhan Institute of Virology, Chinese Academy of Sciences, Wuhan, PR China; ^189^​ Dep. de Fitopatologia/BIOAGRO, Universidade Federal de Viçosa, Viçosa, MG, Brazil; ^190^​ Center for Disease Control and Prevention of Xinjiang Military Command Area, Urumqi, Xinjiang, PR China; ^191^​ National Citrus Engineering and Technology Research Center, Citrus Research Institute, Southwest University, Beibei, Chongqing, PR China; ^192^​ Guangxi Academy of Specialty Crops, Guilin, Guangxi, PR China; ^193^​ School of Life Sciences and Human Phenome Institute, Fudan University, Shanghai, PR China; ^194^​ Key Laboratory of Special Pathogens and Biosafety, Wuhan Institute of Virology, Chinese Academy of Sciences, Wuhan, PR China; ^195^​ University of Chinese Academy of Sciences, Beijing, PR China; ^196^​ Pharmaq Analytiq, Bergen, Norway

**Keywords:** *Aliusviridae*, *Arenaviridae*, articulaviral, *Articulavirales*, *Artoviridae*, *Aspiviridae*, *Bornaviridae*, bunyaviral, *Bunyavirales*, bunyavirus, *Crepuscuviridae*, *Discoviridae*, *Filoviridae*, *Fimoviridae*, goujianviral, *Goujianvirales*, *Hantaviridae*, ICTV, International Committee on Taxonomy of Viruses, *Jingchuvirales*, *Lispiviridae*, megaclassification, megataxonomy, mononegaviral, *Mononegavirales*, muviral, *Muvirales*, *Mymonaviridae*, *Myriaviridae*, *Nairoviridae*, *Natareviridae*, negarnaviricot, *Negarnaviricota*, *Nyamiviridae*, *Orthomyxoviridae*, *Orthornavirae*, *Paramyxoviridae*, *Peribunyaviridae*, *Phasmaviridae*, *Phenuiviridae*, *Pneumoviridae*, *Rhabdoviridae*, *Riboviria*, serpentoviral, *Serpentovirales*, *Sunviridae*, *Tenuivirus*, *Tosoviridae*, *Tospoviridae*, *Tulasviridae*, virus classification, virus nomenclature, virus taxonomy

## Abstract

In April 2023, following the annual International Committee on Taxonomy of Viruses (ICTV) ratification vote on newly proposed taxa, the phylum *Negarnaviricota* was amended and emended. The phylum was expanded by one new family, 14 new genera, and 140 new species. Two genera and 538 species were renamed. One species was moved, and four were abolished. This article presents the updated taxonomy of *Negarnaviricota* as now accepted by the ICTV.

## Authorship

There are 187 co-authors for this manuscript. Co-authors are people who wrote/submitted/revised taxonomic proposals (Taxo-Props) that ultimately were accepted by the ICTV, ICTV Study Group members that reviewed/requested revisions/voted on submitted TaxoProps, and ICTV members who reviewed/requested revisions/voted/ratified TaxoProps. Due to space restrictions the full list of author affiliations can be found in the online version of this article.

## Introduction

Phylum *Negarnaviricota* was established in 2019 by the International Committee on Taxonomy of Viruses (ICTV) for negative-sense RNA viruses encoding evolutionarily related RNA-directed RNA polymerases (RdRps). The phylum includes two subphyla, *Haploviricotina* and *Polyploviricotina*, for negative-sense RNA viruses that encode RdRps with or without mRNA capping activity, respectively. The two subphyla include four (*Chunqiuviricetes*, *Milneviricetes*, *Monjiviricetes*, and *Yunchangviricetes*) and two classes (*Ellioviricetes* and *Insthoviricetes*), respectively [1–4]. The phylum was last amended/emended in 2022 [5]. Here, we present the changes that were proposed to the phylum via official taxonomic proposals (TaxoProps) in 2022 and accepted by the ICTV in April 2023 [6]. These changes are now part of the official ICTV taxonomy [7, 8].

## Taxonomic changes in phylum *Negarnaviricota*

The phylum-associated ICTV Study Groups voted in favour of recommending the elimination of hyphens in all virus name abbrevations. This recommendation is followed throughout this article.

The phylum was expanded by one new family, *Tosoviridae*, currently not assigned to either of the two established subphyla. The family includes one new genus, *Fraservirus*, with one new species, *Fraservirus testudinis*, for turtle fraservirus 1 (TFV1) first isolated from diseased Florida softshell turtles (trionychid *Apalone ferox* (Schneider, 1783)) and later detected in Florida red-bellied cooters (emydid *Pseudemys nelsoni* Carr, 1938) and peninsula cooters (emydid *Pseudemys peninsularis* Carr, 1938) sampled in Florida, USA [9] (TaxoProp 2022.010M.A.Fraservirus_1nfam_1ngen_1nsp).

## Taxonomic changes in subphylum *Haploviricotina*

### Class *Monjiviricetes*, order *Jingchuvirales*

#### Family *Aliusviridae*

Genus *Ollusvirus* was expanded by three new species (TaxoProp 2021.015M.A.Jingchuvirales_2ngen_10nsp):

- *Ollusvirus nomadae*, for Nomada lathburiana mononega-like virus (NLmlV);
- *Ollusvirus oropsyllae*, for Oropsylla silantiewi mononega-like virus 2 (OSmlV2); and
- *Ollusvirus rhagoveliae*, for Rhagovelia obesa mononega-like virus (ROmlV), all discovered in high-throughput sequencing (HTS) data [10].

#### Family *Chuviridae*

The family was expanded by two new genera (TaxoProp 2021.015M.A.Jingchuvirales_2ngen_10nsp):

- *Rochuvirus*, including one new species, *Rochuvirus chalcocoris*, for Chalcocoris rutilans mononega-like virus 2 (CRmlV2) and
- *Vapochuvirus*, including one new species, *Vapochuvirus trialeurodis*, for Trialeurodes vaporariorum mononega-like virus 2 (TVmlV2), both discovered in HTS data [10].

Genus *Mivirus* was expanded by one new species, *Mivirus belostomatis*, for Belostoma flumineum mononega-like virus (BFmlV) discovered in HTS data (TaxoProp 2021.015M.A.Jingchuvirales_2ngen_10nsp).

Genus *Nigecruvirus* was expanded by one new species, *Nigecruvirus periplanetae*, for Periplaneta americana mononega-like virus (PAmlV) discovered in HTS data [10] (TaxoProp 2021.015M.A.Jingchuvirales_2ngen_10nsp).

Genus *Piscichuvirus* was expanded by one new species, *Piscichuvirus craterocephali*, for hardyhead chuvirus (HhCV) discovered by HTS in flyspecked hardyheads (atherinid *Craterocephalus stercusmuscarum* (Günther, 1867)) [11] (TaxoProp 2021.015M.A.Jingchuvirales_2ngen_10nsp).

Genus *Scarabeuvirus* was expanded by two new species (TaxoProp 2021.015M.A.Jingchuvirales_2ngen_10nsp):

- *Scarabeuvirus supellae* for Supella longipalpa mononega-like virus 2 (SLmlV2) and
- *Scarabeuvirus trichopriae* for Trichopria drosophilae mononega-like virus (TDmlV), both discovered in HTS data [10].

### Class *Monjiviricetes*, order *Mononegavirales*

#### Family *Filoviridae*

All non-binomial species names in the family were changed to binomials as required by the recently amended International Code of Virus Classification and Nomenclature (ICVCN) [12–14] (TaxoProp 2021.012M.A.Filoviridae_sprenamed). The genus names *Ebolavirus* and *Marburgvirus* were changed to *Orthoebolavirus* and *Orthomarburgvirus*, respectively (TaxoProp 2022.009M.A.Filoviridae_2genrenamed).

#### Family *Lispiviridae*

Genus *Acridvirus* was created to include one new species, *Acridvirus hangzhouense*, for Hángzhōu acrida cinerea lispivirus 1 (HzACLV1) discovered by HTS in oriental longheaded grasshoppers (acridid *Acrida cinerea* (Thunberg, 1815)) sampled in Hángzhōu (杭州市), Zhèjiāng Province (浙江省), China (TaxoProp 2022.012M.A.Lispiviridae_7ngen_11nsp) [unpublished; GenBank #MZ209654].

Genus *Aleyavirus* was created to include one new species, *Aleyavirus fuyangense*, for Bemisia tabaci arlivirus 1 (BtAV1) discovered by HTS in whiteflies (aleyrodid *Bemisia tabaci* (Gennadius, 1889)) sampled in Fùyáng (阜阳市), Ānhuī Province (安徽省), China [15] (TaxoProp 2022.012M.A.Lispiviridae_7ngen_11nsp).

Genus *Aleybvirus* was created to include one new species, *Aleybvirus fuyangense*, for Bemisia tabaci arlivirus 2 (BtAV2) discovered by HTS in whiteflies (aleyrodid *Bemisia tabaci* (Gennadius, 1889)) sampled in Fùyáng (阜阳市), Ānhuī Province (安徽省), China [15] (TaxoProp 2022.012M.A.Lispiviridae_7ngen_11nsp).

Genus *Aranavirus* was created and includes one new species, *Aranavirus guiyangense*, for Guìyáng lispivirus 1 (GyLV1) discovered by HTS in wasp spiders (araneid *Argiope bruennichi* (Scopoli, 1772)) sampled in Guìyáng (贵阳市), Guìzhōu Province (贵州省), China (TaxoProp 2022.012M.A.Lispiviridae_7ngen_11nsp) [unpublished; GenBank #MZ209771].

Genus *Aranbvirus* was created to include one new species, *Aranbvirus guiyangense*, for Guìyáng lispivirus 2 (GyLV2) discovered by HTS in wasp spiders (araneid *Argiope bruennichi* (Scopoli, 1772)) sampled in Guìyáng (贵阳市), Guìzhōu Province (贵州省), China (TaxoProp 2022.012M.A.Lispiviridae_7ngen_11nsp) [unpublished; GenBank #MZ209782].

Genus *Arlivirus* was expanded by two new species (TaxoProp 2022.012M.A.Lispiviridae_7ngen_11nsp):

- *Arlivirus hangzhouense* for Hángzhōu Scotinophara lurida lispivirus 1 (HzSLLV1) discovered by HTS in black rice bugs (pentatomid *Scotinophara lurida* (Burmeister, 1834)) sampled in Hángzhōu (杭州市), Zhèjiāng Province (浙江省), China [unpublished; GenBank #MZ209613] and
- *Arlivirus ningboense* for Nbu stink bug virus 1 (NbuSBV1) discovered by HTS in yellow marmorated stink bugs (pentatomid *Erthesina fullo* [16]) sampled in Níngbō (宁波市), Zhèjiāng Province (浙江省), China [16].

Genus *Avesvirus* was created to include one new species, *Avesvirus sinense*, for Arlivirus sp. virus (ALV) discovered by HTS in whiteflies (aleyrodid *Bemisia tabaci* (Gennadius, 1889)) sampled in Fùyáng (阜阳市), Ānhuī Province (安徽省), China [17] (TaxoProp 2022.012M.A.Lispiviridae_7ngen_11nsp).

Genus *Damravirus* was expanded by one new species, *Damravirus fushunense*, for Fǔshùn Ischnura senegalensis lispivirus 1 (FsISLV1) discovered by HTS in common bluetails (coenagrionid *Ischnura senegalensis* (Rambur, 1842)) sampled in Fǔshùn (抚顺市), Liáoníng Province (辽宁省), China (TaxoProp 2022.012M.A.Lispiviridae_7ngen_11nsp) [unpublished; GenBank #MZ210022].

Genus *Hemipvirus* was created to include two new species (TaxoProp 2022.012M.A.Lispiviridae_7ngen_11nsp):

- *Hemipvirus scuti* for Hángzhōu Eysarcoris guttigerus lispivirus 1 (HzEGLV1) discovered by HTS in two-spotted sesame bugs (pentatomid *Eysarcoris guttigerus* (Thunberg, 1783)) sampled in Hángzhōu (杭州市), Zhèjiāng Province (浙江省), China [unpublished; GenBank #MZ209652] and
- *Hemipvirus veri* for Hángzhōu Cletus punctiger lispivirus 1 (HzCPLV1) discovered by HTS in rice stink bugs (coreid *Cletus punctiger* (Dallas, 1852)) sampled in Hángzhōu (杭州市), Zhèjiāng Province (浙江省), China [unpublished; GenBank #MZ209674].

#### Family *Mymonaviridae*

Genus *Auricularimonavirus* was expanded by one new species, *Auricularimonavirus armillariae*, for Armillaria mellea negative strand RNA virus 2 (AmNSRV2) discovered by HTS in honey fungi (physalacriaceaen *Armillaria mellea* (Vahl) P.Kumm. (1871)) sampled in South Africa [18] (TaxoProp 2022.014M.A.Mymonaviridae_13nsp).

Genus *Hubramonavirus* was expanded by three new species (TaxoProp 2022.014M.A.Mymonaviridae_13nsp):

- *Hubramonavirus fusarii* for Fusarium oxysporum mymonavirus 1 (FoMyV1) discovered by HTS in an ascomycete (nectriaceaen *Fusarium oxysporum* Schlecht. emend. Snyder and Hansen) sampled in Luòhé (漯河市), Hénán Province (河南省), China [19];
- *Hubramonavirus golovinomycesae* for Golovinomyces cichoracearum GcM3_contig_4635, here renamed Golovinomyces cichoracearum negative-stranded RNA virus 1 (GcNSRV1), discovered by HTS in an ascomycete (erysiphaceaen *Erysiphe cichoracearum* (DC.) (1805)) sampled in Shady Grove, Maryland, USA [20]; and
- *Hubramonavirus vitis* for grapevine-associated mononega-like virus 3 (GaMLV3) discovered by HTS in common grape vine (vitaceaen *Vitis vinifera* L.) sampled in the Veneto region of Italy [21].

Genus *Lentimonavirus* was expanded by one new species, *Lentimonavirus armillariae*, for Armillaria mellea negative-stranded RNA virus 1 (AmNSRV1) discovered by HTS in honey fungi (physalacriaceaen *Armillaria mellea* (Vahl) P.Kumm. (1871)) sampled in South Africa [18] (TaxoProp 2022.014M.A.Mymonaviridae_13nsp).

Genus *Penicillimonavirus* was expanded by four new species (TaxoProp 2022.014M.A.Mymonaviridae_13nsp):

- *Penicillimonavirus alphaerysiphe* for Erysiphe necator associated negative-stranded RNA virus 2 (EnaNSRV2) discovered by HTS in an ascomycete (erysiphaceaen *Erysiphe necator* (Schwein.) Burrill) sampled in Spain [unpublished; GenBank #MN617061];
- *Penicillimonavirus betaerysiphe* for Erysiphe necator associated negative-stranded RNA virus 5 (EnaNSRV5) discovered by HTS in an ascomycete (erysiphaceaen *Erysiphe necator* (Schwein.) Burrill) sampled in Spain [unpublished; GenBank #MN617042];
- *Penicillimonavirus gammaerysiphe* for Erysiphe necator associated negative-stranded RNA virus 6 (EnaNSRV6) discovered by HTS in an ascomycete (erysiphaceaen *Erysiphe necator* (Schwein.) Burrill) sampled in Spain [unpublished; GenBank #MN617052]; and
- *Penicillimonavirus magnaporthe* for Magnaporthe oryzae mononegaambi virus 1 (MoMV1) discovered by HTS in rice blast fungi (magnaporthaceaen *Magnaporthe oryzae* (T.T. Hebert) M.E. Barr) sampled in the Philippines [unpublished; GenBank #MW752165].

Genus *Plasmopamonavirus* was expanded by one new species, *Plasmopamonavirus erysiphe*, for Erysiphe necator associated negative-stranded RNA virus 23 (EnaNSRV23) discovered by HTS in an ascomycete (erysiphaceaen *Erysiphe* necator (Schwein.) Burrill) sampled in Spain (TaxoProp 2022.014M.A.Mymonaviridae_13nsp) [unpublished; GenBank #MN617053].

Genus *Sclerotimonavirus* was expanded by three new species (TaxoProp 2022.014M.A.Mymonaviridae_13nsp):

- *Sclerotimonavirus asiafusarii* for Fusarium asiaticum negative-stranded RNA virus 1 (FaNSRV1) discovered by HTS in an ascomycete (nectriaceaen *Fusarium asiaticum* O’Donnell, Aoki, Kistler and Geiser) sampled in Yangtze Plain (长江中下游平原), China [22];
- *Sclerotimonavirus xinjiangense* for Xīnjiāng mymona-like virus 2 (XjMLV2) discovered by metagenomics of soil sampled in Xīnjiāng Uygur Autonomous Region (新疆维吾尔自治区), China [23]; and
- *Sclerotimonavirus yunnanense* for Yúnnán mymona-like virus 1 (YnMLV1) discovered by metagenomics of soil sampled in Yúnnán Province (云南省), China [23].

#### Family *Nyamiviridae*

Genus *Formivirus* was expanded by one new species, *Formivirus pollinis*, for Xiāngshān nyami-like virus (XNyV) discovered by metagenomics in wild insect pollinators in Xiāngshān Park (香山公园), Beijing, China [24] (TaxoProp 2022.016M.A.Nyamiviridae_2nsp).

Genus *Orinovirus* was expanded by one new species, *Orinovirus sanyae*, for Sanya nyamivirus 1 (SaNyV1; discouraged synonym: Hángzhōu nyamivirus 1) discovered by metagenomics in a beetle (staphylinid *Paederus fuscipes* Curtis, 1826) sampled in a rice field in China (TaxoProp 2022.016M.A.Nyamiviridae_2nsp) [unpublished; Genbank #MZ209903].

#### Family *Paramyxoviridae*

The names of all species in the family were changed to binomials as required by the recently amended ICVCN [12–14] (TaxoProp 2021.026M.A.Paramyxoviridae_sprename).

#### Family *Pneumoviridae*

The names of all species in the family were changed to binomials as required by the recently amended ICVCN [12–14] (TaxoProp 2021.033M.A.Pneumoviridae_sprename).

#### Family *Rhabdoviridae*

One new genus, *Thriprhavirus*, was created to include two new species (TaxoProp 2022.002M.A.Alpharhabdovirinae_1ngen14nsp):

- *Thriprhavirus intonsa* for Hangzhou Frankliniella intonsa rhabdovirus 1 (HFinRV1; discouraged synonym: Hubei dimarhabdovirus 4 (HbDRV4)) [25] discovered by metagenomics of European flower thrips (thripid *Frankliniella intonsa* (Trybom, 1895)) sampled in China in 2016 [unpublished; GenBank #MZ209657] and
- *Thriprhavirus tabaci* for Thrips tabaci associated dimarhabdovirus 1 (TtaDRV1) discovered by metagenomics of onion thrips (thripid *Thrips tabaci* Lindeman, 1889) sampled in Italy [26].

Genus *Almendravirus* was expanded by one new species, *Almendravirus xianshan*, for Xiangshan rhabdo-like virus 1 (XsRLV1) discovered by metagenomics of insects sampled in Beijing, China (TaxoProp 2022.002M.A.Alpharhabdovirinae_1ngen14nsp) [unpublished; GenBank #OK491499].

Genus *Alphanemrhavirus* was expanded by two new species (TaxoProp 2022.002M.A.Alpharhabdovirinae_1ngen14nsp):

- *Alphanemrhavirus bangkok* for Rattus tanezumi rhabdovirus 1 (RtaRV1) discovered by metagenomics of oriental house rats (murid *Rattus tanezumi* Temminck, 1844) sampled in Bangkok, Thailand [27] and
- *Alphanemrhavirus sodak* for Sodak rhabdovirus 1 (SDRV1) discovered by metagenomics of big brown bats (vespertilionid *Eptesicus fuscus* (Beauvois, 1796)) collected in South Dakota, USA [28].

Genus *Alphanucleorhabdovirus* was expanded by two new species (TaxoProp 2022.001M.A.Alpha and betanucleorhabdoviruses_6nsp):

- *Alphanucleorhabdovirus agavis*, for Agave tequilana virus 1 (ATV1) discovered by HTS in blue agave (asparagaceaen *Agave tequilana* F.A.C. Weber) sampled in Guanajuato, Mexico [29] and
- *Alphanucleorhabdovirus artemisiae* for Artemisia capillaris nucleorhabdovirus 1 (ArtCaNV1) discovered by HTS in capillary wormwood (*Artemisia capillaris* Thunberg) sampled from Kizu River (木津川), Kyoto Prefecture (京都府), Japan [30].

Genus *Alpharicinrhavirus* was expanded by one new species, *Alpharicinrhavirus hubei*, for Hubei tick rhabdovirus 1 (HbTRV1) discovered by metagenomics of Asian longhorned ticks (ixodid *Haemaphysalis longicornis* Neumann, 1901) sampled in Húběi Province (湖北省), China [31] (TaxoProp 2022.002M.A.Alpharhabdovirinae_1ngen14nsp).

Genus *Betanucleorhabdovirus* was expanded by four new species (TaxoProp 2022.001M.A.Alpha and betanucleorhabdoviruses_6nsp):

- *Betanucleorhabdovirus asclepiadis* for Asclepias syriaca virus 2 (AscSyV2) discovered by HTS in common milkweed (apocynaceaen *Asclepias syriaca* L.) sampled in Illinois, USA [29];
- *Betanucleorhabdovirus cnidii* for Cnidium virus 1 (CnV1) discovered by HTS in apiaceaen *Cnidium officinale* Makino plants sampled in South Korea [32];
- *Betanucleorhabdovirus plectranthi* for Plectranthus aromaticus virus 1 (PleArV1) discovered by HTS in Cuban oregano (lamiaceaen *Coleus amboinicus* Lour.) sampled in Malaysia [29]; and
- *Betanucleorhabdovirus rhododendri* for Rhododendon delavayi virus 1 (RhoDeV1) discovered by HTS in red rhododendron (ericaceaen *Rhododendron arboreum delavayi* Franchet) sampled in Yúnnán Province (云南省), China [29].

Genus *Cytorhabdovirus* was expanded by ten new species (TaxoProp 2022.007M.A.Cytorhabdovirus_10nsp):

- *Cytorhabdovirus actinidiae* for Actinidia virus D (AcVD) discovered by HTS in golden kiwifruit (actinidiaceaen *Actinidia chinensis* Planch.) sampled in Jiāngxī Province (江西省), China [33];
- *Cytorhabdovirus anthurii* for Anthurium amnicola virus 1 (AntAmV1) discovered by HTS in Hawaiian tulip (araceaen *Anthurium amnicola* Dressler) sampled in Hawaii, USA [29];
- *Cytorhabdovirus asclepiadis* for Asclepias syriaca virus 1 (AscSyV1) discovered by HTS in common milkweed (apocynaceaen *Asclepias syriaca* L.) sampled in Illinois, USA [29];
- *Cytorhabdovirus bemisiae* for Bemisia tabaci-associated virus 1 (BeTaV1) discovered by HTS in whiteflies (aleyrodid *Bemisia tabaci* (Gennadius, 1889)) sampled in India [29];
- *Cytorhabdovirus glehniae* for Glehnia littoralis virus 1 (GlLV1) discovered by HTS in beach silvertop (apiaceaen *Glehnia littoralis* F. Schmidt ex Miq.) sampled in Fújiàn Province (福建省), China [29];
- *Cytorhabdovirus kenyatuberosum* for Kenyan potato cytorhabdovirus (KePCyV) discovered by HTS in potatoes (solanaceaen *Solanum tuberosum* L.) sampled in Kenya [unpublished, GenBank #MN689395];
- *Cytorhabdovirus nymphaeae* for Nymphaea alba virus 1 (NymAV1) discovered by HTS in European white water lily (nymphaeaceaen *Nymphaea alba* L.) sampled in Turkey [29];
- *Cytorhabdovirus orchidaceae* for Gymnadenia densiflora virus 1 (GymDenV1) discovered by HTS in marsh fragrant orchid (orchidaceaen *Gymnadenia densiflora* (Wahlenb.) A.Dietr.) sampled in Switzerland [29];
- *Cytorhabdovirus tagetis* for Tagetes erecta virus 1 (TaEV1) discovered by HTS in African marigold (asteraceaen *Tagetes erecta* L.) sampled in Ānhuī Province (安徽省), China [29]; and
- *Cytorhabdovirus trachyspermi* for Trachyspermum ammi virus 1 (TrAV1) discovered by HTS in ajowan (apiaceaen *Trachyspermum ammi* L.) sampled in Tehran, Iran [29].

Genus *Ephemerovirus* was expanded by two new species (TaxoProp 2022.008M.A.Ephemerovirus_2nsp):

- *Ephemerovirus guangdong* for porcine ephemerovirus 2 (PoEV2) [34] and
- *Ephemerovirus henan* for porcine ephemerovirus 1 (PoEV1), both detected by HTS in domestic pigs (suid *Sus scrofa domesticus* Linnaeus, 1758) sampled in China [34].

Genus *Ledantevirus* was expanded by two new species (TaxoProp 2022.002M.A.Alpharhabdovirinae_1ngen14nsp):

- *Ledantevirus longquan* for Longquan Niviventer coninga ledantevirus 1 (LNcoLV1) discovered by metagenomics of Edward’s leopoldamys (murid *Leopoldamys edwardsi* (Thomas, 1882)) sampled in China [unpublished; GenBank #MZ328293] and
- *Ledantevirus wenzhou* for Wenzhou Rhinolophus pusillus ledantevirus 1 (WRpuLV1) discovered by metagenomics of least horseshoe bats (rhinolophid *Rhinolophus pusillus* Temminck, 1834) sampled in China [unpublished; GenBank #OM030289].

Genus *Sigmavirus* was expanded by three new species (TaxoProp 2022.002M.A.Alpharhabdovirinae_1ngen14nsp):

- *Sigmavirus sichuan* for Apis rhabdovirus 3 (ApRV3) discovered by metagenomics of eastern honey bees (apid *Apis cerana Fabricius*, 1793) sampled in Sìchuān Province (四川省), China [unpublished; GenBank: #MZ822104];
- *Sigmavirus tuva* for Aksy-Durug Melophagus sigmavirus (ADMSV) discovered by metagenomics of sheep keds (hippoboscid *Melophagus ovinus* (Linnaeus, 1758)) sampled in the Republic of Tuva (Республика Тыва), Russia [35]; and
- *Sigmavirus yushu* for Yushu rhabdovirus (YsRV) discovered by metagenomics of bird faeces sampled in the Yushu Tibetan Autonomous Prefecture (玉树藏族自治州), Qīnghǎi Province (青海省), China [unpublished; GenBank #MW826525].

Genus *Tibrovirus* was expanded by one new species, *Tibrovirus mundri*, for Mundri virus (MUNV) discovered by HTS in a human clinical sample from Mundri West County, Western Equatoria, South Sudan [36] (TaxoProp 2022.002M.A.Alpharhabdovirinae_1ngen14nsp).

Genus *Tupavirus* was expanded by two new species (TaxoProp 2022.002M.A.Alpharhabdovirinae_1ngen14nsp):

- *Tupavirus laniger* for Wenzhou Myotis laniger tupavirus 1 (WMlaTV1) discovered by metagenomics of Chinese water myotis (vespertilionid *Myotis laniger* Peters, 1871) sampled in China [unpublished; GenBank #OM030290] and
- *Tupavirus pearsonii* for Wufeng Rhinolophus pearsonii tupavirus 1 (WRpeTV1) discovered by metagenomics of Pearson’s horseshoe bats (*Rhinolophus pearsonii* Horsfield, 1851) sampled in China [unpublished; GenBank #MZ328291].

Genus *Varicosavirus* was expanded by nine new species (TaxoProp 2022.021M.A.Varicosavirus_9nsp):

- *Varicosavirus allii* for Allium angulosum virus 1 (AAnV1) discovered in transcriptome data of mouse garlic (amaryllidaceaen *Allium angulosum* L.) sampled in Brno, South Moravian Region (Jihomoravský kraj), Czech Republic [29];
- *Varicosavirus brassicae* for Brassica rapa virus 1 (BrRV1) discovered in transcriptome data of bok choy (brassicaceaen *Brassica rapa* L.) sampled in Guǎngdōng Province (广东省), China [29];
- *Varicosavirus ipomoeae* for morning glory varicosavirus (MGVV) discovered by HTS in tall morning glory (convolvulaceaen *Ipomoea purpurea* [L.] Roth) sampled in Hénán Province (河南省), China [37];
- *Varicosavirus lolii* for Lolium perenne virus 1 (LoPV1) discovered in transcriptome data of perennial ryegrass (poaceaen *Lolium perenne* L.) sampled in Store Heddinge, Region Zealand (Region Sjælland), Denmark [29];
- *Varicosavirus melampyri* for Melampyrum roseum virus 1 (MelRoV1) discovered in transcriptome data of Asian cow-wheat (oorobanchaceaen *Melampyrum roseum* Maxim) sampled in Kanagawa Prefecture (神奈川県), Japan [29];
- *Varicosavirus pini* for Pinus flexilis virus 1 (PiFleV1) discovered in transcriptome data of limber pine (pinaceaen *Pinus flexilis* E. James) sampled in Oregon, USA [29];
- *Varicosavirus vitis* for Vitis varicosavirus (VVV) discovered in by HTS in crimson glory vine (vitaceaen *Vitis coignetiae* Pulliat ex Planch.) sampled in Hokkaido (北海道), Japan [38];
- *Varicosavirus xinjiangense* for Xinjiang varicosavirus (XVV) identified by metagenomics of soil sampled in Xīnjiāng Uygur Autonomous Region (新疆维吾尔自治区), China [23]; and
- *Varicosavirus zosterae* for Zostera-associated varicosavirus 1 (ZaVV1) discovered in transcriptome data of common eelgrass (zosteraceaen *Zostera marina* L.) sampled in Shāndōng Province (山东省), China [39].

Genus *Vesiculovirus* was expanded by two new species (TaxoProp 2022.022M.A.Vesiculovirus_2nsp):

- *Vesiculovirus mejal* for Mejal virus (MEJV) discovered by metagenomics of streblid bat flies (*Trichobius* sp.) sampled in Villa Purificación, Jalisco, Mexico [40] and
- *Vesiculovirus wufeng* for Wufeng Myotis altarium vesiculovirus 1 (WMalVV1) discovered by metagenomics of Szechwan myotis (vespertilionid *Myotis altarium* Thomas, 1911) sampled in China [unpublished; GenBank #OM030292].

## Taxonomic changes in subphylum *Polyploviricotina*

### Class *Ellioviricetes*, order *Bunyavirales*

#### Family *Arenaviridae*

The names of all species in the family were changed to binomials as required by the recently amended ICVCN [12–14] (TaxoProp 2021.008M.A.Arenaviridae_rename).

The family was expanded by one genus, *Innmovirus*, including one new species, *Innmovirus hailarense*, for Inner Mongolia sediment arena-like virus (IMSV), here renamed Hailar virus (HLRV), discovered by HTS in river sediment sampled from Hailar River (海拉尔河), Inner Mongolia Autonomous Region (内蒙古自治区), China [23] (TaxoProp 2022.003M.A.Arenaviridae_1ngen_1nsp).

Genus *Hartmanivirus* was expanded by two new species, both discovered by HTS in boa constrictors (boid *Boa constrictor* Linnaeus, 1758) sampled in Costa Rica [41] (TaxoProp 2022.011M.A.Hartmanivirus_2nsp):

- *Hartmanivirus quadrati* for big electron-dense squares virus 1 (BESV1) and
- *Hartmanivirus unni* for Universidad Nacional virus 1 (UnNV1).

Genus *Mammarenavirus* was expanded by one new species, *Mammarenavirus dhati-welelense*, for Dhati Welel virus (DHWV) discovered by HTS in Awash mastomys (murid *Mastomys awashensis* Lavrenchenko, Likhnova & Baskevich, 1998) and Natal mastomys (murid *Mastomys natalensis* Smith, 1834) sampled in Dhati Welel (Dai/Dati Wolel) National Park, Ethiopia [42] (TaxoProp 2022.013M.A.Mammarenavirus_1nsp).

#### Family *Fimoviridae*

Genus *Emaravirus* was expanded by five new species:

- *Emaravirus corynocarpi* for karaka Ōkahu purepure emaravirus (KOPV) discovered by HTS in karaka trees (corynocarpaceaen *Corynocarpus laevigatus* J.R.Forst. & G.Forst., 1776) sampled in Auckland, New Zealand [43] (TaxoProp 2022.022P.A.Emaravirus_1ns);
- *Emaravirus fraxini* for ash shoestring-associated virus (ASaV) discovered by HTS in European ash (oleaceaen *Fraxinus excelsior* L.) and manna ash (*F. ornus* L.) sampled in Germany, Italy, Sweden, and Switzerland [44] (TaxoProp 2022.019P.A.Emaravirus_1ns);
- *Emaravirus illicii* for Japanese star anise ringspot-associated virus (JSARaV) discovered by HTS in Japanese star anise (schisandraceaen *Illicium anisatum* L.) sampled in Nakatosa (中土佐町), Kōchi Prefecture (高知県), Japan [45] (TaxoProp 2022.021P.A.Emaravirus_1ns);
- *Emaravirus visci* for Arceuthobium sichuanense-associated virus 1 (ArSaV1) discovered in transcriptome data of spruce dwarf mistletoe (santalaceaen *Arceuthobium sicuanense* (H.S. Kiu) D. Hawksw. and Wiens) sampled in China [46] (TaxoProp 2022.018P.A.Emaravirus_1ns); and
- *Emaravirus vitis* for Vitis emaravirus (VEV) discovered by HTS in crimson glory vine (vitaceaen *Vitis coignetiae* Pulliat ex Planch.) sampled in Hokkaido (北海道), Japan [38] (TaxoProp 2022.020P.A.Emaravirus_1ns).

#### Family *Hantaviridae*

The names of all species in the family were changed to binomials as required by the recently amended ICVCN [12–14] (TaxoProp 2021.013M.A.Hantaviridae_sprename).

#### Family *Nairoviridae*

The names of all species in the family were changed to binomials as required by the recently amended ICVCN [12–14] (TaxoProp 2021.017M.A.Nairoviridae_sprenamed).

Genus *Norwavirus* was expanded by one new species, *Norwavirus beijiense*, for Běijí nairovirus (BJNV; discouraged synonym: Gakugsa virus) discovered by HTS in taiga ticks (ixodid *Ixodes persulcatus* (Schulze, 1930)) sampled in Běijí (北极镇), Hēilóngjiāng Province (黑龙江省), China [47] (TaxoProp 2022.015M.A.Nairoviridae_4nsp).

Genus *Orthonairovirus* was expanded by three new species (TaxoProp 2022.015M.A.Nairoviridae_4nsp):

- *Orthonairovirus songlingense* for Sōnglǐng virus (SGLV) isolated from human clinical samples from Sōnglǐng District (松岭区), Hēilóngjiāng Province (黑龙江省), China [48];
- *Orthonairovirus sulinaense* for Sulina virus (SULV) discovered by HTS in castor bean ticks (ixodid *Ixodes ricinus* (Linnaeus, 1758)) sampled around Sulina, Northern Dobruja (Dobrogea de Nord), Romania [49]; and
- *Orthonairovirus yezoense* for Yezo virus (YEZV) isolated from human clinical samples from Hokkaidō (北海道), Japan [50].

#### Family *Peribunyaviridae*

The names of all species in the family were changed to binomials as required by the recently amended ICVCN [12–14] (TaxoProp 2021.028M.A.Peribunyaviridae_sprename).

Genus *Orthobunyavirus* was expanded by 30 new species:

- *Orthobunyavirus baakalense* for Baakal virus (BKAV) discovered by HTS in Florida SLE mosquitoes (culicid *Culex nigripalpus* Theobald, 1901) sampled in Palenque National Park, Mexico (TaxoProp 2022.018M.A.Orthobunyavirus_29nsp_abolish4sp) [51];
- *Orthobunyavirus balagoduense* for Balagodu virus (BLGV) discovered by HTS in paddybirds (ardeid Ardeola grayii (Sykes, 1832)) sampled in Balagodu, Karnataka, India [52] (TaxoProp 2022.018M.A.Orthobunyavirus_29nsp_abolish4sp);
- *Orthobunyavirus belemense* for Belém virus (BLMV) isolated from white-shouldered fire-eyes (thamnophilid *Pyriglena leucoptera* (Vieillot, 1818)) sampled in Belém, Pará Stae, Brazil [53, 54] (TaxoProp 2022.018M.A.Orthobunyavirus_29nsp_abolish4sp);
- *Orthobunyavirus benficaense* for Benfica virus (BENV=BNFV) moved from species *Bushbush orthobunyavirus*/*Orthobunyavirus bushbushense* (TaxoProp 2022.018M.A.Orthobunyavirus_29nsp_abolish4sp);
- *Orthobunyavirus bobayaense* for Bobaya virus (BOBV) isolated from Kurrichane thrushes (turdid *Turdus libonyana* (Smith, 1836)) sampled in M'Poko, Central African Republic [53, 55] (TaxoProp 2022.018M.A.Orthobunyavirus_29nsp_abolish4sp);
- *Orthobunyavirus boraceiaense* for Boracéia virus (BORV) moved from species *Anopheles B orthobunyavirus*/*Orthobunyavirus cuchillaense* (TaxoProp 2022.018M.A.Orthobunyavirus_29nsp_abolish4sp);
- *Orthobunyavirus ebiense* for Ebinur Lake virus (EBIV; discouraged synonym: Abbey Lake bunyavirus) isolated from mosquitoes (culicid *Culex modestus* Ficalbi, 1889) sampled in Ebi(nur) Lake (艾比湖), Xīnjiāng Uygur Autonomous Region (新疆维吾尔自治区), China [56, 57] (TaxoProp 2022.017M.A.Orthobunyavirus_1nsp);
- *Orthobunyavirus guaratubaense* for Guaratuba virus (GTBV) and Itimirim virus (ITIV) moved from species *Bertioga orthobunyavirus*/*Orthobunyavirus bertiogaense* (TaxoProp 2022.018M.A.Orthobunyavirus_29nsp_abolish4sp);
- *Orthobunyavirus gumbolimboense* for Gumbo Limbo virus (GLV) moved from species *Marituba orthobunyavirus*/*Orthobunyavirus maritubaense* (TaxoProp 2022.018M.A.Orthobunyavirus_29nsp_abolish4sp);
- *Orthobunyavirus heptayabaense* for Yaba-7 virus (Y7V) moved from species *Akabane orthobunyavirus*/*Orthobunyavirus akabaneense* (TaxoProp 2022.018M.A.Orthobunyavirus_29nsp_abolish4sp);
- *Orthobunyavirus infirmati* for Infirmatus virus (INFV) isolated from mosquitoes (culicid *Ochlerotatus infirmatus* (Dyar and Knab, 1906)) sampled in Tampa Bay Downs, Hillsborough County, Florida, USA [58] (TaxoProp 2022.018M.A.Orthobunyavirus_29nsp_abolish4sp);
- *Orthobunyavirus juandiazense* for Juan Díaz virus (JDV) moved from species *Bushbush orthobunyavirus*/*Orthobunyavirus bushbushense* (TaxoProp 2022.018M.A.Orthobunyavirus_29nsp_abolish4sp);
- *Orthobunyavirus ketapangense* for Ketapang virus (KETV) moved from species *Bakau orthobunyavirus*/*Orthobunyavirus bakauense* (TaxoProp 2022.018M.A.Orthobunyavirus_29nsp_abolish4sp);
- *Orthobunyavirus kowanyamaense* for Kowanyama virus (KOWV) isolated from mosquitoes (culicid *Anopheles annulipes* Walker, 1856) sampled in Mitchell River Mission, Queensland, Australia [59, 60] (TaxoProp 2022.018M.A.Orthobunyavirus_29nsp_abolish4sp);
- *Orthobunyavirus lasmaloyasense* for Las Maloyas virus (LMV) moved from species *Anopheles A orthobunyavirus*/*Orthobunyavirus horizonteense* (TaxoProp 2022.018M.A.Orthobunyavirus_29nsp_abolish4sp);
- *Orthobunyavirus ledniceense* for Lednice virus (LEDV) moved from species *Turlock orthobunyavirus*/*Orthobunyavirus turlockense* (TaxoProp 2022.018M.A.Orthobunyavirus_29nsp_abolish4sp);
- *Orthobunyavirus lukuniense* for Lukuni virus (LUKV) moved from species *Anopheles A orthobunyavirus*/*Orthobunyavirus horizonteense* (TaxoProp 2022.018M.A.Orthobunyavirus_29nsp_abolish4sp);
- *Orthobunyavirus mirimense* for Mirim virus (MIRV) moved from species *Bertioga orthobunyavirus*/*Orthobunyavirus bertiogaense* (TaxoProp 2022.018M.A.Orthobunyavirus_29nsp_abolish4sp);
- *Orthobunyavirus nepuyoi* for Nepuyo virus (NEPV) moved from species *Marituba orthobunyavirus*/*Orthobunyavirus maritubaense* (TaxoProp 2022.018M.A.Orthobunyavirus_29nsp_abolish4sp);
- *Orthobunyavirus nesszionaense* for Ness Ziona virus (NZV) discovered by HTS in a horse sampled in Israel [61] (TaxoProp 2022.018M.A.Orthobunyavirus_29nsp_abolish4sp);
- *Orthobunyavirus nolaense* for Nola virus (NOLAV) moved from species *Bakau orthobunyavirus*/*Orthobunyavirus bakauense* (TaxoProp 2022.018M.A.Orthobunyavirus_29nsp_abolish4sp);
- *Orthobunyavirus northwayense* for Northway virus (NORV) moved from species *Bunyamwera orthobunyavirus*/*Orthobunyavirus bunyamweraense* (TaxoProp 2022.018M.A.Orthobunyavirus_29nsp_abolish4sp);
- *Orthobunyavirus okolaense* for Okola virus (OKOV) isolated from mosquitoes (culicid *Eretmapodites chrysogaster* Graham, 1909) sampled in Okola, Cameroon [53, 62] (TaxoProp 2022.018M.A.Orthobunyavirus_29nsp_abolish4sp);
- *Orthobunyavirus pacoraense* for Pacora virus (PCAV) discovered by HTS in mosquitoes (culicid *Culex dunni* Dyar, 1918) sampled in Lambayeque Province, Panama [53, 63] (TaxoProp 2022.018M.A.Orthobunyavirus_29nsp_abolish4sp);
- *Orthobunyavirus sanjuanense* for San Juan virus (SJV) moved from species *Alajuela orthobunyavirus* (TaxoProp 2022.018M.A.Orthobunyavirus_29nsp_abolish4sp);
- *Orthobunyavirus shokweense* for Shokwe virus (SHOV) moved from species *Bunyamwera orthobunyavirus*/*Orthobunyavirus bunyamweraense* (TaxoProp 2022.018M.A.Orthobunyavirus_29nsp_abolish4sp);
- *Orthobunyavirus tangaense* for Tanga virus (TANV) isolated from mosquitoes (culicid *Anopheles funestus* Giles, 1900) sampled in Tanga, Tanzania [53, 64, 65] (TaxoProp 2022.018M.A.Orthobunyavirus_29nsp_abolish4sp);
- *Orthobunyavirus telokense* for Telok Forest virus (TFV) moved from species *Bakau orthobunyavirus*/*Orthobunyavirus bakauense* (TaxoProp 2022.018M.A.Orthobunyavirus_29nsp_abolish4sp);
- *Orthobunyavirus termeilense* for Termeil virus (TERV) isolated from mosquitoes (culicid *Aedes camptorhynchus* Thomson, 1868; culicid *Ochlerotatus vigilax* (Skuse, 1889)); and culicid *Culex annulirostris* Skuse, 1889) sampled in Termeil State Forest, New South Wales, Australia [53, 66] (TaxoProp 2022.018M.A.Orthobunyavirus_29nsp_abolish4sp); and
- *Orthobunyavirus umbreense* for Umbre virus (UMBV) moved from species *Turlock orthobunyavirus*/*Orthobunyavirus turlockense* (TaxoProp 2022.018M.A.Orthobunyavirus_29nsp_abolish4sp).

Seven previously unclassified viruses were classified into established orthobunyavirus species (TaxoProp 2022.018M.A.Orthobunyavirus_29nsp_abolish4sp):

- Antequera virus (ANTV), Barranqueras virus (BQSV), and Resistencia virus (RTAV), all isolated from mosquitoes (culicid *Culex delpontei* Duret, 1969) sampled in Chaco Province, Argentina [53, 67], were classified into species *Bellavista orthobunyavirus*/*Orthobunyavirus bellavistaense*;
- Laguna Larga virus (LLV), isolated from mosquitoes (culicid *Ochlerotatus albifasciatus* (Macquart, 1838)) sampled in Córdoba Province, Argentina [68, 69], was classified into species *Fort Sherman orthobunyavirus*/*Orthobunyavirus shermanense*;
- Murrumbidgee virus (MURBV), discovered by HTS in mosquitoes (culicid *Anopheles annulipes* Walker, 1856) sampled in Griffith, New South Wales, Australia [70] and Trubanaman virus (TRUV), isolated from mosquitoes (culicid *Anopheles annulipes* Walker, 1856) sampled in North Queensland, Australia [59, 71], were classified into species *Buffalo Creek orthobunyavirus*/*Orthobunyavirus buffaloense*; and
- Salt Ash virus (SASHV), discovered by HTS in moquitoes (culicid *Ochlerotatus vigilax* (Skuse, 1889)) sampled in Port Stephens, New South Wales, Australia [70], was classified into species *Gan Gan orthobunyavirus*/*Orthobunyavirus ganganense*.

Four orthobunyavirus species were abolished and their viruses were moved into established species (TaxoProp 2022.018M.A.Orthobunyavirus_29nsp_abolish4sp):

- *Alajuela orthobunyavirus*: Alajuela virus (ALJV) was moved into species *Gamboa orthobunyavirus/Orthobunyavirus gamboaense*;
- *Benevides orthobunyavirus*: Benevides virus (BVSV=BENV) was moved into species *Acara orthobunyavirus/Orthobunyavirus acaraense*;
- *Bimiti orthobunyavirus*: Bimiti virus (BIMV) was moved into species *Guama orthobunyavirus/Orthobunyavirus guamaense*; and
- *Moju orthobunyavirus*: Moju virus (MOJUV) was moved into species *Catu orthobunyavirus/Orthobunyavirus catuense*.

Eight orthobunyaviruses were reclassified:

- Itaquí virus (ITQV) was moved from *Oriboca orthobunyavirus/Orthobunyavirus oribocaense* to *Caraparu orthobunyavirus/Orthobunyavirus caraparuense*;
- Kaikalur virus (KAIV) was moved from *Shuni orthobunyavirus/Orthobunyavirus shuniense* into *Aino orthobunyavirus/Orthobunyavirus ainoense*;
- Lokern virus (LOKV) and Santa Rosa virus (SARV) were moved from *Bunyamwera orthobunyavirus/Orthobunyavirus bunyamweraense* into *Main Drain orthobunyavirus/Orthobunyavirus kernense*;
- Moriche virus (MORV) was moved from *Acara orthobunyavirus/Orthobunyavirus acaraense* into *Bushbush orthobunyavirus/Orthobunyavirus bushbushense*;
- Murutucú virus (MURV) and Restan virus (RESV) were moved from *Marituba orthobunyavirus/Orthobunyavirus maritubaense* into *Oriboca orthobunyavirus/Orthobunyavirus oribocaense*;
- Playas virus (PLAV) was moved from *Maguari orthobunyavirus/Orthobunyavirus maguariense* to *Cache Valley orthobunyavirus/Orthobunyavirus cacheense*;
- Pueblo Viejo virus (PVV) was moved from *Gamboa orthobunyavirus*/*Orthobunyavirus gamboaense* to *Orthobunyavirus sanjuanense*; and
- Vinces virus (VINV) was moved from *Gamboa orthobunyavirus/Orthobunyavirus caraparuense* to *Madrid orthobunyavirus/Orthobunyavirus madridense*.

#### Family *Phasmaviridae*

The names of all species in the family were changed to binomials as required by the recently amended ICVCN [12, 13, 72] (TaxoProp 2021.031M.A.Phasmaviridae_sprename).

Genus *Feravirus* was expanded by one new species, *Feravirus guaguaense*, for Guagua virus (GUAV) discovered by HTS in mosquitoes (culicid *Mansonia titillans* (Walker, 1848)) sampled in Columbia [unpublished; GenBank #MN661015–7] (TaxoProp 2022.019M.A.Phasmaviridae_3nsp.docx).

Genus *Orthophasmavirus* was expanded by two new species (TaxoProp 2022.019M.A.Phasmaviridae_3nsp.docx):

- *Orthophasmavirus fushunense* for Fǔshùn phasmavirus 1 (FsnPV1) discovered by HTS in planthoppers (delphacid *Sogatella furcifera* (Horváth, 1899)) sampled in China [unpublished; GenBank #MZ209987–9] and
- *Orthophasmavirus sogatellae* for Fǔshùn phasmavirus 2 (FsnPV2) discovered by HTS in planthoppers (delphacid *Laodelphax striatellus* (Fallén, 1826)) sampled in China [unpublished; GenBank #MZ210010–12].

#### Family *Phenuiviridae*

The names of all species in the family (with the exception of *Frijoles phlebovirus*, which unfortunately was overlooked) were changed to binomials as required by the recently amended ICVCN [12–14] (TaxoProp 2021.002M.A.Phenuiviridae_sprenamed).

The family was expanded by one new genus, *Citricivirus*, including one new species, *Citricivirus chongqinense*, for Aphis citricidus bunyavirus (AcBV) discovered by HTS in brown citrus aphids (aphid *Aphis citricidus* (Kirkaldy, 1907)) sampled in Chóngqìng (重庆市), China [73] (TaxoProp 2022.020M.A.Phenuiviridae_2ngen_10nsp_1rensp).

Genus *Bandavirus* was expanded by one new species, *Bandavirus zwieselense*, for Zwiesel bat bandavirus (ZbbV) discovered by HTS in northern bats (vespertilionid *Eptesicus nilssonii* (Keyserling et Blasius, 1839)) sampled in Zwiesel, Bavaria (Bayern), Germany [74] (TaxoProp 2022.020M.A.Phenuiviridae_2ngen_10nsp_1rensp).

Genus *Coguvirus* was expanded by four new species:

- *Coguvirus chinense* for Brassica campestris chinensis coguvirus 1 (BCCoV1) discovered by HTS in bok choy (brassicaceaen *Brassica rapa* L.) sampled in Wǔhàn (武汉市), Húběi Province (湖北省) and Nánjīng (南京市), Jiāngsū Province (江苏省), China (TaxoProp 2022.006M.A.Coguvirus_2nsp2) [75];
- *Coguvirus citrulli* for watermelon crinkle leaf-associated virus 1 (WCLaV1) discovered by HTS in watermelon (cucurbitaceaen *Citrullus lanatus* (Thunberg) Matsum. & Nakai, 1916) sampled in Hénán Province (河南省), China (TaxoProp 2022.005M.A.Coguvirus_2nsp) [72, 76];
- *Coguvirus henanense* for watermelon crinkle leaf-associated virus 2 (WCLaV2) discovered by HTS in watermelon (cucurbitaceaen *Citrullus lanatus* (Thunberg) Matsum. & Nakai, 1916) sampled in Hénán Province (河南省), China (TaxoProp 2022.005M.A.Coguvirus_2nsp) [72, 76]; and
- *Coguvirus yunnanense* for Yúnnán Paris negative-stranded virus (YPNSV) discovered by HTS in bunchflowers (melanthiaceaen *Paris polyphylla* Sm.) sampled in Yúnnán Province (云南省), China (TaxoProp 2022.006M.A.Coguvirus_2nsp2) [77].

Genus *Goukovirus* was expanded by two new species (TaxoProp 2022.020M.A.Phenuiviridae_2ngen_10nsp_1rensp):

- *Goukovirus aphalarae* for Aphalara polygoni bunya-like virus (ApBLV) discovered in HTS data of jumping plant lice (psyllid *Aphalara polygoni* Foerster, 1848) [10] and
- *Goukovirus ceraphri* for Ceraphron bunya-like virus (CerBLV) discovered in HTS data of ceraphronid hymenopterans (*Ceraphron* sp.) [10].

Genus *Horwuvirus* was expanded by one new species, *Horwuvirus solenopsidis*, for Solenopsis invicta virus 14 (SINV14) discovered by HTS in red imported fire ants (formicid *Solenopsis invicta* (Buren, 1972)) collected in Mississippi, USA [78] (TaxoProp 2022.020M.A.Phenuiviridae_2ngen_10nsp_1rensp).

Genus *Laulavirus* was expanded by one new species, *Laulavirus wardellense*, for Wardell virus (WRDV) discovered by HTS in Australian paralysis ticks (ixodid *Ixodes holocyclus* Neumann, 1899) sampled in Wardell, New South Wales, Australia [79] (TaxoProp 2022.020M.A.Phenuiviridae_2ngen_10nsp_1rensp).

Genus *Mobuvirus* was expanded by two new species (TaxoProp 2022.020M.A.Phenuiviridae_2ngen_10nsp_1rensp):

- *Mobuvirus arnae* for Euproctis pseudoconspersa bunyavirus (EPBV) discovered by HTS in tea tussock moths (erebid *Arna pseudoconspersa* Strand, 1914) sampled in Qīngyuǎn (清远市), Guǎngdōng Province (广东省), China [80] and
- *Mobuvirus stephanocirci* for Browner virus (BRWV) discovered by HTS in stephanocircid fleas (*Stephanocircus* sp.) sampled in New South Wales, Australia [81].

Genus *Phlebovirus* was expanded by one new species, *Phlebovirus hediense*, for Hédǐ virus (HEDV) isolated from sand flies (psychodid *Phlebotomus chinensis* Newstead, 1916) sampled in Hédǐ (河底镇), Shānxī Province (山西省), China [82] (TaxoProp 2022.020M.A.Phenuiviridae_2ngen_10nsp_1rensp).

Genus *Uukuvirus* was expanded by one new species, *Uukuvirus toyoense*, for Tōyo virus (TOYOV) discovered by HTS in ticks (ixodid *Haemaphysalis formosensis* Neumann, 1913) sampled in Tōyo (東予市), Ehime Prefecture (愛媛県), Japan [83] (TaxoProp 2022.020M.A.Phenuiviridae_2ngen_10nsp_1rensp).

Species *Melon tenuivirus*/*Tenuivirus cucumeris* was moved into the newly created genus *Mechlorovirus* and renamed *Mechlorovirus cucumeris* (TaxoProp 2022.020M.A.Phenuiviridae_2ngen_10nsp_1rensp).

Culex bunyavirus 1 strain CBunV1/Kern, discovered by HTS in culicid mosquitoes (*Culex* sp.) sampled in the USA [84], was found to be an isolate of the already classified phasivirus Wǔtái mosquito virus (WtMV). Leuven phlebovirus, discovered by HTS in castor bean ticks (ixodid *Ixodes ricinus* (Linnaeus, 1758)) sampled in Belgium [85], was found to be an isolate of the already classified ixovirus Fairhair virus. Sunday Canyon virus, isolated from ticks (argasid *Argas cooleyi* Kohls & Hoogstraal, 1960) sampled in Sunday Canyon, Texas, USA [86], was found to be an isolate of the already classified uukuvirus murre virus (MURV).

#### Family *Tospoviridae*

The names of all species in the family were changed to binomials as required by the recently amended ICVCN [12–14] (TaxoProp 2022.011P.A.Tospoviridae_rename).

## Conclusions

A summary of the current, ICTV-accepted taxonomy of the phylum *Negarnaviricota* is presented in Tables 1–8 and in the form of two posters (Fig. S1).

**Table 1.** ICTV-accepted taxonomy of the order *Muvirales* (*Negarnaviricota: Haploviricotina: Chunqiuviricetes*) as of April 2023

Genus	Species^∗^	Virus (Abbreviation)†
**Family** ***Qinviridae***		
*Yingvirus*	*Yingvirus beihaiense*	Běihǎi sesarmid crab virus 4 (BhSCV4)
	*Yingvirus charybdis*	Wēnzhōu qinvirus-like virus 2 (WzQLV2)
	*Yingvirus hubeiense*	Húběi qinvirus-like virus 1 (HbQLV1)
	*Yingvirus sanxiaense*	Sānxiá qinvirus-like virus 1 (SxQLV1)
	*Yingvirus shaheense*	Shāhé qinvirus-like virus 1 (ShQLV1)
	*Yingvirus wenzhouense*	Wēnzhōu qinvirus-like virus 1 (WzQLV1)
	*Yingvirus wuhanense*	Wǔhàn insect virus 15 (WhIV15)
	*Yingvirus xinzhouense*	Xīnzhōu nematode virus 3 (XzNV3)

Note that viruses are real objects that are assigned to concepts that are called taxa. Species, genera, families, and orders are taxa. As appropriate, cultural significance may be conveyed in virus names.

*Taxon names are always italicized and always begin with a capital letter.

†Virus names are not italicized and are not capitalized, except if the name or a name component is a proper noun. This column lists the virus names with their correct (lack of) capitalization.

**Table 2.** ICTV-accepted taxonomy of the order *Serpentovirales* (*Negarnaviricota: Haploviricotina: Milneviricetes*) as of April 2023

Genus	Species^∗^	Virus (Abbreviation)†
**Family** ***Aspiviridae***		
*Ophiovirus*	*Ophiovirus citri*	citrus psorosis virus (CPsV)
	*Ophiovirus freesiae*	freesia sneak virus (FreSV)
	*Ophiovirus lactucae*	lettuce ring necrosis virus (LRNV)
	*Ophiovirus mirafioriense*	Mirafiori lettuce big-vein virus (MLBVV)
	*Ophiovirus ranunculi*	ranunculus white mottle virus (RWMV)
	*Ophiovirus tulipae*	tulip mild mottle mosaic virus (TMMMV)
	Ophiovirus vaccinii	blueberry mosaic associated virus (BlMaV)

Note that viruses are real objects that are assigned to concepts that are called taxa. Species, genera, families, and orders are taxa. Species, genera, families, and orders are taxa. As appropriate, cultural significance may be conveyed in virus names.

^∗^Taxon names are always italicized and always begin with a capital letter.

†Virus names are not italicized and are not capitalized, except if the name or a name component is a proper noun. This column lists the virus names with their correct (lack of) capitalization.

**Table 3.** ICTV-accepted taxonomy of the order *Jingchuvirales* (*Negarnaviricota: Haploviricotina: Monjiviricetes*) as of April 2023

Genus	Species^∗^	Virus (Abbreviation)†
**Family *Aliusviridae***		
*Obscuruvirus*	*Obscuruvirus quintum*	Atrato chu-like virus 5 (AClV5)
*Ollusvirus*	*Ollusvirus coleopteri*	Húběi coleoptera virus 3 (HbCV3)
	*Ollusvirus culvertonense*	Culverton virus (CvV)
	*Ollusvirus hanchengense*	Hánchéng leafhopper mivirus (HLMV)
	*Ollusvirus hymenopteri*	hymenopteran chu-related virus 123 (HCrV123)
	*Ollusvirus insectii*	hymenopteran chu-related virus 126 (HCrV126)
	*Ollusvirus nomadae*	Nomada lathburiana mononega-like virus (NLmlV)
	*Ollusvirus oropsyllae*	Oropsylla silantiewi mononega-like virus 2 (OSmlV2)
	*Ollusvirus rhagoveliae*	Rhagovelia obesa mononega-like virus (ROmlV)
	*Ollusvirus scaldisense*	Scaldis River bee virus (SRBV)
	*Ollusvirus shayangense*	Shāyáng fly virus 1 (SyFV1)
	*Ollusvirus taiyuanense*	Tàiyuán leafhopper virus (TYLeV)
**Family *Chuviridae***		
*Boscovirus*	*Boscovirus hippoboscidae*	Wǔhàn louse fly virus 7 (WhLFV7)
	*Boscovirus hypoboscidae*	Wǔhàn louse fly virus 6 (WhLFV6)
*Chuvivirus*	*Chuvivirus brunnichi*	Wēnlǐng crustacean virus 14 (WlCV14)
	*Chuvivirus canceris*	Wēnzhōu crab virus 2 (WzCV2)
*Culicidavirus*	*Culicidavirus culicidae*	Wǔhàn mosquito virus 8 (WhMV8)
	*Culicidavirus culicis*	Culex mosquito virus 5 (ClMV5)
	*Culicidavirus imjinense*	Imjin River virus 1 (IjRV1)
	*Culicidavirus quitotaense*	Culex mosquito virus 4 (ClMV4)
*Demapteravirus*	*Demapteravirus dermapteri*	dermapteran chu-related virus 142 (DCrV142)
*Doliuvirus*	*Doliuvirus culisetae*	Mos8Chu0 chuvirus (MoCV)
*Mivirus*	*Mivirus amblyommae*	lone star tick chuvirus 1 (LSTCV1)
	*Mivirus belostomatis*	Belostoma flumineum mononega-like virus (BFmlV)
	*Mivirus boleense*	Bólè tick virus 3 (BTV3)
	*Mivirus changpingense*	Chāngpíng tick virus 2 (CpTV2)
	*Mivirus dermacentoris*	Chāngpíng tick virus 3 (CpTV3)
		Tǎchéng tick virus 5 (TcTV5)
	*Mivirus genovaense*	Genoa virus (GeV)
	*Mivirus karukeraense*	Karukera tick virus (KtV)
	*Mivirus rhipicephali*	brown dog tick mivirus 1 (BDTMV1)
	*Mivirus suffolkense*	Suffolk virus (SFKV)
	*Mivirus wuhanense*	Wǔhàn tick virus 2 (WhTV2)
*Morsusvirus*	*Morsusvirus argatis*	Tǎchéng tick virus 4 (TcTV4)
*Nigecruvirus*	*Nigecruvirus ixodes*	blacklegged tick chuvirus 2 (BlTCV2)
	*Nigecruvirus periplanetae*	Periplaneta americana mononega-like virus (PAmlV)
*Odonatavirus*	*Odonatavirus draconis*	odonatan chu-related virus 137 (OCrV137)
	*Odonatavirus fabricii*	Húběi odonate virus 11 (HbOV11)
	*Odonatavirus odontis*	odonatan chu-related virus 136 (OCrV136)
*Pediavirus*	*Pediavirus cirripedis*	Běihǎi barnacle virus 9 (BhBV9)
*Piscichuvirus*	*Piscichuvirus craterocephali*	hardyhead chuvirus (HhCV)
	*Piscichuvirus franki*	Herr Frank virus 1 (HFrV1)
	*Piscichuvirus lycodontis*	Guǎngdōng red-banded snake chuvirus-like virus (GRSCV)
	*Piscichuvirus sanxiaense*	Sānxiá atyid shrimp virus 4 (SxASC4)
	*Piscichuvirus wenlingense*	Wēnlǐng fish chu-like virus (WFClV)
*Pterovirus*	*Pterovirus chulinense*	hymenopteran chu-related virus OKIAV147 (HCrV147)
*Rochuvirus*	*Rochuvirus chalcocoris*	Chalcocoris rutilans mononega-like virus 2 (CRmlV2)
*Scarabeuvirus*	*Scarabeuvirus blattae*	Wǔchāng cockroach virus 3 (WcLFV3)
	*Scarabeuvirus dentati*	Húběi chuvirus-like virus 3 (HbCLV3)
	*Scarabeuvirus hubeiense*	Húběi chuvirus-like virus 1 (HbCLV1)
	*Scarabeuvirus lampyris*	Lampyris noctiluca chuvirus-like virus 1 (LNClV1)
	*Scarabeuvirus lishiense*	Lĭshì spider virus 1 (LsSV1)
	*Scarabeuvirus supellae*	Supella longipalpa mononega-like virus 2 (SLmlV2)
	*Scarabeuvirus trichopriae*	Trichopria drosophilae mononega-like virus (TDmlV)
*Taceavirus*	*Taceavirus wenlingense*	Wēnlǐng crustacean virus 15 (WlCV15)
*Vapochuvirus*	*Vapochuvirus trialeurodis*	Trialeurodes vaporariorum mononega-like virus 2 (TVmlV2)
**Family *Crepuscuviridae***		
*Aqualaruvirus*	*Aqualaruvirus sialis*	megalopteran chu-related virus 119 (MCrV119)
**Family *Myriaviridae***		
*Myriavirus*	*Myriavirus myriapedis*	Húběi myriapoda virus 8 (HbMV8)
**Family *Natareviridae***		
*Charybdivirus*	*Charybdivirus charybdis*	Wēnzhōu crab virus 3 (WzCV3)

Note that viruses are real objects that are assigned to concepts that are called taxa. Species, genera, families, and orders are taxa. Species, genera, families, and orders are taxa. As appropriate, cultural significance may be conveyed in virus names.

^∗^Taxon names are always italicized and always begin with a capital letter.

†Virus names are not italicized and are not capitalized, except if the name or a name component is a proper noun. This column lists the virus names with their correct (lack of) capitalization. Lists of viruses within a given species are provisional at this point and will likely be amended in the near future.

**Table 4.** ICTV-accepted taxonomy of the order *Mononegavirales* (*Negarnaviricota: Haploviricotina: Monjiviricetes*) as of April 2023

Genus	Species†	Virus (Abbreviation)‡
**Family *Artoviridae***		
*Hexartovirus*	*Hexartovirus cirripedis*	Běihǎi barnacle virus 8 (BhBV8)
	*Hexartovirus lepeophtheiri*	Lepeophtheirus salmonis negative-stranded RNA virus 1 (LsNSRV1)
*Peropuvirus*	*Peropuvirus beihaiense*	Běihǎi rhabdo-like virus 1 (BhRLV1)
	*Peropuvirus dentati*	Húběi rhabdo-like virus 8 (HbRLV8)
	*Peropuvirus hubeiense*	Húběi rhabdo-like virus 6 (HbRLV6)
	*Peropuvirus juli*	Húběi rhabdo-like virus 5 (HbRLV5)
	*Peropuvirus lignarii*	Běihǎi rhabdo-like virus 2 (BhRLV2)
	*Peropuvirus melongenae*	Solanum melongena rhabdo-like virus (SmRLV)
	*Peropuvirus pteromali*	Pteromalus puparum negative-strand RNA virus 1 (PpNSRV1)
**Family *Bornaviridae***		
*Carbovirus*	*Carbovirus queenslandense*	jungle carpet python virus (JCPV)
	*Carbovirus tapeti*	southwest carpet python virus (SWCPV)
*Cultervirus*	*Cultervirus hemicultri*	Wǔhàn sharpbelly bornavirus (WhSBV)
*Orthobornavirus*	*Orthobornavirus alphapsittaciforme*	parrot bornavirus 1 (PaBV1)
		parrot bornavirus 2 (PaBV2)
		parrot bornavirus 3 (PaBV3)
		parrot bornavirus 4 (PaBV4)
		parrot bornavirus 7 (PaBV7)
	*Orthobornavirus avisaquaticae*	aquatic bird bornavirus 1 (ABBV1)
		aquatic bird bornavirus 2 (ABBV2)
	*Orthobornavirus betapsittaciforme*	parrot bornavirus 5 (PaBV5)
	*Orthobornavirus bornaense*	Borna disease virus 1 (BoDV1)
		Borna disease virus 2 (BoDV2)
	*Orthobornavirus caenophidiae*	Caribbean watersnake bornavirus (CWBV)
		Mexican black-tailed rattlesnake bornavirus (MRBV)
	*Orthobornavirus elapsoideae*	Loveridge’s garter snake virus 1 (LGSV1)
	*Orthobornavirus estrildidae*	estrildid finch bornavirus 1 (EsBV1)
	*Orthobornavirus sciuri*	variegated squirrel bornavirus 1 (VSBV1)
	*Orthobornavirus serini*	canary bornavirus 1 (CnBV1)
		canary bornavirus 2 (CnBV2)
		canary bornavirus 3 (CnBV3)
**Family *Filoviridae***		
*Cuevavirus*	*Cuevavirus lloviuense*	Lloviu virus (LLOV)
*Dianlovirus*	*Dianlovirus menglaense*	Měnglà virus (MLAV)
*Oblavirus*	*Oblavirus percae*	Oberland virus (OBLV)
*Orthoebolavirus*	*Orthoebolavirus bombaliense*	Bombali virus (BOMV)
	*Orthoebolavirus bundibugyoense*	Bundibugyo virus (BDBV)
	*Orthoebolavirus restonense*	Reston virus (RESTV)
	*Orthoebolavirus sudanense*	Sudan virus (SUDV)
	*Orthoebolavirus taiense*	Taï Forest virus (TAFV)
	*Orthoebolavirus zairense*	Ebola virus (EBOV)
*Orthomarburgvirus*	*Orthomarburgvirus marburgense*	Marburg virus (MARV)
		Ravn virus (RAVV)
*Striavirus*	*Striavirus antennarii*	Xīlǎng virus (XILV)
Tapjovirus	*Tapjovirus bothropis*	Tapajós virus (TAPV)
*Thamnovirus*	*Thamnovirus kanderense*	Kander virus (KNDV)
	*Thamnovirus percae*	Fiwi virus (FIWIV)
	*Thamnovirus thamnaconi*	Huángjiāo virus (HUJV)
**Family *Lispiviridae***		
*Acridvirus*	*Acridvirus hangzhouense*	Hángzhōu acrida cinerea lispivirus 1 (HzACLV1)
*Aleyavirus*	*Aleyavirus fuyangense*	Bemisia tabaci arlivirus 1 (BtAV1)
*Aleybvirus*	*Aleybvirus fuyangense*	Bemisia tabaci arlivirus 2 (BtAV2)
*Anicalvirus*	*Anicalvirus hangzhouense*	Anisopteromalus calandrae negative-strand RNA virus 2 (AcNSRV2)
*Anidravirus*	*Anidravirus hangzhouense*	Anisopteromalus calandrae negative-strand RNA virus 1 (AcNSRV1)
*Aranavirus*	*Aranavirus guiyangense*	Guìyáng lispivirus 1 (GyLV1)
*Aranbvirus*	*Aranbvirus guiyangense*	Guìyáng lispivirus 2 (GyLV2)
*Arlivirus*	*Arlivirus arachnae*	Lĭshì spider virus 2 (LsSV2)
	*Arlivirus hangzhouense*	Hángzhōu Scotinophara lurida lispivirus 1 (HzSLLV1)
	*Arlivirus ningboense*	Nbu stink bug virus 1 (NbuSBV1)
*Avesvirus*	*Avesvirus sinense*	Arlivirus sp. virus (ALV)
*Copasivirus*	*Copasivirus ivindoense*	isopteran arli-related virus OKIAV103 (IARV103)
	*Copasivirus manlyvaleense*	Jimsystermes virus (JIMV)
*Cybitervirus*	*Cybitervirus niederense*	coleopteran arli-related virus OKIAV107 (CARV107)
*Damravirus*	*Damravirus dentatis*	Húběi odonate virus 10 (HbOV10)
	*Damravirus fushunense*	Fǔshùn Ischnura senegalensis lispivirus 1 (FsISLV1)
*Ganiavirus*	*Ganiavirus tachengense*	Tǎchéng tick virus 6 (TcTV6)
*Hemipvirus*	*Hemipvirus scuti*	Hángzhōu Eysarcoris guttigerus lispivirus 1 (HzEGLV1)
	*Hemipvirus veri*	Hángzhōu Cletus punctiger lispivirus 1 (HzCPLV1)
*Leocovirus*	*Leocovirus coleopteris*	Húběi rhabdo-like virus 3 (HbRLV3)
*Nematovirus*	*Nematovirus wuchangense*	Wǔchāng romanomermis nematode virus 2 (WcRNV2)
*Phelinovirus*	*Phelinovirus aphidis*	hymenopteran arli-related virus OKIAV99 (HARV99)
*Rivapovirus*	*Rivapovirus aleyrodidae*	hemipteran arli-related virus OKIAV94 (HARV94)
*Sanstrivirus*	*Sanstrivirus gerridis*	Sānxiá water strider virus 4 (SxWSV4)
*Stylovirus*	*Stylovirus niederense*	strepsipteran arli-related virus OKIAV104 (SARV104)
*Supelovirus*	*Supelovirus thailandense*	blattodean arli-related virus OKIAV102 (BARV102)
*Synelinevirus*	*Synelinevirus bonnense*	hymenopteran arli-related virus OKIAV98 (HARV98)
	*Synelinevirus paranaense*	Linepithema humile rhabdo-like virus 1 (LhuRLV1)
*Usmuvirus*	*Usmuvirus newyorkense*	Amsterdam virus (AMSV)
*Xenophyvirus*	*Xenophyvirus mathesonense*	hemipteran arli-related virus OKIAV95 (HARV95)
**Family *Mymonaviridae***		
*Auricularimonavirus*	*Auricularimonavirus armillariae*	Armillaria mellea negative strand RNA virus 2 (AmNSRV2)
	*Auricularimonavirus auriculariae*	Auricularia heimuer negative-stranded RNA virus 1 (AhNRSV1)
	*Auricularimonavirus bondarzewiae*	Bondarzewia berkeleyi negative-strand RNA virus 1 (BbNSRV1)
*Botrytimonavirus*	*Botrytimonavirus alphabotrytidis*	Botrytis cinerea negative-stranded RNA virus 5 (BcNSRV5)
		Sclerotinia sclerotiorum negative-stranded RNA virus 10 (SsNSRV10)
	*Botrytimonavirus botrytidis*	Botrytis cinerea negative-stranded RNA virus 7 (BcNSRV7)
		Sclerotinia sclerotiorum negative-stranded RNA virus 11 (SsNSRV11)
	*Botrytimonavirus glycinis*	soybean leaf-associated negative-stranded RNA virus 3 (SLaNSRV3)
	*Botrytimonavirus sclerotiniae*	Sclerotinia sclerotiorum negative-stranded RNA virus 2 (SsNSRV2)
		Sclerotinia sclerotiorum negative-stranded RNA virus 2 A (SsNSRV2A)
		Sclerotinia sclerotiorum negative-stranded RNA virus 4 (SsNSRV4)
		Sclerotinia sclerotiorum negative-stranded RNA virus 4 A (SsNSRV4A)
*Hubramonavirus*	*Hubramonavirus fusarii*	Fusarium oxysporum mymonavirus 1 (FoMyV1)
	*Hubramonavirus golovinomycesae*	Golovinomyces cichoracearum negative-stranded RNA virus (GcNSRV1)
	*Hubramonavirus hubeiense*	Húběi rhabdo-like virus 4 (HbRLV4)
	*Hubramonavirus terrae*	H2BulkLitter1223 virus (H2BLV)
	*Hubramonavirus vitis*	grapevine-associated mononega-like virus 3 (GaMLV3)
*Lentimonavirus*	*Lentimonavirus armillariae*	Armillaria mellea negative-stranded RNA virus 1 (AmNSRV1)
	*Lentimonavirus lentinulae*	Lentinula edodes negative-stranded RNA virus 1 (LeNSRV1)
*Penicillimonavirus*	*Penicillimonavirus alphaerysiphe*	Erysiphe necator associated negative-stranded RNA virus 2 (EnaNSRV2)
	*Penicillimonavirus alphapenicillii*	Penicillium adametzioides negative-stranded RNA virus 1 (PaNsRV1)
	*Penicillimonavirus alphaplasmoparae*	Plasmopara viticola lesion associated mononegaambi virus 1 (PvaMV1)
	*Penicillimonavirus betaerysiphe*	Erysiphe necator associated negative-stranded RNA virus 5 (EnaNSRV5)
	*Penicillimonavirus betapenicillii*	Penicillium glabrum negative-stranded RNA virus 1 (PgRlV1)
	*Penicillimonavirus betaplasmoparae*	Plasmopara viticola lesion associated mononegaambi virus 2 (PvaMV2)
		Plasmopara viticola lesion associated mononegaambi virus 4 (PvaMV4)
	*Penicillimonavirus deltaplasmoparae*	Plasmopara viticola lesion associated mononegaambi virus 5 (PvaMV5)
	*Penicillimonavirus epsilonplasmoparae*	Plasmopara viticola lesion associated mononegaambi virus 6 (PvaMV6)
	*Penicillimonavirus etaplasmoparae*	Plasmopara viticola lesion associated mononegaambi virus 9 (PvaMV9)
	*Penicillimonavirus gammaerysiphe*	Erysiphe necator associated negative-stranded RNA virus 6 (EnaNSRV6)
	*Penicillimonavirus gammaplasmopara*	Plasmopara viticola lesion associated mononegaambi virus 3 (PvaMV3)
	*Penicillimonavirus kilnbarnense*	Kiln Barn virus (KBV)
	*Penicillimonavirus magnaporthe*	Magnaporthe oryzae mononegaambi virus 1 (MoMV1)
	*Penicillimonavirus zetaplasmoparae*	Plasmopara viticola lesion associated mononegaambi virus 7 (PvaMV7)
*Phyllomonavirus*	*Phyllomonavirus gysingense*	Gysinge virus (GYSV)
	*Phyllomonavirus phyllospherae*	soybean leaf-associated negative-stranded RNA virus 4 (SLaNSRV4)
*Plasmopamonavirus*	*Plasmopamonavirus erysiphe*	Erysiphe necator associated negative-stranded RNA virus 23 (EnaNSRV23)
	*Plasmopamonavirus plasmoparae*	Plasmopara viticola lesion associated mononegaambi virus 8 (PvaMV8)
*Rhizomonavirus*	*Rhizomonavirus mali*	apple virus B (APPVB)
*Sclerotimonavirus*	*Sclerotimonavirus alphabotrytidis*	Botrytis cinerea negative-stranded RNA virus 3 (BcNSRV3)
		Sclerotinia sclerotiorum negative-stranded RNA virus 9 (SsNSRV9)
	*Sclerotimonavirus alphaclarireediae*	Sclerotinia homoeocarpa TSA contig 1 (ShTSA1)
		Sclerotinia homoeocarpa TSA contig 2 (ShTSA2)
	*Sclerotimonavirus alphaplasmoparae*	Plasmopara viticola lesion associated mymonavirus 1 (PvaMV1)
	*Sclerotimonavirus alternariae*	Alternaria tenuissima negative-stranded RNA virus 1 (AtNsRV1)
	*Sclerotimonavirus asiafusarii*	Fusarium asiaticum negative-stranded RNA virus 1 (FaNSRV1)
	*Sclerotimonavirus betabotrytidis*	Botrytis cinerea negative-stranded RNA virus 4 (BcNSRV4)
	*Sclerotimonavirus betaclarireediae*	Sclerotinia homoeocarpa TSA contig 3 (ShTSA3)
	*Sclerotimonavirus betaplasmoparae*	Plasmopara viticola lesion associated mononega virus 2 (PvaMV2)
	*Sclerotimonavirus botrytidis*	Botrytis cinerea mymonavirus 1 (BcMyV1)
		Sclerotinia sclerotiorum negative-stranded RNA virus 7 (SsNSRV7)
	*Sclerotimonavirus cryphonectriae*	Cryphonectria parasitica sclerotimonavirus 1 (CpSV1)
	*Sclerotimonavirus fusarii*	Fusarium graminearum negative-stranded RNA virus 1 (FgNSRV1)
		soybean leaf-associated negative-stranded RNA virus 1 (SLaNSRV1)
	*Sclerotimonavirus illinoisense*	soybean leaf-associated negative-stranded RNA virus 2 (SLaNSRV2)
	*Sclerotimonavirus penicillii*	Penicillium cairnsense negative-stranded RNA virus 1 (PcNsRV1)
	*Sclerotimonavirus sclerotiniae*	Sclerotinia sclerotiorum negative-stranded RNA virus 1 (SsNSRV1)
		Sclerotinia sclerotiorum negative-stranded RNA virus 1 A (SsNSRV1-A)
		Sclerotinia sclerotiorum negative-stranded RNA virus 3 (SsNSRV3)
		Sclerotinia sclerotiorum negative-stranded RNA virus 3 A (SsNSRV3-A)
	*Sclerotimonavirus terrae*	H4BulkLitter234 virus (H4BLV)
	*Sclerotimonavirus xinjiangense*	Xīnjiāng mymona-like virus 2 (XjMLV2)
	*Sclerotimonavirus yunnanense*	Yúnnán mymona-like virus 1 (YnMLV1)
**Family *Nyamiviridae***		
*Berhavirus*	*Berhavirus beihaiense*	Běihǎi rhabdo-like virus 4 (BhRLV4)
	*Berhavirus radialis*	Běihǎi rhabdo-like virus 5 (BhRLV5)
	*Berhavirus sipunculi*	Běihǎi rhabdo-like virus 3 (BhRLV3)
*Crustavirus*	*Crustavirus beihaiense*	Běihǎi rhabdo-like virus 6 (BhRLV6)
	*Crustavirus wenlingense*	Wēnlǐng crustacean virus 12 (WlCV12)
	*Crustavirus wenzhouense*	Wēnzhōu crab virus 1 (WzCV1)
*Formivirus*	*Formivirus angliae*	Formica fusca virus 1 (FfusV1)
	*Formivirus chalybii*	hymenopteran orino-related virus OKIAV87 (HORV87)
	*Formivirus finnoniae*	Formica exsecta virus 4 (FeV4)
	*Formivirus gorytis*	hymenopteran orino-related virus OKIAV85 (HORV85)
	*Formivirus pollinis*	Xiāngshān nyami-like virus (XNyV)
	*Formivirus solenopsi*	Solenopsis invicta virus 15 (SoINV15)
*Nyavirus*	*Nyavirus argatis*	Sekira virus (SEKRV)
	*Nyavirus midwayense*	Midway virus (MIDWV)
	*Nyavirus nyamaniniense*	Nyamanini virus (NYMV)
	*Nyavirus sanjacintoense*	San Jacinto virus (SJCV)
	*Nyavirus sierranevadaense*	Sierra Nevada virus (SNVV)
	*Nyavirus somateriae*	Jeremy Point nyavirus (JPNV)
*Orinovirus*	*Orinovirus pasiphilae*	Orinoco virus (ONCV)
	*Orinovirus sanyae*	Sanya nyamivirus 1 (SaNyV1)
*Socyvirus*	*Socyvirus heteroderae*	soybean cyst nematode virus 1 (SbCNV1)
*Tapwovirus*	*Tapwovirus cesti*	Wēnzhōu tapeworm virus 1 (WzTWV1)
**Family *Paramyxoviridae***		
Subfamily *Avulavirinae*		
*Metaavulavirus*	*Metaavulavirus delawarense*	avian paramyxovirus 8 (APMV8)
	*Metaavulavirus falklandense*	avian paramyxovirus 10 (APMV10)
	*Metaavulavirus galliense*	avian paramyxovirus 11 (APMV11)
	*Metaavulavirus hongkongense*	avian paramyxovirus 6 (APMV6)
	*Metaavulavirus japanense*	avian paramyxovirus 14 (APMV14)
	*Metaavulavirus kazakhstanense*	avian paramyxovirus 20 (APMV20)
	*Metaavulavirus kunitachiense*	avian paramyxovirus 5 (APMV5)
	*Metaavulavirus peixense*	avian paramyxovirus 15 (APMV15)
	*Metaavulavirus taiwanense*	avian paramyxovirus 22 (APMV22)
	*Metaavulavirus tennessense*	avian paramyxovirus 7 (APMV7)
	*Metaavulavirus yucaipaense*	avian paramyxovirus 2 (APMV2)
*Orthoavulavirus*	*Orthoavulavirus borisense*	Antarctic penguin virus A (APVA)
	*Orthoavulavirus italiense*	avian paramyxovirus 12 (APMV12)
	*Orthoavulavirus japanense*	avian paramyxovirus 13 (APMV13)
	*Orthoavulavirus javaense*	avian paramyxovirus 1 (APMV1)^1^
	*Orthoavulavirus kopaiticense*	Antarctic penguin virus B (APVB)
	*Orthoavulavirus koreaense*	avian paramyxovirus 21 (APMV21)
	*Orthoavulavirus newyorkense*	avian paramyxovirus 9 (APMV9)
	*Orthoavulavirus oneillense*	Antarctic penguin virus C (APVC)
	*Orthoavulavirus upoense*	avian paramyxovirus 16 (APMV16)
*Paraavulavirus*	*Paraavulavirus hongkongense*	avian paramyxovirus 4 (APMV4)
	*Paraavulavirus wisconsinense*	avian paramyxovirus 3 (APMV3)
**Subfamily *Metaparamyxovirinae***		
*Synodonvirus*	*Synodonvirus synodi*	Wēnlǐng triplecross lizardfish paramyxovirus (WTLPV)
**Subfamily *Orthoparamyxovirinae***		
*Aquaparamyxovirus*	*Aquaparamyxovirus oregonense*	Pacific salmon paramyxovirus (PSPV)
	*Aquaparamyxovirus salmonis*	Atlantic salmon paramyxovirus (AsaPV)
*Ferlavirus*	*Ferlavirus reptilis*	fer-de-lance virus (FDLV)
*Henipavirus*	*Henipavirus cedarense*	Cedar virus (CedV)
	*Henipavirus ghanaense*	Ghana virus (GhV)
	*Henipavirus hendraense*	Hendra virus (HeV)
	*Henipavirus mojiangense*	Mòjiāng virus (MojV)
	*Henipavirus nipahense*	Nipah virus (NiV)
*Jeilongvirus*	*Jeilongvirus anhuiense*	Miniopterus schreibersii paramyxovirus (MisPV)
	*Jeilongvirus apodemi*	rodent paramyxovirus (RoPV)
	*Jeilongvirus beilongi*	Beilong virus (BeiV)
	*Jeilongvirus comorosense*	bat paramyxovirus 16 797 (BatPV1)
	*Jeilongvirus erinacei*	belerina virus (BeV)
	*Jeilongvirus felis*	feline paramyxovirus (FPaV)
	*Jeilongvirus lophuromysis*	Mount Mabu Lophuromys virus 1 (MMLV1)
	*Jeilongvirus mabuense*	Mount Mabu Lophuromys virus 2 (MMLV2)
	*Jeilongvirus madagascarense*	bat paramyxovirus 17 770 (BatPV2)
	*Jeilongvirus miniopteri*	Shaan virus (ShaV)
	*Jeilongvirus murinae*	bat paramyxovirus (BatPV3)
	*Jeilongvirus myodesis*	Pohorje Myodes paramyxovirus 1 (PMPV1)
	*Jeilongvirus queenslandense*	J virus (JV)
	*Jeilongvirus rungweense*	ruloma virus (RulV)
	*Jeilongvirus tailamense*	Tailam virus (TaiV)
*Morbillivirus*	*Morbillivirus canis*	canine distemper virus (CDV)
	*Morbillivirus caprinae*	peste-des-petits-ruminants virus (PPRV)
	*Morbillivirus ceti*	cetacean morbillivirus (CeMV)
	*Morbillivirus felis*	feline morbillivirus (FeMV)
	*Morbillivirus hominis*	measles virus (MeV)
	*Morbillivirus pecoris*	rinderpest virus (RPV)
	*Morbillivirus phocae*	phocine distemper virus (PDV)
*Narmovirus*	*Narmovirus mossmanense*	Mossman virus (MossV)
	*Narmovirus myodesis*	bank vole virus 1 (BaV1)
	*Narmovirus narivaense*	Nariva virus (NarV)
	*Narmovirus tupaiae*	Tupaia paramyxovirus (TupV)
*Respirovirus*	*Respirovirus bovis*	bovine parainfluenza virus 3 (BPIV3)
	*Respirovirus caprae*	caprine parainfluenzavirus 3 (CPIV3)
	*Respirovirus laryngotracheitidis*	human parainfluenza virus 1 (HPIV1)
	*Respirovirus muris*	Sendai virus (SeV)
	*Respirovirus pneumoniae*	human parainfluenza virus 3 (HPIV3)
	*Respirovirus ratufae*	giant squirrel virus (GSqV)
	*Respirovirus suis*	porcine parainfluenza virus 1 (PPIV1)
*Salemvirus*	*Salemvirus salemense*	Salem virus (SalV)
**Subfamily *Rubulavirinae***		
*Orthorubulavirus*	*Orthorubulavirus alstonvillense*	Alston virus (AlsV)
	*Orthorubulavirus hominis*	human parainfluenza virus 4 a (HPIV4a)
		human parainfluenza virus 4b (HPIV4b)
	*Orthorubulavirus laryngotracheitidis*	human parainfluenza virus 2 (HPIV2)
	*Orthorubulavirus mammalis*	parainfluenza virus 5 (PIV5)
	*Orthorubulavirus mapueraense*	Mapuera virus (MapV)
	*Orthorubulavirus parotitidis*	mumps virus (MuV)
	*Orthorubulavirus simiae*	simian virus 41 (SV41)
	*Orthorubulavirus suis*	La Piedad Michoacán Mexico virus (LPMV)
*Pararubulavirus*	*Pararubulavirus accraense*	Achimota virus 2 (AchPV2)
	*Pararubulavirus achimotaense*	Achimota virus 1 (AchPV1)
	*Pararubulavirus cantonense*	Tuhoko virus 2 (ThkPV2)
	*Pararubulavirus guangdongense*	Tuhoko virus 1 (ThkPV1)
	*Pararubulavirus herveyense*	Hervey virus (HerV)
	*Pararubulavirus hongkongi*	Tuhoko virus 3 (ThkPV3)
	*Pararubulavirus menangleense*	Menangle virus (MenPV)
	*Pararubulavirus sosugaense*	Sosuga virus (SOSV)
	*Pararubulavirus teviotense*	Teviot virus (TevPV)
	*Pararubulavirus tiomanense*	Tioman virus (TioPV)
**Unassigned (to subfamilies)**		
*Cynoglossusvirus*	*Cynoglossusvirus cynoglossi*	Wēnlǐng tonguesole paramyxovirus (WTSPV)
*Hoplichthysvirus*	*Hoplichthysvirus hoplichthysis*	Wēnlǐng hoplichthys paramyxovirus (WHPV)
*Scoliodonvirus*	*Scoliodonvirus scoliodontis*	Wēnzhōu pacific spadenose shark paramyxovirus (WPSSPV)
F**amily *Pneumoviridae***		
*Metapneumovirus*	*Metapneumovirus avis*	avian metapneumovirus (AMPV)
	*Metapneumovirus hominis*	human metapneumovirus (HMPV)
*Orthopneumovirus*	*Orthopneumovirus bovis*	bovine respiratory syncytial virus (BRSV)
	*Orthopneumovirus hominis*	human respiratory syncytial virus (HRSV)
	*Orthopneumovirus muris*	murine pneumonia virus (MPV)
**Family *Rhabdoviridae***		
**Subfamily *Alpharhabdovirinae***		
*Almendravirus*	*Almendravirus almendras*	Puerto Almendras virus (PTAMV)
	*Almendravirus arboretum*	Arboretum virus (ABTV)
	*Almendravirus balsa*	Balsa virus (BALV)
	*Almendravirus chico*	Rio Chico virus (RCHV)
	*Almendravirus cootbay*	Coot Bay virus (CBV)
	*Almendravirus menghai*	Menghai virus (MRV)
	*Almendravirus xianshan*	Xiangshan rhabdo-like virus 1 (XsRLV1)
*Alphanemrhavirus*	*Alphanemrhavirus bangkok*	Rattus tanezumi rhabdovirus 1 (RtaRV1)
	*Alphanemrhavirus sodak*	Sodak rhabdovirus 1 (SDRV1)
	*Alphanemrhavirus xingshan*	Xingshan nematode virus 4 (XsNV4)
	*Alphanemrhavirus xinzhou*	Xinzhou nematode virus 4 (XzNV4)
*Alphapaprhavirus*	*Alphapaprhavirus hubei*	Hubei lepidoptera virus 2 (HbLV2)
	*Alphapaprhavirus pararge*	Pararge aegeria rhabdovirus (PAeRV)
*Alpharicinrhavirus*	*Alpharicinrhavirus blanchseco*	Blanchseco virus (BCOV)
	*Alpharicinrhavirus bole*	Bole tick virus 2 (BlTV2)
	*Alpharicinrhavirus hubei*	Hubei tick rhabdovirus 1 (HbTRV1)
	*Alpharicinrhavirus wuhan*	Wuhan tick virus 1 (WhTV1)
*Amplylivirus*	*Amplylivirus cinereus*	frog lyssa-like virus 1 (FLLV1)
*Arurhavirus*	*Arurhavirus aruac*	Aruac virus (ARUV)
	*Arurhavirus inhangapi*	Inhangapi virus (INHV)
	*Arurhavirus santabarbara*	Santa Barbara virus (SBAV)
	*Arurhavirus xiburema*	Xiburema virus (XIBV)
*Barhavirus*	*Barhavirus bahia*	Bahia Grande virus (BGV)
		Harlingen virus (HARV)
	*Barhavirus muir*	Muir Springs virus (MSV)
*Caligrhavirus*	*Caligrhavirus caligus*	Caligus rogercresseyi rhabdovirus (CRogRV)
	*Caligrhavirus lepeophtheirus*	Lepeophtheirus salmonis rhabdovirus 127 (LSalRV127)
	*Caligrhavirus salmonlouse*	Lepeophtheirus salmonis rhabdovirus 9 (LSalRV9)
*Cetarhavirus*	*Cetarhavirus laganorhynchus*	dolphin rhabdovirus (DRV)
	*Cetarhavirus phocoena*	harbour porpoise rhabdovirus (HPRV)
*Curiovirus*	*Curiovirus curionopolis*	Curionopolis virus (CURV)
	*Curiovirus iriri*	Iriri virus (IRIRV)
	*Curiovirus itacaiunas*	Itacaiunas virus (ITAV)
	*Curiovirus rochambeau*	Rochambeau virus (RBUV)
*Ephemerovirus*	*Ephemerovirus adelaide*	Adelaide River virus (ARV)
	*Ephemerovirus berrimah*	Berrimah virus (BRMV)
	*Ephemerovirus febris*	bovine ephemeral fever virus (BEFV)
	*Ephemerovirus guangdong*	porcine ephemerovirus 2 (PoEV2)
	*Ephemerovirus hayes*	Hayes Yard virus (HYV)
	*Ephemerovirus henan*	porcine ephemerovirus 1 (PoEV1)
	*Ephemerovirus kent*	New Kent County virus (NKCV)
	*Ephemerovirus kimberley*	Kimberley virus (KIMV)
		Malakal virus (MALV)
	*Ephemerovirus koolpinyah*	Koolpinyah virus (KOOLV)
	*Ephemerovirus kotonkan*	kotonkan virus (KOTV)
	*Ephemerovirus obodhiang*	Obodhiang virus (OBOV)
	*Ephemerovirus puchong*	Puchong virus (PUCV)
	*Ephemerovirus yata*	Yata virus (YATV)
*Hapavirus*	*Hapavirus bangoran*	Bangoran virus (BGNV)
	*Hapavirus flanders*	Flanders virus (FLAV)
	*Hapavirus graylodge*	Grey Lodge virus (GLOV)
	*Hapavirus hartpark*	Hart Park virus (HPV)
	*Hapavirus holmes*	Holmes Jungle virus (HOJV)
	*Hapavirus joinjakaka*	Joinjakaka virus (JOIV)
	*Hapavirus kamese*	Kamese virus (KAMV)
	*Hapavirus lajoya*	La Joya virus (LJV)
	*Hapavirus landjia*	Landjia virus (LANV=LJAV)
	*Hapavirus manitoba*	Manitoba virus (MANV=MNTBV)
	*Hapavirus marco*	Marco virus (MCOV)
	*Hapavirus mosqueiro*	Mosqueiro virus (MQOV)
	*Hapavirus mossuril*	Mossuril virus (MOSV)
	*Hapavirus ngaingan*	Ngaingan virus (NGAV)
	*Hapavirus ord*	Ord River virus (ORV)
	*Hapavirus parry*	Parry Creek virus (PCV)
	*Hapavirus porton*	Porton virus (PORV)
	*Hapavirus wongabel*	Wongabel virus (WONV)
*Ledantevirus*	*Ledantevirus barur*	Barur virus (BARV)
	*Ledantevirus bughendera*	Bughendera virus (BUGV)
	*Ledantevirus elgon*	Mount Elgon bat virus (MEBV)
	*Ledantevirus fikirini*	Fikirini virus (FKRV)
	*Ledantevirus fukuoka*	Fukuoka virus (FUKV)
	*Ledantevirus kanyawara*	Kanyawara virus (KYAV)
	*Ledantevirus kern*	Kern Canyon virus (KCV)
	*Ledantevirus keuraliba*	Keuraliba virus (KEUV)
	*Ledantevirus kolente*	Kolente virus (KOLEV)
	*Ledantevirus kumasi*	Kumasi rhabdovirus (KRV)
	*Ledantevirus ledantec*	Le Dantec virus (LDV)
	*Ledantevirus longquan*	Longquan Niviventer coninga ledantevirus 1 (LNcoLV1)
	*Ledantevirus nishimuro*	Nishimuro virus (NISV)
	*Ledantevirus nkolbisson*	Nkolbisson virus (NKOV)
	*Ledantevirus oita*	Oita virus (OITAV)
	*Ledantevirus taiyi*	Taiyi bat virus (TYBV)
	*Ledantevirus vaprio*	Vaprio virus (VAPV)
	*Ledantevirus wenzhou*	Wenzhou Rhinolophus pusillus ledantevirus 1 (WRpuLV1)
	*Ledantevirus wuhan*	Wuhan louse fly virus 5 (WLFV5)
	*Ledantevirus yongjia*	Yongjia tick virus 2 (YTV2)
*Lostrhavirus*	*Lostrhavirus hyalomma*	Xinjiang tick rhabdovirus (XjTRV)
	Lostrhavirus lonestar	lone star tick rhabdovirus (LITRV)
Lyssavirus	Lyssavirus aravan	Aravan virus (ARAV)
	*Lyssavirus australis*	Australian bat lyssavirus (ABLV)
	*Lyssavirus bokeloh*	Bokeloh bat lyssavirus (BBLV)
	*Lyssavirus caucasicus*	West Caucasian bat virus (WCBV)
	*Lyssavirus duvenhage*	Duvenhage virus (DUVV)
	*Lyssavirus formosa*	Taiwan bat lyssavirus (TWBLV)
	*Lyssavirus gannoruwa*	Gannoruwa bat lyssavirus (GBLV)
	*Lyssavirus hamburg*	European bat lyssavirus 1 (EBLV1)
	*Lyssavirus helsinki*	European bat lyssavirus 2 (EBLV2)
	*Lyssavirus ikoma*	Ikoma lyssavirus (IKOV)
	*Lyssavirus irkut*	Irkut virus (IRKV)
	*Lyssavirus khujand*	Khujand virus (KHUV)
	*Lyssavirus lagos*	Lagos bat virus (LBV)
	*Lyssavirus lleida*	Lleida bat lyssavirus (LLEBV)
	*Lyssavirus mokola*	Mokola virus (MOKV)
	*Lyssavirus rabies*	rabies virus (RABV)
	*Lyssavirus shimoni*	Shimoni bat virus (SHIBV)
*Merhavirus*	*Merhavirus merida*	Merida virus (MERDV)
	*Merhavirus tritaeniorhynchus*	Culex tritaeniorhynchus rhabdovirus (CTRV)
*Mousrhavirus*	*Mousrhavirus moussa*	Moussa virus (MOUV)
*Ohlsrhavirus*	*Ohlsrhavirus angeles*	Culex rhabdo-like virus Los Angeles (CRLVLA)
	*Ohlsrhavirus culex*	Culex rhabdo-like virus (CRLV)
	*Ohlsrhavirus lobeira*	Lobeira virus (LOBV)
	*Ohlsrhavirus northcreek*	North Creek virus (NORCV)
	*Ohlsrhavirus ohlsdorf*	Ohlsdorf virus (OHLDV)
	*Ohlsrhavirus pseudovishnui*	Culex pseudovishnui rhabdo-like virus (CpRLV)
	*Ohlsrhavirus riverside*	Riverside virus (RISV)
	*Ohlsrhavirus tongilchon*	Tongilchon virus 1 (TCHV1)
*Perhabdovirus*	*Perhabdovirus anguilla*	eel virus American (EVA)
		eel virus European X (EVEX)
	*Perhabdovirus leman*	Leman virus (LeRV)
	*Perhabdovirus perca*	perch rhabdovirus (PRV)
	*Perhabdovirus trutta*	lake trout rhabdovirus (LTRV)
*Replylivirus*	*Replylivirus allogus*	anole lyssa-like virus 1 (ALLV1)
*Sawgrhavirus*	*Sawgrhavirus connecticut*	Connecticut virus (CNTV)
	*Sawgrhavirus longisland*	Long Island tick rhabdovirus (LITRV)
	*Sawgrhavirus minto*	New Minto virus (NMV)
	*Sawgrhavirus sawgrass*	Sawgrass virus (SAWV)
*Scophrhavirus*	*Scophrhavirus chanodychthys*	Wuhan redfin culter dimarhabdovirus (WhRFCRV)
	*Scophrhavirus maximus*	Scophthalmus maximus rhabdovirus (SMRV)
*Sigmavirus*	*Sigmavirus affinis*	Drosophila affinis sigmavirus (DAffSV)
	*Sigmavirus ananassae*	Drosophila ananassae sigmavirus (DAnaSV)
	*Sigmavirus capitata*	Ceratitis capitata sigmavirus (CCapSV)
	*Sigmavirus domestica*	Wuhan fly virus 2 (WhFV2)
	*Sigmavirus hippoboscid*	Wuhan louse fly virus 9 (WhLFV9)
	*Sigmavirus hubei*	Hubei diptera virus 9 (HbDV9)
	*Sigmavirus immigrans*	Drosophila immigrans sigmavirus (DImmSV)
	*Sigmavirus lousefly*	Wuhan louse fly virus 10 (WhLFV10)
	*Sigmavirus melanogaster*	Drosophila melanogaster sigmavirus (DMelSV)
	*Sigmavirus muscina*	Muscina stabulans sigmavirus (MStaSV)
	*Sigmavirus myga*	Hubei diptera virus 10 (HbDV10)
	*Sigmavirus obscura*	Drosophila obscura sigmavirus (DObsSV)
	*Sigmavirus shayang*	Shayang fly virus 2 (SyFV2)
	*Sigmavirus sichuan*	Apis rhabdovirus 3 (ApRV3)
	*Sigmavirus sturtevanti*	Drosophila sturtevanti sigmavirus (DStuSV)
	*Sigmavirus tristis*	Drosophila tristis sigmavirus (DTriSV)
	*Sigmavirus tuva*	Aksy-Durug Melophagus sigmavirus (ADMSV)
	*Sigmavirus wuhan*	Wuhan house fly virus 1 (WhHFV1)
	*Sigmavirus ying*	Hubei dimarhabdovirus 1 (HbDRV1)
	*Sigmavirus yushu*	Yushu rhabdovirus (YsRV)
*Siniperhavirus*	*Siniperhavirus chuatsi*	Chinese rice-field eel rhabdovirus (CrERV)
		hybrid snakehead rhabdovirus (HSHRV)
		Micropterus salmoides rhabdovirus (MSRV)
		Siniperca chuatsi rhabdovirus (SCRV)
	*Siniperhavirus zoarces*	eelpout rhabdovirus (EPRV)
*Sprivivirus*	*Sprivivirus cyprinus*	spring viremia of carp virus (SVCV)
	*Sprivivirus esox*	grass carp rhabdovirus (GrCRV)
		pike fry rhabdovirus (PFRV)
		tench rhabdovirus (TenRV)
*Sripuvirus*	*Sripuvirus almpiwar*	Almpiwar virus (ALMV)
	*Sripuvirus chaco*	Chaco virus (CHOV)
	*Sripuvirus charleville*	Charleville virus (CHVV)
	*Sripuvirus cuiaba*	Cuiaba virus (CUIV)
	*Sripuvirus hainan*	Hainan black-spectacled toad rhabdovirus (HnBSTRV)
	*Sripuvirus madureira*	Sena Madureira virus (SMV)
	*Sripuvirus niakha*	Niakha virus (NIAV)
	*Sripuvirus sripur*	Sripur virus (SRIV)
*Sunrhavirus*	*Sunrhavirus alexandria*	Burg el Arab virus (BEAV)
	*Sunrhavirus bimbo*	Bimbo virus (BBOV)
	*Sunrhavirus boteke*	Boteke virus (BOTV)
	*Sunrhavirus dillard*	Dillard’s Draw virus (DDRV)
	*Sunrhavirus garba*	Garba virus (GARV)
	*Sunrhavirus harrison*	Harrison Dam virus (HARDV)
	*Sunrhavirus kolongo*	Kolongo virus (KOLV)
	*Sunrhavirus kwatta*	Kwatta virus (KWAV)
	*Sunrhavirus matariya*	Matariya virus (MTYV)
	*Sunrhavirus nasoule*	Nasoule virus (NASV)
	*Sunrhavirus oakvale*	Oak Vale virus (OVV)
	*Sunrhavirus ouango*	Ouango virus (OUAV)
	*Sunrhavirus sandjimba*	Sandjimba virus (SJAV)
	*Sunrhavirus sunguru*	Sunguru virus (SUNV)
	*Sunrhavirus walkabout*	Walkabout Creek virus (WACV)
*Thriprhavirus*	*Thriprhavirus intonsa*	Hangzhou Frankliniella intonsa rhabdovirus 1 (HFinRV1)
	*Thriprhavirus tabaci*	Thrips tabaci associated dimarhabdovirus 1 (TtaDRV1)
*Tibrovirus*	*Tibrovirus alphaekpoma*	Ekpoma virus 1 (EKV1)
	*Tibrovirus beatrice*	Beatrice Hill virus (BHV)
	*Tibrovirus betaekpoma*	Ekpoma virus 2 (EKV2)
	*Tibrovirus coastal*	Coastal Plains virus (CPV)
	*Tibrovirus congo*	Bas-Congo virus (BASV)
	*Tibrovirus mundri*	Mundri virus (MUNV)
	*Tibrovirus sweetwater*	Sweetwater Branch virus (SWBV)
	*Tibrovirus tibrogargan*	Bivens Arm virus (BAV)
		Tibrogargan virus (TIBV)
*Tupavirus*	*Tupavirus durham*	Durham virus (DURV)
	*Tupavirus klamath*	Klamath virus (KLAV)
	*Tupavirus laniger*	Wenzhou Myotis laniger tupavirus 1 (WMlaTV1)
	*Tupavirus pearsonii*	Wufeng Rhinolophus pearsonii tupavirus 1 (WRpeTV1)
	*Tupavirus tupaia*	tupaia rhabdovirus (TUPV)
*Vesiculovirus*	*Vesiculovirus alagoas*	vesicular stomatitis Alagoas virus (VSAV)
	*Vesiculovirus bogdanovac*	Yug Bogdanovac virus (YBV)
	*Vesiculovirus carajas*	Carajás virus (CJSV)
	*Vesiculovirus chandipura*	Chandipura virus (CHPV)
	*Vesiculovirus cocal*	Cocal virus (COCV)
	*Vesiculovirus eptesicus*	American bat vesiculovirus (ABVV)
	*Vesiculovirus indiana*	vesicular stomatitis Indiana virus (VSIV)
	*Vesiculovirus isfahan*	Isfahan virus (ISFV)
	*Vesiculovirus jurona*	Jurona virus (JURV)
	*Vesiculovirus malpais*	Malpais Spring virus (MSPV)
	*Vesiculovirus maraba*	Maraba virus (MARAV)
	*Vesiculovirus mediterranean*	Mediterranean bat virus (MBV)
	*Vesiculovirus mejal*	Mejal virus (MEJV)
	*Vesiculovirus morreton*	Morreton virus (MORV)
	*Vesiculovirus newjersey*	vesicular stomatitis New Jersey virus (VSNJV)
	*Vesiculovirus perinet*	Perinet virus (PERV)
	*Vesiculovirus piry*	Piry virus (PIRYV)
	*Vesiculovirus radi*	Radi virus (RADV)
	*Vesiculovirus rhinolophus*	Jinghong bat virus (JhBV)
		Qiongzhong bat virus (QZBV)
	*Vesiculovirus wufeng*	Wufeng Myotis altarium vesiculovirus 1 (WMalVV1)
	*Vesiculovirus yinshui*	Yinshui bat virus (YSBV)
*Zarhavirus*	*Zarhavirus zahedan*	Zahedan rhabdovirus (ZARV)
**Subfamily *Betarhabdovirinae***		
*Alphanucleorhabdovirus*	*Alphanucleorhabdovirus agavis*	Agave tequilana virus 1 (ATV1)
	*Alphanucleorhabdovirus artemisiae*	Artemisia capillaris nucleorhabdovirus 1 (ArtCaNV1)
	*Alphanucleorhabdovirus colocasiae*	taro vein chlorosis virus (TaVCV)
	*Alphanucleorhabdovirus constrictae*	constricta yellow dwarf virus (CYDV)
	*Alphanucleorhabdovirus joa*	joa yellow blotch associated virus (JYBaV)
	*Alphanucleorhabdovirus maydis*	maize mosaic virus (MMV)
	*Alphanucleorhabdovirus melongenae*	eggplant mottled dwarf virus (EMDV)
	*Alphanucleorhabdovirus morogoromaydis*	Morogoro maize-associated virus (MMaV)
	*Alphanucleorhabdovirus oryzae*	rice transitory yellowing virus (RTYV)
		rice yellow stunt virus (RYSV)
	*Alphanucleorhabdovirus physostegiae*	Physostegia chlorotic mottle virus (PhCMoV)
	*Alphanucleorhabdovirus pruni*	peach virus 1 (PeV1)
	*Alphanucleorhabdovirus tritici*	wheat yellow striate virus (WYSV)
	*Alphanucleorhabdovirus tuberosum*	potato yellow dwarf virus (PYDV)
	*Alphanucleorhabdovirus zeairanense*	maize Iranian mosaic virus (MIMV)
*Betanucleorhabdovirus*	*Betanucleorhabdovirus asclepiadis*	Asclepias syriaca virus 2 (AscSyV2)
	*Betanucleorhabdovirus bacopae*	Bacopa monnieri virus 2 (BmV2)
	*Betanucleorhabdovirus cardamomi*	cardamom vein clearing virus (CdVCV)
	*Betanucleorhabdovirus cnidii*	Cnidium virus 1 (CnV1)
	*Betanucleorhabdovirus daturae*	Datura yellow vein virus (DYVV)
	*Betanucleorhabdovirus loti*	birds-foot trefoil-associated virus (BFTV)
	*Betanucleorhabdovirus mali*	apple rootstock virus A (ApRVA)
	*Betanucleorhabdovirus medicagonis*	alfalfa-associated nucleorhabdovirus (AaNV)
	*Betanucleorhabdovirus plectranthi*	Plectranthus aromaticus virus 1 (PleArV1)
	*Betanucleorhabdovirus retesonchi*	Sonchus yellow net virus (SYNV)
	*Betanucleorhabdovirus rhododendri*	Rhododendon delavayi virus 1 (RhoDeV1)
	*Betanucleorhabdovirus ribes*	blackcurrant-associated rhabdovirus (BCaRV)
	*Betanucleorhabdovirus venasonchi*	sowthistle yellow vein virus (SYVV)
	*Betanucleorhabdovirus zanthoxyli*	Zhuye pepper nucleorhabdovirus (ZPNRV)
*Cytorhabdovirus*	*Cytorhabdovirus actinidiae*	Actinidia virus D (AcVD)
	*Cytorhabdovirus alphatrifolii*	Trifolium pratense virus A (TpVA)
	*Cytorhabdovirus alphawuhaninsectum*	Wuhan insect virus 4 (WuIV4)
	*Cytorhabdovirus anthurii*	Anthurium amnicola virus 1 (AntAmV1)
	*Cytorhabdovirus asclepiadis*	Asclepias syriaca virus 1 (AscSyV1)
	*Cytorhabdovirus bacopae*	Bacopa monnieri virus 1 (BmV1)
	*Cytorhabdovirus bemisiae*	Bemisia tabaci-associated virus 1 (BeTaV1)
	*Cytorhabdovirus betatrifolii*	Trifolium pratense virus B (TpVB)
	*Cytorhabdovirus betawuhaninsectum*	Wuhan insect virus 5 (WuIV5)
	*Cytorhabdovirus brassicae*	broccoli necrotic yellows virus (BNYV)
	*Cytorhabdovirus brassicicolae*	cabbage cytorhabdovirus 1 (CCyV1)
	*Cytorhabdovirus broussonetiae*	paper mulberry mosaic associated virus (PMuMaV)
	*Cytorhabdovirus caricae*	papaya virus E (PpVE)
	*Cytorhabdovirus chrysanthemi*	chrysanthemum yellow dwarf associated virus (CYDaV)
	*Cytorhabdovirus colocasiae*	Colocasia bobone disease-associated virus (CBDaV)
	*Cytorhabdovirus cucurbitae*	cucurbit cytorhabdovirus 1 (CuCV1)
	*Cytorhabdovirus festucae*	Festuca leaf streak virus (FLSV)
	*Cytorhabdovirus flaviyerbamate*	yerba mate chlorosis-associated virus (YmCaV)
	*Cytorhabdovirus fragariae*	strawberry-associated virus 1 (SaV1)
		strawberry virus 1 (StrV1)
	*Cytorhabdovirus fragariarugosus*	strawberry crinkle virus (SCV)
	*Cytorhabdovirus gammawuhaninsectum*	Wuhan insect virus 6 (WuIV6)
	*Cytorhabdovirus glehniae*	Glehnia littoralis virus 1 (GlLV1)
	*Cytorhabdovirus gramineae*	northern cereal mosaic virus (NCMV)
	*Cytorhabdovirus hordei*	barley yellow striate mosaic virus (BYSMV)
	*Cytorhabdovirus kenyatuberosum*	Kenyan potato cytorhabdovirus (KePCyV)
	*Cytorhabdovirus lactucamaculante*	lettuce yellow mottle virus (LYMoV)
	*Cytorhabdovirus lactucanecante*	lettuce necrotic yellows virus (LNYV)
	*Cytorhabdovirus lycopersici*	tomato yellow mottle-associated virus (TYMaV)
	*Cytorhabdovirus maydis*	maize-associated cytorhabdovirus (MaCV)
	*Cytorhabdovirus maysflavostriatis*	maize yellow striate virus (MYSV)
	*Cytorhabdovirus medicagonis*	alfalfa dwarf virus (ADV)
	*Cytorhabdovirus nymphaeae*	Nymphaea alba virus 1 (NymAV1)
	*Cytorhabdovirus orchidaceae*	Gymnadenia densiflora virus 1 (GymDenV1)
	*Cytorhabdovirus oryzae*	rice stripe mosaic virus (RSMV)
	*Cytorhabdovirus persimmon*	persimmon virus A (PeVA)
	*Cytorhabdovirus rosae*	rose virus R (RVR)
	*Cytorhabdovirus rubus*	raspberry vein chlorosis virus (RVCV)
	*Cytorhabdovirus sonchi*	Sonchus virus (SonV)
	*Cytorhabdovirus tagetis*	Tagetes erecta virus 1 (TaEV1)
	*Cytorhabdovirus trachyspermi*	Trachyspermum ammi virus 1 (TrAV1)
	*Cytorhabdovirus trichosanthei*	Trichosanthes associated rhabdovirus 1 (TrARV1)
	*Cytorhabdovirus tritici*	wheat American striate mosaic virus (WASMV)
	*Cytorhabdovirus yerbamate*	yerba mate virus A (YmVA)
*Dichorhavirus*	*Dichorhavirus citri*	citrus chlorotic spot virus (CiCSV)
	*Dichorhavirus clerodendri*	Clerodendrum chlorotic spot virus (ClCSV)
	*Dichorhavirus coffeae*	coffee ringspot virus (CoRSV)
	*Dichorhavirus leprosis*	citrus leprosis virus N (CiLV-N)
	*Dichorhavirus orchidaceae*	orchid fleck virus (OFV)
*Gammanucleorhabdovirus*	*Gammanucleorhabdovirus maydis*	maize fine streak virus (MFSV)
*Varicosavirus*	*Varicosavirus allii*	Allium angulosum virus 1 (AAnV1)
	*Varicosavirus alopecuri*	Alopecurus myosuroides varicosavirus 1 (AMVV1)
	*Varicosavirus brassicae*	Brassica rapa virus 1 (BrRV1)
	*Varicosavirus ipomoeae*	morning glory varicosavirus (MGVV)
	*Varicosavirus lactucae*	lettuce big-vein associated virus (LBVaV)
	*Varicosavirus lolii*	Lolium perenne virus 1 (LoPV1)
	*Varicosavirus melampyri*	Melampyrum roseum virus 1 (MelRoV1)
	*Varicosavirus pini*	Pinus flexilis virus 1 (PiFleV1)
	*Varicosavirus trifolii*	red clover associated varicosavirus (RCaVV)
	*Varicosavirus vitis*	Vitis varicosavirus (VVV)
	*Varicosavirus xinjiangense*	Xinjiang varicosavirus (XVV)
	*Varicosavirus zosterae*	Zostera-associated varicosavirus 1 (ZaVV1)
**Subfamily *Gammarhabdovirinae***		
*Novirhabdovirus*	*Novirhabdovirus hirame*	hirame rhabdovirus (HIRRV=HIRV)
	*Novirhabdovirus piscine*	viral haemorrhagic septicemia virus (VHSV)
	*Novirhabdovirus salmonid*	infectious hematopoietic necrosis virus (IHNV)
	*Novirhabdovirus snakehead*	snakehead rhabdovirus (SHRV)
Unassigned (to subfamilies)		
*Alphacrustrhavirus*	*Alphacrustrhavirus wenling*	Wenling crustacean virus 10 (WlCV10)
	*Alphacrustrhavirus zhejiang*	Wenling crustacean virus 11 (WlCV11)
*Alphadrosrhavirus*	*Alphadrosrhavirus hubei*	Wuhan house fly virus 2 (WhHFV2)
	*Alphadrosrhavirus shayang*	Shayang fly virus 3 (SyFV3)
*Alphahymrhavirus*	*Alphahymrhavirus cinereus*	hymenopteran rhabdo-related virus 38 (HyRRV38)
	*Alphahymrhavirus hirtum*	hymenopteran rhabdo-related virus 109 (HyRRV109)
	*Alphahymrhavirus neglectus*	Lasius neglectus virus 2 (LnegV2)
	*Alphahymrhavirus radians*	hymenopteran rhabdo-related virus 46 (HyRRV46)
*Betahymrhavirus*	*Betahymrhavirus austriaca*	hymenopteran rhabdo-related virus 23 (HyRRV23)
	*Betahymrhavirus heterodontonyx*	hymenopteran rhabdo-related virus 24 (HyRRV24)
*Betanemrhavirus*	*Betanemrhavirus hubei*	Hubei rhabdo-like virus 9 (HbRLV9)
	*Betanemrhavirus shayang*	Shayang ascaridia galli virus 2 (SyAGV2)
*Betapaprhavirus*	*Betapaprhavirus frugiperda*	Spodoptera frugiperda rhabdovirus (SfruRV)
	*Betapaprhavirus sylvina*	lepidopteran rhabdo-related virus 34 (LeRRV34)
*Betaricinrhavirus*	*Betaricinrhavirus chimay*	Chimay rhabdovirus (CRV)
	*Betaricinrhavirus scapularis*	blacklegged tick rhabdovirus 1 (BLTRV1)
**Family *Sunviridae***		
*Sunshinevirus*	*Sunshinevirus reptilis*	Sunshine Coast virus (SunCV)
**Family *Xinmoviridae***		
*Alasvirus*	*Alasvirus muscae*	Húběi diptera virus 11 (HbDV11)
*Anphevirus*	*Anphevirus xinchengense*	Xīnchéng mosquito virus (XcMV)
*Doupovirus*	*Doupovirus australiaense*	Culex mononega-like virus 2 (CMLV2)
*Draselvirus*	*Draselvirus dentati*	Húběi rhabdo-like virus 7 (HbRLV7)
*Drunivirus*	*Drunivirus chambonense*	Drosophila unispina virus 1 (DuniV1)
*Gambievirus*	*Gambievirus bolahunense*	Bolahun virus (BLHV)
	*Gambievirus senegalense*	Gambie virus (GAMV)
*Gylbovirus*	*Gylbovirus aagae*	Aedes anphevirus (AeAV)
*Hoptevirus*	*Hoptevirus orthopteris*	Húběi orthoptera virus 5 (HbOV5)
*Madalivirus*	*Madalivirus amapaense*	Anopheles marajoara virus (AnMV)
	*Madalivirus amazonaense*	Anopheles darlingi virus (AnDV)
*Pelmivirus*	*Pelmivirus eymattense*	hymenopteran anphe-related virus OKIAV71 (HARV71)
*Triniovirus*	*Triniovirus yonagoense*	Culex tritaeniorhynchus anphevirus (CtAV)
*Ulegvirus*	*Ulegvirus freckenfeldense*	odonatan anphe-related virus OKIAV59 (OARV59)

Note that viruses are real objects that are assigned to concepts that are called taxa. Species, genera, subfamilies, families, and orders are taxa. Species, genera, families, and orders are taxa. As appropriate, cultural significance may be conveyed in virus names.

^∗^Includes: Newcastle disease virus (NDV).

†Taxon names are always italicized and always begin with a capital letter.

‡Virus names are not italicized and are not capitalized, except if the name or a name component is a proper noun. This column lists the virus names with their correct (lack of) capitalization. Lists of viruses within a given species are provisional at this point and will likely be amended in the near future.

**Table 5.** ICTV-accepted taxonomy of the order *Goujianvirales* (*Negarnaviricota: Haploviricotina: Yunchangviricetes*) as of April 2023

Genus	Species^∗^	Virus (Abbreviation)†
**Family *Yueviridae***		
*Yuyuevirus*	*Yuyuevirus beihaiense*	Běihǎi sesarmid crab virus 3 (BhSCV3)
	*Yuyuevirus shaheense*	Shāhé yuèvirus-like virus 1 (ShYLV1)

Note that viruses are real objects that are assigned to concepts that are called taxa. Species, genera, families, and orders are taxa. Species, genera, families, and orders are taxa. As appropriate, cultural significance may be conveyed in virus names.

^∗^Taxon names are always italicized and always begin with a capital letter.

†Virus names are not italicized and are not capitalized, except if the name or a name component is a proper noun. This column lists the virus names with their correct (lack of) capitalization.

**Table 6.** ICTV-accepted taxonomy of the order *Bunyavirales* (*Negarnaviricota: Polyploviricotina: Ellioviricetes*) as of April 2023

Genus	Species^∗^	Virus (Abbreviation)†
**Family *Arenaviridae***		
*Antennavirus*	*Antennavirus hirsutum*	Wēnlǐng frogfish arenavirus 2 (WlFAV2)
	*Antennavirus salmonis*	salmon pescarenavirus 1 (SPAV1)
		salmon pescarenavirus 2 (SPAV2)
	*Antennavirus striale*	Wēnlǐng frogfish arenavirus 1 (WlFAV1)
*Hartmanivirus*	*Hartmanivirus brazilense*	SetPatVet virus 1 (SPVV1)
	*Hartmanivirus haartmani*	Haartman Institute snake virus 1 (HISV1)
		Haartman Institute snake virus 2 (HISV2)
	*Hartmanivirus helvetiae*	Dante Muikkunen virus 1 (DaMV1)
	*Hartmanivirus patriae*	andere Heimat virus 1 (aHeV1)
	*Hartmanivirus quadrati*	big electron-dense squares virus 1 (BESV1)
	*Hartmanivirus scholae*	old schoolhouse virus 1 (OScV1)
		old schoolhouse virus 2 (OScV2)
	*Hartmanivirus turici*	veterinary pathology Zurich virus 1 (VPZV1)
		veterinary pathology Zurich virus 2 (VPZV2)
	*Hartmanivirus unni*	Universidad Nacional virus 1 (UnNV1)
*Innmovirus*	*Innmovirus hailarense*	Hailar virus (HLRV)
*Mammarenavirus*	*Mammarenavirus alashanense*	Alxa virus (ALXV)
	*Mammarenavirus allpahuayoense*	Allpahuayo virus (ALLV)
	*Mammarenavirus amapariense*	Amaparí virus (AMAV)
	*Mammarenavirus aporeense*	Aporé virus (APOV)
	*Mammarenavirus bearense*	Bear Canyon virus (BCNV)
	*Mammarenavirus bituense*	Bitu virus (BITV)
	*Mammarenavirus brazilense*	Sabiá virus (SBAV)
	*Mammarenavirus caliense*	Pichindé virus (PICHV)
	*Mammarenavirus cameroonense*	souris virus (SOUV)
	*Mammarenavirus chapareense*	Chapare virus (CHAPV)
	*Mammarenavirus choriomeningitidis*	Dandenong virus (DANV)
		lymphocytic choriomeningitis virus (LCMV)
	*Mammarenavirus cupixiense*	Cupixi virus (CUPXV)
	*Mammarenavirus dhati-welelense*	Dhati Welel virus (DHWV)
	*Mammarenavirus flexalense*	Flexal virus (FLEV)
	*Mammarenavirus gairoense*	Gairo virus (GAIV)
	*Mammarenavirus guanaritoense*	Guanarito virus (GTOV)
	*Mammarenavirus ippyense*	Ippy virus (IPPYV)
	*Mammarenavirus juninense*	Junín virus (JUNV)
	*Mammarenavirus kitaleense*	Kitale virus (KTLV)
	*Mammarenavirus kwanzaense*	Kwanza virus (KWAV)
	*Mammarenavirus lassaense*	Lassa virus (LASV)
	*Mammarenavirus latinum*	Latino virus (LATV)
	*Mammarenavirus lijiangense*	Lìjiāng virus (LIJV)
	*Mammarenavirus loeiense*	Loei River virus (LORV)
	*Mammarenavirus lujoense*	Lujo virus (LUJV)
	*Mammarenavirus lunaense*	Luli virus (LULV)
		Luna virus (LUAV)
	*Mammarenavirus lunkense*	Lunk virus (LNKV)
	*Mammarenavirus machupoense*	Machupo virus (MACV)
	*Mammarenavirus marientalense*	Mariental virus (MRLV)
	*Mammarenavirus merinoense*	Merino Walk virus (MRWV)
	*Mammarenavirus mopeiaense*	Mopeia virus (MPOV)
		Morogoro virus (MORV)
	*Mammarenavirus okahandjaense*	Okahandja virus (OKAV)
	*Mammarenavirus oliverosense*	Oliveros virus (OLVV)
	*Mammarenavirus paranaense*	Paraná virus (PRAV)
	*Mammarenavirus piritalense*	Pirital virus (PIRV)
	*Mammarenavirus praomyidis*	mobala virus (MOBV)
	*Mammarenavirus ryukyuense*	Ryukyu virus (RYKV)
	*Mammarenavirus solweziense*	Solwezi virus (SOLV)
	*Mammarenavirus tacaribeense*	Tacaribe virus (TCRV)
	*Mammarenavirus tamiamiense*	Tamiami virus (TMMV)
	*Mammarenavirus wenzhouense*	Wēnzhōu virus (WENV)
	*Mammarenavirus whitewaterense*	Big Brushy Tank virus (BBRTV)
		Catarina virus (CTNV)
		Skinner Tank virus (SKTV)
		Tonto Creek virus (TTCV)
		Whitewater Arroyo virus (WWAV)
	*Mammarenavirus xapuriense*	Xapuri virus (XAPV)
*Reptarenavirus*	*Reptarenavirus aurei*	Golden Gate virus (GOGV)
	*Reptarenavirus californiae*	CAS virus (CASV)
	*Reptarenavirus commune*	tavallinen suomalainen mies virus 2 (TSMV2)
	*Reptarenavirus giessenae*	University of Giessen virus 1 (UGV1)
		University of Giessen virus 2 (UGV2)
		University of Giessen virus 3 (UGV3)
	*Reptarenavirus rotterdamense*	*ROUT virus (ROUTV)*
		*University of Helsinki virus 1 (UHV1)*
*Family Cruliviridae*		
*Lincruvirus*	*Lincruvirus europense*	*European shore crab virus 1 (EscV1)*
	*Lincruvirus sinense*	*Chinese mitten crab virus 1 (CmcV1)*
	*Lincruvirus wenlingense*	*Wēnlǐng crustacean virus 9 (WlCV9)*
Family Discoviridae		
*Orthodiscovirus*	*Orthodiscovirus coniellae*	Coniothyrium diplodiella negative-stranded RNA virus 1 (CdNSRV1)
		Plasmopara viticola lesion associated mycobunyavirales-like virus 9 (PvLAMLV9)
	*Orthodiscovirus hispaniae*	Plasmopara viticola lesion associated mycobunyavirales-like virus 8 (PvLAMLV8)
	*Orthodiscovirus iberiae*	Plasmopara viticola lesion associated mycobunyavirales-like virus 4 (PvLAMLV4)
	*Orthodiscovirus missouriense*	Penicillium discovirus (PDV)
	*Orthodiscovirus penicillii*	Penicillium roseopurpureum negative ssRNA virus 1 (PrNssV1)
**Family *Fimoviridae***		
*Emaravirus*	*Emaravirus aceris*	maple mottle-associated virus (MaMaV)
	*Emaravirus actinidiae*	Actinidia chlorotic ringspot-associated virus (AcCRaV)
	*Emaravirus cajani*	pigeonpea sterility mosaic virus 1 (PPSMV1)
	*Emaravirus camelliae*	Camellia japonica-associated virus 1 (CjaV1)
	*Emaravirus cercidis*	redbud yellow ringspot-associated virus (RYRaV)
	*Emaravirus chrysanthemi*	chrysanthemum mosaic-associated virus (ChMaV)
	*Emaravirus cordylinae*	ti ringspot-associated virus (TiRSaV)
	*Emaravirus corynocarpi*	karaka Ōkahu purepure emaravirus (KOPV)
	*Emaravirus fici*	fig mosaic virus (FMV)
	*Emaravirus fraxini*	ash shoestring-associated virus (ASaV)
	*Emaravirus idaeobati*	raspberry leaf blotch virus (RLBV)
	*Emaravirus illicii*	Japanese star anise ringspot-associated virus (JSARaV)
	*Emaravirus kiwii*	Actinidia virus 2 (AcV2)
	*Emaravirus parkinsoniae*	palo verde broom virus (PVBV)
	*Emaravirus perillae*	Perilla mosaic virus (PerMV)
	*Emaravirus pistaciae*	Pistacia virus B (PiVB)
	*Emaravirus populi*	aspen mosaic-associated virus (AsMaV)
	*Emaravirus pyri*	pear chlorotic leaf spot-associated virus (PCLSaV)
	*Emaravirus quercus*	common oak ringspot-associated virus (CORaV)
	*Emaravirus rosae*	rose rosette virus (RRV)
	*Emaravirus rubi*	blackberry leaf mottle-associated virus (BLMaV)
	*Emaravirus sorbi*	European mountain ash ringspot-associated virus (EMARaV)
	*Emaravirus syringae*	lilac chlorotic ringspot-associated virus (LiCRaV)
	*Emaravirus toordali*	pigeonpea sterility mosaic virus 2 (PPSMV2)
	*Emaravirus tritici*	High Plains wheat mosaic virus (HPWMoV)
	*Emaravirus verbanni*	Camellia japonica-associated virus 2 (CjaV2)
	*Emaravirus visci*	Arceuthobium sichuanense-associated virus 1 (ArSaV1)
	*Emaravirus vitis*	Vitis emaravirus (VEV)
	*Emaravirus ziziphi*	jujube yellow mottle-associated virus (JYMaV)
**Family *Hantaviridae***		
**Subfamily *Actantavirinae***		
*Actinovirus*	*Actinovirus bernense*	Bern perch virus (BRPV)
	*Actinovirus halieutaeae*	Wēnlǐng minipizza batfish virus (WEMBV)
	*Actinovirus lophii*	Wēnlǐng yellow goosefish virus (WEYGV)
	*Actinovirus triacanthodis*	Wēnlǐng red spikefish virus (WERSV)
**Subfamily *Agantavirinae***		
*Agnathovirus*	*Agnathovirus eptatreti*	Wēnlǐng hagfish virus (WEHV)
Subfamily Mammantavirinae		
*Loanvirus*	*Loanvirus brunaense*	Brno virus (BRNV)
	*Loanvirus longquanense*	Lóngquán virus (LQUV)
*Mobatvirus*	*Mobatvirus laibinense*	Láibīn virus (LAIV)
	*Mobatvirus lenaense*	Lena virus (LENV)
	*Mobatvirus novaense*	Nova virus (NVAV)
	*Mobatvirus quezonense*	Quezon virus (QZNV)
	*Mobatvirus xuansonense*	Xuân Sơn virus (XSV)
*Orthohantavirus*	*Orthohantavirus andesense*	Andes virus (ANDV)
		Castelo dos Sonhos virus (CASV)
		Lechiguanas virus (LECV=LECHV)
		Orán virus (ORNV)
	*Orthohantavirus asamaense*	Asama virus (ASAV)
	*Orthohantavirus asikkalaense*	Asikkala virus (ASIV)
	*Orthohantavirus bayoui*	bayou virus (BAYV)
		Catacamas virus (CATV)
	*Orthohantavirus boweense*	Bowé virus (BOWV)
	*Orthohantavirus brugesense*	Bruges virus (BRGV)
	*Orthohantavirus caobangense*	Cao Bằng virus (CBNV)
		Liánghé virus (LHEV)
	*Orthohantavirus chocloense*	Choclo virus (CHOV)
	*Orthohantavirus dabieshanense*	Dàbiéshān virus (DBSV)
	*Orthohantavirus delgaditoense*	Caño Delgadito virus (CADV)
	*Orthohantavirus dobravaense*	Dobrava virus (DOBV)
		Kurkino virus (KURV)
		Saaremaa virus (SAAV)
		Sochi virus (SOCV)
	*Orthohantavirus fugongense*	Fúgòng virus (FUGV)
	*Orthohantavirus fusongense*	Fǔsōng virus (FUSV)
	*Orthohantavirus hantanense*	Amur virus (AMRV)
		Hantaan virus (HTNV)
		Soochong virus (SOOV)
	*Orthohantavirus jejuense*	Jeju virus (JJUV)
	*Orthohantavirus kenkemeense*	Kenkeme virus (KKMV)
	*Orthohantavirus khabarovskense*	Khabarovsk virus (KHAV)
		Topografov virus (TOPV)
	*Orthohantavirus luxiense*	Lúxī virus (LUXV)
	*Orthohantavirus maporalense*	Maporal virus (MAPV)
	*Orthohantavirus montanoense*	Montaño virus (MTNV)
	*Orthohantavirus moroense*	Carrizal virus (CARV)
		El Moro Canyon virus (ELMCV)
		Huitzilac virus (HUIV)
	*Orthohantavirus necocliense*	Necoclí virus (NECV)
	*Orthohantavirus negraense*	Laguna Negra virus (LANV)
		Maripa virus (MARV)
		Rio Mamoré virus (RIOMV)
	*Orthohantavirus nigrorivense*	Black Creek Canal virus (BCCV)
	*Orthohantavirus oxbowense*	Oxbow virus (OXBV)
	*Orthohantavirus prospectense*	Prospect Hill virus (PHV)
	*Orthohantavirus puumalaense*	Hokkaido virus (HOKV)
		Muju virus (MUJV)
		Puumala virus (PUUV)
	*Orthohantavirus robinaense*	Robina virus (ROBV)
	*Orthohantavirus rockportense*	Rockport virus (RKPV)
	*Orthohantavirus sangassouense*	Sangassou virus (SANGV)
	*Orthohantavirus seewisense*	Seewis virus (SWSV)
	*Orthohantavirus seoulense*	gōu virus (GOUV)
		Seoul virus (SEOV)
	*Orthohantavirus sinnombreense*	New York virus (NYV)
		Sin Nombre virus (SNV)
	*Orthohantavirus tatenalense*	Tatenale virus (TATV)
	*Orthohantavirus thailandense*	Anjozorobe virus (ANJZV)
		Serang virus (SERV)
		Thailand virus (THAIV)
	*Orthohantavirus tigrayense*	Tigray virus (TIGV)
	*Orthohantavirus tulaense*	Adler virus (ADLV)
		Tula virus (TULV)
	*Orthohantavirus yakeshiense*	Yákèshí virus (YKSV)
Thottimvirus	*Thottimvirus imjinense*	Imjin virus (MJNV)
	*Thottimvirus thottapalayamense*	Thottapalayam virus (TPMV)
**Subfamily *Repantavirinae***		
*Reptillovirus*	*Reptillovirus hemidactyli*	Hǎinán oriental leaf-toed gecko virus (HOLGV)
**Family *Leishbuviridae***		
*Shilevirus*	*Shilevirus leptomonadis*	Leptomonas moramango virus (LEPMV)
**Family *Mypoviridae***		
*Hubavirus*	*Hubavirus myriapedis*	Húběi myriapoda virus 5 (HbMV5)
**Family *Nairoviridae***		
*Norwavirus*	*Norwavirus beijiense*	Běijí nairovirus (BJNV)
	*Norwavirus grotenhoutense*	Grotenhout virus (GRHV)
*Ocetevirus*	*Ocetevirus paratemnopterygis*	red goblin roach virus 1 (RGRV1)
*Orthonairovirus*	*Orthonairovirus abuhammadense*	Abū Ḥammād virus (AHV)
	*Orthonairovirus abuminaense*	Abū Mīnā virus (AMV)
	*Orthonairovirus amblyommae*	kupe virus (KUPEV)
	*Orthonairovirus artashatense*	Artashat virus (ARTSV)
	*Orthonairovirus australiaense*	Vinegar Hill virus (VINHV)
	*Orthonairovirus avalonense*	Avalon virus (AVAV)
	*Orthonairovirus bandiaense*	Bandia virus (BDAV)
	*Orthonairovirus buranaense*	Burana virus (BURV)
	*Orthonairovirus bushkeyense*	Caspiy virus (CASV)
		Farallon virus (FARV)
		Great Saltee virus (GRSV)
		Hughes virus (HUGV)
		Raza virus (RAZAV)
	*Orthonairovirus chimense*	Chim virus (CHIMV)
	*Orthonairovirus clomorense*	Clo Mor virus (CMV=CLMV)
	*Orthonairovirus dermacentoris*	Pacific Coast tick nairovirus (PCTNV)
	*Orthonairovirus dugbeense*	Dugbe virus (DUGV)
	*Orthonairovirus erveense*	Erve virus (ERVEV)
	*Orthonairovirus esteroense*	Estero Real virus (ERV)
	*Orthonairovirus gossasense*	Gossas virus (GOSV)
	*Orthonairovirus haemorrhagiae*	Crimean-Congo haemorrhagic fever virus (CCHFV)
	*Orthonairovirus hazaraense*	Hazara virus (HAZV)
	*Orthonairovirus huangpiense*	Huángpí tick virus 1 (HpTV1)
	*Orthonairovirus issykkulense*	Issyk-kul virus (ISKV)
	*Orthonairovirus japonicum*	tofla virus (TFLV)
	*Orthonairovirus kasokeroense*	Kasokero virus (KASV=KASOV)
	*Orthonairovirus keterehense*	Keterah virus (KTRV)
		Uzun-Agach virus (UZAV)
	*Orthonairovirus khani*	Dera Ghazi Khan virus (DGKV)
	*Orthonairovirus lusakaense*	Leopards Hill virus (LPHV)
	*Orthonairovirus macquariense*	Taggert virus (TAGV)
	*Orthonairovirus meramense*	Meram virus (MEMV)
	*Orthonairovirus nairobiense*	Nairobi sheep disease virus (NSDV)
	*Orthonairovirus parahaemorrhagiae*	Aigai virus (AIGV)
	*Orthonairovirus peruense*	Punta Salinas virus (PSV)
	*Orthonairovirus qalyubense*	Geran virus (GERV)
		Qalyub virus (QYBV)
	*Orthonairovirus randallense*	Sapphire II virus (SAPV)
	*Orthonairovirus sakhalinense*	Sakhalin virus (SAKV)
		Tillamook virus (TILLV)
	*Orthonairovirus soldadoense*	Soldado virus (SOLV)
	*Orthonairovirus songlingense*	Sōnglǐng virus (SGLV)
	*Orthonairovirus sulinaense*	Sulina virus (SULV)
	*Orthonairovirus tachengense*	Tǎchéng tick virus 1 (TcTV1)
	*Orthonairovirus thiaforaense*	Thiafora virus (TFAV)
	*Orthonairovirus tomdiense*	Tamdy virus (TAMV)
	*Orthonairovirus tunisense*	Tunis virus (TUNV)
	*Orthonairovirus wenzhouense*	Wēnzhōu tick virus (WzTV)
	*Orthonairovirus yezoense*	Yezo virus (YEZV)
	*Orthonairovirus yogueense*	Yogue virus (YOGV)
	*Orthonairovirus zirkuense*	Zirqa virus (ZIRV)
*Sabavirus*	*Sabavirus americanum*	South Bay virus (SBV)
*Shaspivirus*	*Shaspivirus aranei*	Shāyáng spider virus 1 (SySV1)
*Striwavirus*	*Striwavirus sanxiaense*	Sānxiá water strider virus 1 (SxWSV1)
*Xinspivirus*	*Xinspivirus xinzhouense*	Xīnzhōu spider virus (XSV)
**Family *Peribunyaviridae***		
*Herbevirus*	*Herbevirus herberti*	Herbert virus (HEBV)
	*Herbevirus kibaleense*	Kibale virus (KIBV)
	*Herbevirus taiense*	Taï virus (TAIV)
*Khurdivirus*	*Khurdivirus volgaense*	Khurdun virus (KURV)
*Lakivirus*	*Lakivirus lakamhaense*	Lakamha virus (LAKV)
*Lambavirus*	*Lambavirus wisconsinense*	largemouth bass bunyavirus (LBBV)
*Orthobunyavirus*	*Orthobunyavirus abrasense*	Abras virus (ABRV)
	*Orthobunyavirus acaraense*	Acará virus (ACAV)
		Benevides virus (BVSV=BENV)
	*Orthobunyavirus achiotei*	Achiote virus (ACHOV)
		Trivittatus virus (TVTV)
	*Orthobunyavirus ainoense*	Aino virus (AINOV)
		Kaikalur virus (KAIV)
	*Orthobunyavirus akabaneense*	Akabane virus (AKAV)
		Tinaroo virus (TINV)
	*Orthobunyavirus anadyrense*	Anadyr virus (ANADV)
	*Orthobunyavirus ananindeuaense*	Ananindeua virus (ANUV)
	*Orthobunyavirus angeloense*	San Angelo virus (SAV)
	*Orthobunyavirus anhembiense*	Anhembi virus (AMBV)
	*Orthobunyavirus apeuense*	Apeú virus (APEUV)
	*Orthobunyavirus baakalense*	Baakal virus (BKAV)
	*Orthobunyavirus bakauense*	Bakau virus (BAKV)
		Tanjong Rabok virus (TRV)
	*Orthobunyavirus balagoduense*	Balagodu virus (BLGV)
	*Orthobunyavirus bataiense*	Batai virus (BATV)
	*Orthobunyavirus batamaense*	Batama virus (BMAV)
	*Orthobunyavirus belemense*	Belém virus (BLMV)
	*Orthobunyavirus bellavistaense*	Antequera virus (ANTV)
		Barranqueras virus (BQSV)
		Bellavista virus (BELLV)
		Resistencia virus (RTAV)
	*Orthobunyavirus benficaense*	Benfica virus (BENV=BNFV)
	*Orthobunyavirus bertiogaense*	Bertioga virus (BERV)
		Cananéia virus (CNAV)
	*Orthobunyavirus biraoense*	Birao virus (BIRV)
	*Orthobunyavirus bobayaense*	Bobaya virus (BOBV)
	*Orthobunyavirus boraceiaense*	Boracéia virus (BORV)
	*Orthobunyavirus botambiense*	Botambi virus (BOTV)
	*Orthobunyavirus bozoense*	Bozo virus (BOZOV)
	*Orthobunyavirus brazoriaense*	brazoran virus (BRAZV)
	*Orthobunyavirus bruconhaense*	Bruconha virus (BRUV)
	*Orthobunyavirus buffaloense*	Buffalo Creek virus (BUCV)
		Murrumbidgee virus (MURBV)
		Trubanaman virus (TRUV)
	*Orthobunyavirus bunyamweraense*	Bunyamwera virus (BUNV)
		Germiston virus (GERV)
		Mboké virus (MBOV)
		Ngari virus (NRIV)
		Stanfield virus (STAV)
		Xingu virus (XINV)
	*Orthobunyavirus bushbushense*	Bushbush virus (BSBV)
		Moriche virus (MORV)
	*Orthobunyavirus buttonwillowense*	Buttonwillow virus (BUTV)
	*Orthobunyavirus bwambaense*	Bwamba virus (BWAV)
		Pongola virus (PGAV)
	*Orthobunyavirus cacheense*	Cache Valley virus (CVV)
		Cholul virus (CHLV)
		Playas virus (PLAV)
		Tlacotalpan virus (TLAV)
	*Orthobunyavirus capimense*	Capim virus (CAPV)
	*Orthobunyavirus caraparuense*	Caraparú virus (CARV)
		El Huayo virus (EHUV)
		Itaquí virus (ITQV)
		Itaya virus (ITYV)
		Ossa virus (OSSAV)
	*Orthobunyavirus catqueense*	Cát Quế virus (CQV)
		Oya virus (OYAV)
	*Orthobunyavirus catuense*	Catú virus (CATUV)
		Moju virus (MOJUV)
	*Orthobunyavirus cuchillaense*	Anopheles B virus (ANBV)
	*Orthobunyavirus ebiense*	Ebinur Lake virus (EBIV)
	*Orthobunyavirus encephalitidis*	California encephalitis virus (CEV)
		Morro Bay virus (MBV)
	*Orthobunyavirus enseadaense*	Enseada virus (ENSV)
	*Orthobunyavirus faceyense*	Facey’s paddock virus (FPV)
	*Orthobunyavirus gamboaense*	Alajuela virus (ALJV)
		Brus Laguna virus (BLAV)
		Calchaquí virus (CQIV)
		Gamboa virus (GAMV)
		Soberanía virus (SOBV)
	*Orthobunyavirus ganganense*	Gan Gan virus (GGV)
		Salt Ash virus (SASHV)
	*Orthobunyavirus guajaraense*	Guajará virus (GJAV)
	*Orthobunyavirus guamaense*	Bimiti virus (BIMV)
		Guamá virus (GMAV)
	*Orthobunyavirus guaratubaense*	Guaratuba virus (GTBV)
		Itimirim virus (ITIV)
	*Orthobunyavirus guaroaense*	Guaroa virus (GROV)
	*Orthobunyavirus gumbolimboense*	Gumbo Limbo virus (GLV)
	*Orthobunyavirus heptayabaense*	Yaba-7 virus (Y7V)
	*Orthobunyavirus horizonteense*	Anopheles A virus (ANAV)
		Arumateua virus (ARTV=ARMTV)
		Caraipé virus (CPEV=CRPV)
		Trombetas virus (TRMV)
		Tucuruí virus (TUCV=TUCRV)
	*Orthobunyavirus iacoense*	Iaco virus (IACOV)
	*Orthobunyavirus ileshaense*	Ilesha virus (ILEV)
	*Orthobunyavirus infirmati*	Infirmatus virus (INFV)
	*Orthobunyavirus ingwavumaense*	Ingwavuma virus (INGV)
	*Orthobunyavirus insulae*	kairi virus (KRIV)
	*Orthobunyavirus jamestownense*	Inkoo virus (INKV)
		Jamestown Canyon virus (JCV)
		Jerry Slough virus (JSV)
		South River virus (SORV)
	*Orthobunyavirus jatobalense*	Jatobal virus (JATV)
	*Orthobunyavirus juandiazense*	Juan Díaz virus (JDV)
	*Orthobunyavirus kaengkhoiense*	Kaeng Khoi virus (KKV)
	*Orthobunyavirus kernense*	Lokern virus (LOKV)
		Main Drain virus (MDV)
		Santa Rosa virus (SARV)
	*Orthobunyavirus ketapangense*	Ketapang virus (KETV)
	*Orthobunyavirus keystoneense*	Keystone virus (KEYV)
	*Orthobunyavirus khatangaense*	Khatanga virus (KHATV)
		snowshoe hare virus (SSHV)
	*Orthobunyavirus koongoli*	koongol virus (KOOV)
		wongal virus (WONV)
	*Orthobunyavirus kowanyamaense*	Kowanyama virus (KOWV)
	*Orthobunyavirus lacrosseense*	La Crosse virus (LACV)
	*Orthobunyavirus lasmaloyasense*	Las Maloyas virus (LMV)
	*Orthobunyavirus leanyerense*	Leanyer virus (LEAV)
	*Orthobunyavirus ledniceense*	Lednice virus (LEDV)
	*Orthobunyavirus lukuniense*	Lukuni virus (LUKV)
	*Orthobunyavirus lumboense*	Lumbo virus (LUMV)
	*Orthobunyavirus macauaense*	Macauã virus (MCAV)
	*Orthobunyavirus madridense*	Madrid virus (MADV)
		Vinces virus (VINV)
	*Orthobunyavirus maguariense*	Maguari virus (MAGV)
	*Orthobunyavirus mahoganyense*	Mahogany Hammock virus (MHV)
	*Orthobunyavirus manzanillaense*	Manzanilla virus (MANV)
		Inini virus (INIV)
	*Orthobunyavirus maprikense*	Maprik virus (MPKV)
	*Orthobunyavirus maritubaense*	Marituba virus (MTBV)
		Zungarococha virus (ZUNV)
	*Orthobunyavirus matruhense*	Matruh virus (MTRV)
	*Orthobunyavirus melajoense*	Melao virus (MELV)
	*Orthobunyavirus mermetense*	Mermet virus (MERV)
	*Orthobunyavirus minatitlanense*	Minatitlán virus (MNTV)
		Palestina virus (PLSV)
	*Orthobunyavirus mirimense*	Mirim virus (MIRV)
	*Orthobunyavirus mitchellense*	Mapputta virus (MAPV)
	*Orthobunyavirus mpokoense*	M'Poko virus (MPOV)
		Yaba-1 virus (Y1V)
	*Orthobunyavirus navioense*	Serra do Navio virus (SDNV)
	*Orthobunyavirus nepuyoi*	Nepuyo virus (NEPV)
	*Orthobunyavirus nesszionaense*	Ness Ziona virus (NZV)
	*Orthobunyavirus nolaense*	Nola virus (NOLAV)
	*Orthobunyavirus northwayense*	Northway virus (NORV)
	*Orthobunyavirus nyandoense*	Eretmapodites virus (ERETV)
		Mojuí dos Campos virus (MDCV)
		Nyando virus (NDV)
	*Orthobunyavirus okolaense*	Okola virus (OKOV)
	*Orthobunyavirus olifantsvleiense*	Bobia virus (BIAV)
		Dabakala virus (DABV)
		Olifantsvlei virus (OLIV)
		Oubi virus (OUBIV)
	*Orthobunyavirus oribocaense*	Murutucú virus (MURV)
		Oriboca virus (ORIV)
		Restan virus (RESV)
	*Orthobunyavirus oropoucheense*	Iquitos virus (IQTV)
		Madre de Dios virus (MDDV)
		Oropouche virus (OROV)
		Perdões virus (PDEV)
		Pintupo virus (PINTV)
	*Orthobunyavirus oyoense*	Oyo virus (OYOV)
	*Orthobunyavirus pacoraense*	Pacora virus (PCAV)
	*Orthobunyavirus patoisense*	Babahoya virus (BABV)
		Pahayokee virus (PAHV)
		Patois virus (PATV)
	*Orthobunyavirus peachesterense*	peaton virus (PEAV)
	*Orthobunyavirus porteiraense*	Cachoeira Porteira virus (CPOV)
	*Orthobunyavirus potosiense*	Potosi virus (POTV)
	*Orthobunyavirus saboense*	Sabo virus (SABOV)
	*Orthobunyavirus sangoense*	Sango virus (SANV)
	*Orthobunyavirus sanjuanense*	Pueblo Viejo virus (PVV)
		San Juan virus (SJV)
	*Orthobunyavirus schmallenbergense*	Douglas virus (DOUV)
		Sathuperi virus (SATV)
		Schmallenberg virus (SBV)
		Shamonda virus (SHAV)
	*Orthobunyavirus sedlecense*	Sedlec virus (SEDV)
	*Orthobunyavirus shermanense*	Fort Sherman virus (FSV)
		Laguna Larga virus (LLV)
	*Orthobunyavirus shokweense*	Shokwe virus (SHOV)
	*Orthobunyavirus shuniense*	Shuni virus (SHUV)
	*Orthobunyavirus simbuense*	Para virus (PARAV)
		Simbu virus (SIMV)
	*Orthobunyavirus sororocaense*	Sororoca virus (SORV)
	*Orthobunyavirus squalofluvii*	Shark River virus (SRV)
	*Orthobunyavirus tacaiumaense*	CoAr 1071 virus (CA1071V)
		CoAr 3627 virus (CA3626V)
		Tacaiuma virus (TCMV)
		Virgin River virus (VRV)
	*Orthobunyavirus tahynaense*	Ťahyňa virus (TAHV)
	*Orthobunyavirus tangaense*	Tanga virus (TANV)
	*Orthobunyavirus tataguineense*	Tataguine virus (TATV)
	*Orthobunyavirus telokense*	Telok Forest virus (TFV)
	*Orthobunyavirus tensawense*	Tensaw virus (TENV)
	*Orthobunyavirus termeilense*	Termeil virus (TERV)
	*Orthobunyavirus teteense*	Bahig virus (BAHV)
		Tete virus (TETEV)
		Tsuruse virus (TSUV)
		Weldona virus (WELV)
	*Orthobunyavirus thimiriense*	Thimiri virus (THIV)
	*Orthobunyavirus timboteuaense*	Timboteua virus (TBTV)
	*Orthobunyavirus trinitiense*	Triniti virus (TNTV)
	*Orthobunyavirus turlockense*	Turlock virus (TURV)
	*Orthobunyavirus umbreense*	Umbre virus (UMBV)
	*Orthobunyavirus utingaense*	Utinga virus (UTIV)
	*Orthobunyavirus witwatersrandense*	Witwatersrand virus (WITV)
	*Orthobunyavirus wolkbergense*	Wolkberg virus (WBV)
	*Orthobunyavirus wyeomyiae*	Rio Pracupi virus
		Taiassui virus (TAIAV)
		Tucunduba virus (TUCV)
		Wyeomyia virus (WYOV)
*Pacuvirus*	*Pacuvirus caimitoense*	Caimito virus (CAIV)
	*Pacuvirus chilibreense*	Chilibre virus (CHIV)
	*Pacuvirus evaense*	Rio Preto da Eva virus (RPEV)
	*Pacuvirus pacuiense*	Pacui virus (PACV)
	*Pacuvirus tapirapeense*	Tapirapé virus (TAPV)
*Shangavirus*	*Shangavirus shuangaoense*	Shuāngào insect virus 1 (SgIV1)
**Family *Phasmaviridae***		
*Cicadellivirus*	*Cicadellivirus scaphoidei*	Scaphoideus titanus bunya-like virus 1 (StHV)
*Feravirus*	*Feravirus ferakinum*	Ferak virus (FRKV)
	*Feravirus guaguaense*	Guagua virus (GUAV)
	*Feravirus hemipterus*	hemipteran phasma-related virus OKIAV247 (HeFV)
	*Feravirus neuropterus*	neuropteran phasma-related virus OKIAV248 (NeFV)
*Hymovirus*	*Hymovirus chrysis*	hymenopteran phasma-related virus OKIAV250 (HyHV2)
	*Hymovirus chrysurae*	hymenopteran phasma-related virus OKIAV252 (HyHV1)
*Jonvirus*	*Jonvirus eboris*	jonchet virus (JONV)
*Orthophasmavirus*	*Orthophasmavirus aedis*	Wǔhàn mosquito virus 2 (WhMV2)
	*Orthophasmavirus anophelae*	Anopheles triannulatus orthophasmavirus (AtOPV)
	*Orthophasmavirus barstukasense*	Barstukas virus (BARV)
	*Orthophasmavirus chrysis*	hymenopteran phasma-related virus OKIAV227 (HyOV2)
	*Orthophasmavirus coleopterus*	coleopteran phasma-related virus OKIAV235 (CPRV)
	*Orthophasmavirus coredoense*	Coredo virus (CORV)
	*Orthophasmavirus culicis*	Culex phasma-like virus (CPLV)
	*Orthophasmavirus flenense*	Flen virus (FLNV)
	*Orthophasmavirus fushunense*	Fǔshùn phasmavirus 1 (FsnPV1)
	*Orthophasmavirus gandaense*	Ganda bee virus (GBEEV)
	*Orthophasmavirus hubeiense*	Húběi odonate virus 9 (HbOV9)
	*Orthophasmavirus kigluaikense*	Kigluaik phantom virus (KIGV)
	*Orthophasmavirus miglotasense*	Miglotas virus (MIGV)
	*Orthophasmavirus niuklukense*	Niukluk phantom virus (NUKV)
	*Orthophasmavirus odonatus*	Húběi odonate virus 8 (HbOV8)
	*Orthophasmavirus philoctetis*	hymenopteran phasma-related virus OKIAV228 (HyOV1)
	*Orthophasmavirus sogatellae*	Fǔshùn phasmavirus 2 (FsnPV2)
	*Orthophasmavirus wuchangense*	Wǔchāng cockroach virus 1 (WcCV1)
	*Orthophasmavirus wuhanense*	Wǔhàn mosquito virus 1 (WhMV1)
*Sawastrivirus*	*Sawastrivirus sanxiaense*	Sānxiá water strider virus 2 (SxWSV2)
*Wuhivirus*	*Wuhivirus insecti*	Wǔhàn insect virus 2 (WhIV2)
**Family *Phenuiviridae***		
*Bandavirus*	*Bandavirus albatrossense*	Albatross Island virus (ABIV)
	*Bandavirus amblyommae*	lone star virus (LSV)
	*Bandavirus bhanjanagarense*	Bhanja virus (BHAV)
	*Bandavirus dabieense*	severe fever with thrombocytopenia syndrome virus (SFTSV)
	*Bandavirus guertuense*	Gùěrtú virus (GTV)
	*Bandavirus heartlandense*	Heartland virus (HRTV)
	*Bandavirus kismaayoense*	Kismaayo virus (KISV)
	*Bandavirus razdanense*	Razdan virus (RAZV)
	*Bandavirus zwieselense*	Zwiesel bat bandavirus (ZbbV)
*Beidivirus*	*Beidivirus muscae*	Húběi diptera virus 3 (HbDV3)
*Citricivirus*	*Citricivirus chongqinense*	Aphis citricidus bunyavirus (AcBV)
*Coguvirus*	*Coguvirus chinense*	Brassica campestris chinensis coguvirus 1 (BCCoV1)
	*Coguvirus citri*	citrus concave gum-associated virus (CCGaV)
	*Coguvirus citrulli*	watermelon crinkle leaf-associated virus 1 (WCLaV1)
	*Coguvirus eburi*	citrus virus A (CiVA)
	*Coguvirus henanense*	watermelon crinkle leaf-associated virus 2 (WCLaV2)
	*Coguvirus viticulum*	grapevine associated cogu-like virus 1 (GaCLV1)
	*Coguvirus yunnanense*	Yúnnán Paris negative-stranded virus (YPNSV)
*Entovirus*	*Entovirus entoleucae*	Entoleuca phenui-like virus 1 (EnPLV1)
*Goukovirus*	*Goukovirus aphalarae*	Aphalara polygoni bunya-like virus (ApBLV)
	*Goukovirus ceraphri*	Ceraphron bunya-like virus (CerBLV)
	*Goukovirus cumutoense*	Cumuto virus (CUMV)
	*Goukovirus gouleakoense*	Gouléako virus (GOLV)
	*Goukovirus yichangense*	Yíchāng insect virus (YcIV)
*Horwuvirus*	*Horwuvirus fitzroyense*	Fitzroy Crossing tenui-like virus 1 (FCTenV1)
	*Horwuvirus solenopsidis*	Solenopsis invicta virus 14 (SINV14)
	*Horwuvirus wuhanense*	Wǔhàn horsefly virus (WhHV)
*Hudivirus*	*Hudivirus muscae*	Húběi diptera virus 4 (HbDV4)
*Hudovirus*	*Hudovirus lepidopteris*	Húběi lepidoptera virus 1 (HbLV1)
*Ixovirus*	*Ixovirus heckscherense*	blacklegged tick virus 1 (BLTV1)
	*Ixovirus ixodis*	blacklegged tick virus 3 (BLTV3)
	*Ixovirus norvegiae*	Fairhair virus (FHAV)
*Laulavirus*	*Laulavirus alphaviticulum*	grapevine associated cogu-like virus 2 (GaCLV2)
	*Laulavirus betaviticulum*	grapevine associated cogu-like virus 3 (GaCLV3)
	*Laulavirus gammaviticulum*	grapevine associated cogu-like virus 4 (GaCLV4)
	*Laulavirus laurelense*	Laurel Lake virus (LLV)
	*Laulavirus wardellense*	Wardell virus (WRDV)
*Lentinuvirus*	*Lentinuvirus lentinulae*	Lentinula edodes negative-strand RNA virus 2 (LeNSRV2)
*Mechlorovirus*	*Mechlorovirus cucumeris*	melon chlorotic spot virus (MeCSV)
*Mobuvirus*	*Mobuvirus arnae*	Euproctis pseudoconspersa bunyavirus (EPBV)
	*Mobuvirus mothrae*	Mothra virus (MTHV)
	*Mobuvirus narangueense*	Ñarangue virus (NRGV)
	*Mobuvirus stephanocirci*	Browner virus (BRWV)
*Phasivirus*	*Phasivirus baduense*	Badu virus (BADUV)
	*Phasivirus guadeloupeense*	Guadeloupe mosquito phasivirus (GMPV)
	*Phasivirus hubeiense*	Húběi diptera virus 5 (HbDV5)
	*Phasivirus parryense*	Parry’s Creek phasivirus 1 (PCPhasV1)
	*Phasivirus phasiense*	Phasi Charoen-like virus (PCLV)
	*Phasivirus wuhanense*	Wǔhàn fly virus 1 (WhFV1)
	*Phasivirus wutaiense*	Wǔtái mosquito virus (WtMV)
*Phlebovirus*	*Frijoles phlebovirus*	frijoles virus (FRIV)
		Joá virus (JOAV)
	*Phlebovirus adanaense*	Adana virus (ADAV)
	*Phlebovirus aguacateense*	Aguacate virus (AGUV)
	*Phlebovirus alcubeense*	Alcube virus (ACBV)
	*Phlebovirus alenquerense*	Alenquer virus (ALEV)
	*Phlebovirus almendrasense*	Tres Almendras virus (TRAV)
	*Phlebovirus ambeense*	Ambé virus (ABEV)
	*Phlebovirus anhangaense*	Anhangá virus (ANHV)
	*Phlebovirus arumowotense*	Arumowot virus (AMTV)
	*Phlebovirus bogoriaense*	Bogoria virus (BGRV)
	*Phlebovirus buenaventuraense*	Buenaventura virus (BUEV)
	*Phlebovirus bujaruense*	Bujaru virus (BUJV)
	*Phlebovirus cacaoense*	Cacao virus (CACV)
	*Phlebovirus campanaense*	Campana virus (CMAV)
	*Phlebovirus candiruense*	Ariquemes virus (ARQV)
		Candirú virus (CDUV)
		Jacundá virus (JCNV)
		Morumbi virus (MR(M)BV)
		Mucura virus (MCRV=MRAV)
		Serra Norte virus (SRNV)
	*Phlebovirus chagresense*	Chagres virus (CHGV)
	*Phlebovirus claroense*	Rio Claro virus (RICV)
	*Phlebovirus cocleense*	Coclé virus (CCLV)
	*Phlebovirus corfouense*	Corfou virus (CFUV)
	*Phlebovirus dashliense*	Dāshlī virus (DASV)
	*Phlebovirus duraniaense*	Durania virus (DRNV)
	*Phlebovirus echarateense*	Echarate virus (ECHV)
	*Phlebovirus embossosense*	Embossos virus (EMBV)
	*Phlebovirus florisense*	Saint-Floris virus (SAFV)
	*Phlebovirus gabekense*	Gabek forest virus (GFV)
	*Phlebovirus gloriaense*	La Gloria virus (LAGV)
	*Phlebovirus gordilense*	Gordil virus (GORV)
	*Phlebovirus hediense*	Hédǐ virus (HEDV)
	*Phlebovirus icoaraciense*	Icoaraci virus (ICOV)
	*Phlebovirus itaitubaense*	Itaituba virus (ITAV)
	*Phlebovirus itaporangaense*	Itaporanga virus (ITPV)
	*Phlebovirus ixcanalense*	Ixcanal virus (IXCV)
	*Phlebovirus karimabadense*	Karimabad virus (KARV)
	*Phlebovirus kiborgochense*	Kiborgoch virus (KBGV)
	*Phlebovirus leticiaense*	Leticia virus (LTCV)
	*Phlebovirus maldonadoense*	Maldonado virus (MLOV)
	*Phlebovirus massiliaense*	Massilia virus (MASV)
	*Phlebovirus medjerdaense*	Medjerda Valley virus (MVV)
	*Phlebovirus monagritaense*	Mona Grita virus (MOGV)
	*Phlebovirus mukawaense*	Mukawa virus (MKWV)
	*Phlebovirus mungubaense*	Munguba virus (MUNV)
	*Phlebovirus napoliense*	Arrábida virus (ARRV)
		Balkan virus (BALKV)
		Fermo virus (FERV)
		Granada virus (GRV=GRAV)
		Saddaguia virus (SADV)
		sandfly fever Naples virus (SFNV)
	*Phlebovirus niqueense*	Nique virus (NIQV)
	*Phlebovirus ntepesense*	Ntepes virus (NTPV)
	*Phlebovirus odrenisrouense*	Odrénisrou virus (ODRV)
	*Phlebovirus oriximinaense*	Oriximiná virus (ORXV)
	*Phlebovirus pantanalense*	viola virus (VIOV)
	*Phlebovirus penablancaense*	Peña Blanca virus (PEBV)
	*Phlebovirus penshurtense*	Penshurt virus (PEHV)
	*Phlebovirus perkerraense*	Perkerra virus (PKEV)
	*Phlebovirus puniqueense*	Punique virus (PUNV)
	*Phlebovirus riftense*	Rift Valley fever virus (RVFV)
	*Phlebovirus riograndense*	Rio Grande virus (RGV)
	*Phlebovirus salangaense*	Salanga virus (SLGV)
	*Phlebovirus salehabadense*	Adria virus (ADRV)
		Arbia virus (ARBV)
		Bregalaka virus (BREV)
		Olbia virus (OLBV)
		Salehabad virus (SALV)
		Zaba virus (ZABAV)
	*Phlebovirus saloboense*	Salobo virus (SLBOV)
	*Phlebovirus siciliaense*	sandfly fever Sicilian virus (SFSV)
	*Phlebovirus taparaense*	Tapará virus (TPRV)
	*Phlebovirus tehranense*	Tehran virus (THEV)
	*Phlebovirus ticoense*	Tico virus (TICV)
	*Phlebovirus toroense*	Capira virus (CAPIV)
		Punta Toro virus (PTV)
	*Phlebovirus torosense*	Toros virus (TORV)
	*Phlebovirus toscanaense*	Toscana virus (TOSV)
	*Phlebovirus turunaense*	Turuna virus (TUAV)
	*Phlebovirus uriuranaense*	Uriurana virus (URIV)
	*Phlebovirus urucuriense*	Urucuri virus (URUV)
	*Phlebovirus zerdaliense*	Zerdali virus (ZERV)
*Pidchovirus*	*Pidchovirus pidgei*	Pidgey virus (PGYV)
	*Pidchovirus stethori*	coleopteran phenui-related virus 308 (CoPrV308)
*Rubodvirus*	*Rubodvirus argentinaense*	grapevine Muscat rose virus (GMRV)
	*Rubodvirus armeniaense*	grapevine Garan dmak virus (GGDV)
	*Rubodvirus mali*	apple rubbery wood virus 1 (ARWV1)
	*Rubodvirus prosserense*	apple rubbery wood virus 2 (ARWV2)
*Tanzavirus*	*Tanzavirus daressalaamense*	Dar es Salaam virus (DeSV)
*Tenuivirus*	*Tenuivirus echinochloae*	Echinochloa hoja blanca virus (EHBV)
	*Tenuivirus eurotritici*	European wheat striate mosaic virus (EWSMV)
	*Tenuivirus oryzabrevis*	rice grassy stunt virus (RGSV)
	*Tenuivirus oryzaclavatae*	rice stripe virus (RSV=RStV)
	*Tenuivirus oryzalbae*	rice hoja blanca virus (RHBV)
	*Tenuivirus persotritici*	Iranian wheat stripe virus (IWSV)
	*Tenuivirus urochloae*	Urochloa hoja blanca virus (UHBV)
	*Tenuivirus zeae*	maize stripe virus (MSV=MStV= MSpV)
*Uukuvirus*	*Uukuvirus dabieshanense*	Dàbiéshān tick virus (DbsTV=DBTV= DTV)
	*Uukuvirus dermacentoris*	American dog tick virus (ADAV)
	*Uukuvirus grandarbaudense*	Grand Arbaud virus (GAV)
	*Uukuvirus hoplandense*	Pacific coast tick virus (PACV)
	*Uukuvirus huangpiense*	Huángpí tick virus 2 (HpTV2)
	*Uukuvirus kabutoense*	Kabuto mountain virus (KAMV)
	*Uukuvirus kaisodiense*	Kaisodi virus (KASDV)
	*Uukuvirus lihanense*	Lǐhán tick virus (LITV)
	*Uukuvirus macquariense*	Precarious Point virus (PPV)
	*Uukuvirus rukutamaense*	Rukutama virus (RUKV)
	*Uukuvirus schmidti*	Nile warbler virus (NIWV)
	*Uukuvirus silverwaterense*	Silverwater virus (SILV)
	*Uukuvirus tachengense*	Tǎchéng tick virus 2 (TcTV2)
	*Uukuvirus toyoense*	Tōyo virus (TOYOV)
	*Uukuvirus tyulenyense*	Zaliv Terpeniya virus (ZTV)
	*Uukuvirus uriae*	murre virus (MURV)
	*Uukuvirus uukuniemiense*	Chizé virus (CHZV)
		Fin V 707 virus (FINV)
		Oceanside virus (OCV=OCEV)
		Pontevès virus (PTVV)
		St. Abbs Head virus (SAHV)
		Uukuniemi virus (UUKV)
	*Uukuvirus yongjiaense*	Yǒngjiā tick virus (YONV)
*Wenrivirus*	*Wenrivirus penaei*	Mourilyan virus (MoV)
**Family *Tospoviridae***		
*Orthotospovirus*	*Orthotospovirus alstroemeriflavi*	Alstroemeria yellow spot virus (AYSV)
	*Orthotospovirus alstroemerinecrosis*	Alstroemeria necrotic streak virus (ANSV)
	*Orthotospovirus arachianuli*	groundnut ringspot virus (GRSV)
	*Orthotospovirus arachiflavamaculae*	groundnut yellow spot virus (GYSV)
	*Orthotospovirus arachiflavi*	groundnut chlorotic fan-spot virus (GCFSV)
	*Orthotospovirus arachinecrosis*	groundnut bud necrosis virus (GBNV)
	*Orthotospovirus callaflavi*	calla lily chlorotic spot virus (CCSV)
	*Orthotospovirus capsiciflavi*	Capsicum chlorosis virus (CaCV)
	*Orthotospovirus capsicimaculaflavi*	pepper chlorotic spot virus (PCSV)
	*Orthotospovirus chrysanthinecrocaulis*	Chrysanthemum stem necrosis virus (CSNV)
	*Orthotospovirus citrullomaculosi*	watermelon silver mottle virus (WSMoV)
	*Orthotospovirus citrullonecrosis*	watermelon bud necrosis virus (WBNV)
	*Orthotospovirus cucurbichlorosis*	zucchini lethal chlorosis virus (ZLCV)
	*Orthotospovirus glycininecrovenae*	soybean vein necrosis virus (SVNV)
	*Orthotospovirus hippeflavi*	Hippeastrum chlorotic spot virus (HCRV)
	*Orthotospovirus impatiensnecromaculae*	impatiens necrotic spot virus (INSV)
	*Orthotospovirus iridimaculaflavi*	iris yellow spot virus (IYSV)
	*Orthotospovirus meloflavi*	melon yellow spot virus (MYSV)
	*Orthotospovirus melotessellati*	melon severe mosaic virus (MSMV)
	*Orthotospovirus morivenae*	mulberry vein banding-associated virus (MVBaV)
	*Orthotospovirus phaseolinecrotessellati*	bean necrotic mosaic virus (BeNMV)
	*Orthotospovirus polygonianuli*	Polygonum ringspot virus (PolRSV)
	*Orthotospovirus tomatanuli*	tomato yellow ring virus (TYRV)
	*Orthotospovirus tomatoflavi*	tomato chlorotic spot virus (TCSV)
	*Orthotospovirus tomatomaculae*	tomato spotted wilt virus (TSWV)
	*Orthotospovirus tomatozonae*	tomato zonate spot virus (TZSV)
**Family *Tulasviridae***		
*Orthotulasvirus*	*Orthotulasvirus tulasnellae*	Tulasnella bunyavirales-like virus 1 (TB-LV)
**Family *Wupedeviridae***		
*Wumivirus*	*Wumivirus millepedae*	Wǔhàn millipede virus 2 (WhMV2)

Note that viruses are real objects that are assigned to concepts that are called taxa. Species, genera, subfamilies, families, and orders are taxa. Species, genera, families, and orders are taxa. As appropriate, cultural significance may be conveyed in virus names.

^∗^Taxon names are always italicized and always begin with a capital letter.

†Virus names are not italicized and are not capitalized, except if the name or a name component is a proper noun. This column lists the virus names with their correct (lack of) capitalization. Lists of viruses within a given species are provisional at this point and will likely be amended in the near future.

**Table 7.** ICTV-accepted taxonomy of the order *Articulavirales* (*Negarnaviricota: Polyploviricotina: Insthoviricetes*) as of April 2023

Genus	Species^∗^	Virus (Abbreviation)†
**Family *Amnoonviridae***		
*Tilapinevirus*	*Tilapinevirus tilapiae*	tilapia lake virus (TiLV)
**Family *Orthomyxoviridae***		
*Alphainfluenzavirus*	*Alphainfluenzavirus influenzae*	influenza A virus (FLUAV)
*Betainfluenzavirus*	*Betainfluenzavirus influenzae*	influenza B virus (FLUBV)
*Deltainfluenzavirus*	*Deltainfluenzavirus influenzae*	influenza D virus (FLUDV)
*Gammainfluenzavirus*	*Gammainfluenzavirus influenzae*	influenza C virus (FLUCV)
*Isavirus*	*Isavirus salaris*	infectious salmon anaemia virus (ISAV)
*Mykissvirus*	*Mykissvirus tructae*	rainbow trout orthomyxovirus (RbtOV)
		steelhead trout orthomyxovirus (SttOV1)
*Quaranjavirus*	*Quaranjavirus araguariense*	Araguari virus (ARAV)
	*Quaranjavirus chadense*	Lake Chad virus (LKCV)
	*Quaranjavirus johnstonense*	Johnston Atoll virus (JAV)
	*Quaranjavirus quaranfilense*	Quaranfil virus (QRFV)
	*Quaranjavirus tyulekense*	Tyulek (Tjuloc) virus (TLKV)
	*Quaranjavirus wellfleetense*	Wellfleet Bay virus (WFBV)
*Sardinovirus*	*Sardinovirus pilchardi*	pilchard orthomyxovirus (POMV)
*Thogotovirus*	*Thogotovirus bourbonense*	Bourbon virus (BRBV)
	*Thogotovirus dhoriense*	Dhori virus (DHOV)
	*Thogotovirus josense*	Jos virus (JOSV)
	*Thogotovirus ozense*	Oz virus (OZV)
	*Thogotovirus sinuense*	Sinu virus (SINUV)
	*Thogotovirus thailandense*	Thailand tick thogotovirus (TT-THOV)
	*Thogotovirus thogotoense*	Thogoto virus (THOV)
	*Thogotovirus upoluense*	Upolu virus (UPOV)

Note that viruses are real objects that are assigned to concepts that are called taxa. Species, genera, families, and orders are taxa. Species, genera, families, and orders are taxa. As appropriate, cultural significance may be conveyed in virus names.

^∗^Taxon names are always italicized and always begin with a capital letter.

†Virus names are not italicized and are not capitalized, except if the name or a name component is a proper noun. This column lists the virus names with their correct (lack of) capitalization.

**Table 8.** ICTV-accepted taxonomy of family *Tosoviridae* (*Negarnaviricota*: unassigned) as of April 2023

Genus	Species^∗^	Virus (Abbreviation)†
**Family *Tosoviridae***		
*Fraservirus*	*Fraservirus testudinis*	turtle fraservirus 1 (TFV1)

Note that viruses are real objects that are assigned to concepts that are called taxa. Species, genera, families, and orders are taxa. Species, genera, families, and orders are taxa. As appropriate, cultural significance may be conveyed in virus names.

^∗^Taxon names are always italicized and always begin with a capital letter.

†Virus names are not italicized and are not capitalized, except if the name or a name component is a proper noun. This column lists the virus names with their correct (lack of) capitalization.

## Supplementary Data

Supplementary material 1

Supplementary material 2
