# Supplementary material for: Annual (2023) taxonomic update of RNA-directed RNA polymerase-encoding negative-sense RNA viruses (realm Riboviria: kingdom Orthornavirae: phylum Negarnaviricota)
Source: J Gen Virol. 2023 Aug 25;104(8):001864. doi: 10.1099/jgv.0.001864 (PMC10721048; doi:10.1099/jgv.0.001864)
Supplement: Supplementary material 1 [file jgv-104-1864-s001.pdf]

2023 taxonomy of phylum *Negarnaviricota* (*Riboviria: Orthornavirae*): subphylum *Haploviricotina*

Kuhn JH *et al.* (2023) 2023 taxonomic update of phylum *Negarnaviricota* (*Riboviria: Orthornavirae*), including the large orders *Bunyavirales* and *Mononegavirales*.

Negarnaviricota

Haploviricotina

Polyploviricotina, Unassigned

Chunqiuviricetes

Muvirales

Qinviridae

Yingvirus
- *Yingvirus beihaiense* — Běihǎi sesarmid crab virus 4 (BhSCV4)
- *Yingvirus charybdis* — Wēnzhōu qinvirus-like virus 2 (WzQLV2)
- *Yingvirus hubeiense* — Húběi qinvirus-like virus 1 (HbQLV1)
- *Yingvirus sanxiaense* — Sānxiá qinvirus-like virus 1 (SxQLV1)
- *Yingvirus shaheense* — Shāhé qinvirus-like virus 1 (ShQLV1)
- *Yingvirus wenzhouense* — Wēnzhōu qinvirus-like virus 1 (WzQLV1)
- *Yingvirus wuhanense* — Wǔhàn insect virus 15 (WhIV15)
- *Yingvirus xinzhouense* — Xīnzhōu nematode virus 3 (XzNV3)

Milneviricetes

Serpentovirales

Aspiviridae

Ophiovirus
- *Ophiovirus citri* — citrus psorosis virus (CPsV)
- *Ophiovirus freesiae* — freesia sneak virus (FreSV)
- *Ophiovirus lactucae* — lettuce ring necrosis virus (LRNV)
- *Ophiovirus mirafioriense* — Mirafiori lettuce big-vein virus (MLBVV)
- *Ophiovirus ranunculi* — ranunculus white mottle virus (RWMV)
- *Ophiovirus tulipae* — tulip mild mottle mosaic virus (TMMMV)
- *Ophiovirus vaccinii* — blueberry mosaic associated virus (BlMaV)

Yunchangviricetes

Goujianvirales

Yueviridae

Yuyuevirus
- *Yuyuevirus beihaiense* — Běihǎi sesarmid crab virus 3 (BhSCV3)
- *Yuyuevirus shaheense* — Shāhé yuèvirus-like virus 1 (ShYLV1)

Monjiviricetes

Jingchuvirales

Aliusviridae

Obscuruvirus
- *Obscuruvirus quintum* — Atrato chu-like virus 5 (ACVS)

Ollusvirus
- *Ollusvirus coleopteri* — Húběi coleoptera virus 3 (HbCV3)
- *Ollusvirus culvertonense* — Culverton virus (CVV)
- *Ollusvirus hanchengense* — Hánchéng leafhopper mivirus (HLMV)
- *Ollusvirus hymenopteri* — hymenopteran chu-related virus 123 (HCrV123)
- *Ollusvirus insectii* — hymenopteran chu-related virus 126 (HCrV126)
- *Ollusvirus nomadae* — Nomada lathburiana mononega-like virus (NLmV)
- *Ollusvirus oropsyllae* — Oropsylla silantiewi mononega-like virus 2 (OSmlV2)
- *Ollusvirus rhagoveliae* — Rhagovelia obesa mononega-like virus (ROmV)
- *Ollusvirus scaldisense* — Scaldis River bee virus (SRBV)
- *Ollusvirus shayangense* — Shāyáng fly virus 1 (SyFV1)
- *Ollusvirus taiyuanense* — Tàiyuán leafhopper virus (TYLeV)

Chuviridae

Boscovirus
- *Boscovirus hippoboscidae* — Wǔhàn louse fly virus 7 (WhLFV7)
- *Boscovirus hypoboscidae* — Wǔhàn louse fly virus 6 (WhLFV6)

Chuvirus
- *Chuvirus brunnichi* — Wēnlǐng crustacean virus 14 (WlCV14)
- *Chuvirus cancris* — Wēnzhōu crab virus 2 (WzCV2)

Culicidavirus
- *Culicidavirus culicidae* — Wǔhàn mosquito virus 8 (WhMV8)
- *Culicidavirus culicis* — Culex mosquito virus 5 (CMV5)
- *Culicidavirus imjinense* — Imjin River virus 1 (IjRV1)
- *Culicidavirus quitotaense* — Culex mosquito virus 4 (CMV4)

Demapteravirus
- *Demapteravirus dermapteri* — dermapteran chu-related virus 142 (DCrV142)

Doliuvirus
- *Doliuvirus culisetae* — Mos8Chu0 chuvirus (MoCV)

Mivirus
- *Mivirus amblyommae* — lone star tick chuvirus 1 (LSTCV1)
- *Mivirus belostomatis* — Belostoma flumineum mononega-like virus (BFmlV)
- *Mivirus boleense* — Bólè tick virus 3 (BTV3)
- *Mivirus changpingense* — Chāngpíng tick virus 3 (CpTV3)
- *Mivirus dermacentoris* — Chāngpíng tick virus 3 (CpTV3)
- Tǎchéng tick virus 5 (TcTV5)
- *Mivirus genovaense* — Genoa virus (GeV)
- *Mivirus karukeraense* — Karukera tick virus (KtV)
- *Mivirus rhipicephali* — brown dog tick mivirus 1 (BDTMV1)
- *Mivirus suffolkense* — Suffolk virus (SFKV)
- *Mivirus wuhanense* — Wǔhàn tick virus 2 (WhTV2)

Morsusvirus
- *Morsusvirus argatis* — Tǎchéng tick virus 4 (TcTV4)

Nigecruvirus
- *Nigecruvirus ixodes* — blacklegged tick chuvirus 2 (BTCV2)
- *Nigecruvirus periplanetae* — Periplaneta americana mononega-like virus (PAmlV)

Odonatavirus
- *Odonatavirus dracontis* — odonatan chu-related virus 137 (OCrV137)
- *Odonatavirus fabricii* — Húběi odonate virus 11 (HbOV11)
- *Odonatavirus odontis* — odonatan chu-related virus 136 (OCrV136)

Pediavirus
- *Pediavirus cirripedis* — Běihǎi barnacle virus 9 (BhBV9)

Piscichuvirus
- *Piscichuvirus craterocephali* — hardyhead chuvirus (HhCV)
- *Piscichuvirus franki* — Herr Frank virus 1 (HFrV1)
- *Piscichuvirus lycodontis* — Guǎngdōng red-banded snake chuvirus-like virus (GRSCV)
- *Piscichuvirus sanxiaense* — Sānxiá atyid shrimp virus 4 (SxASC-4)
- *Piscichuvirus wenlingense* — Wēnlǐng fish chu-like virus (WFCV)

Pterovirus
- *Pterovirus chulinense* — hymenopteran chu-related virus OKIAV147 (HCrV147)

Rochuvirus
- *Rochuvirus chalcocoris* — Chalcocoris rutilans mononega-like virus 2 (CRmlV2)

Scarabeuvirus
- *Scarabeuvirus blattae* — Wǔchāng cockroach virus 3 (WcLFV3)
- *Scarabeuvirus dentati* — Húběi chuvirus-like virus 3 (HbCLV3)
- *Scarabeuvirus hubeiense* — Húběi chuvirus-like virus 1 (HbCLV1)
- *Scarabeuvirus lampyris* — Lampyris noctiluca chuvirus-like virus 1 (LNCLV1)
- *Scarabeuvirus lishiense* — Lìshì spider virus 1 (LsSV1)
- *Scarabeuvirus supellae* — Supella longipalpa mononega-like virus 2 (SLmlV2)
- *Scarabeuvirus trichopriae* — Trichopria drosophilae mononega-like virus (TDmlV)

Taceavirus
- *Taceavirus wenlingense* — Wēnlǐng crustacean virus 15 (WlCV15)

Vapochuvirus
- *Vapochuvirus triaeuroidis* — Trialeurodes vaporariorum mononega-like virus 2 (TVmlV2)

Crepuscuviridae

Aqualaruvirus
- *Aqualaruvirus sialis* — megalopteran chu-related virus 119 (MCrV119)

Myriaviridae

Myriavirus
- *Myriavirus myriapedis* — Húběi myriapoda virus 8 (HbMV8)

Natareviridae

Charybdivirus
- *Charybdivirus charybdis* — Wēnzhōu crab virus 3 (WzCV3)

Mononegavirales

Artoviridae

Hexartovirus
- *Hexartovirus cirripedis* — Běihǎi barnacle virus 8 (BhBV8)
- *Hexartovirus lepeophtheiri* — Lepeophtheirus salmonis negative-stranded RNA virus 1 (LsNSRV1)

Peropuvirus
- *Peropuvirus beihaiense* — Běihǎi rhabdo-like virus 1 (BhRLV1)
- *Peropuvirus dentati* — Húběi rhabdo-like virus 8 (HbRLV8)
- *Peropuvirus hubeiense* — Húběi rhabdo-like virus 6 (HbRLV6)
- *Peropuvirus juli* — Húběi rhabdo-like virus 5 (HbRLV5)
- *Peropuvirus lignarii* — Běihǎi rhabdo-like virus 2 (BhRLV2)
- *Peropuvirus melongenae* — Solanum melongena rhabdo-like virus (SmRLV)
- *Peropuvirus pteromali* — Pteromalus puparum negative-strand RNA virus 1 (PpNSRV1)

Bornaviridae

Carbovirus
- *Carbovirus queenslandense* — jungle carpet python virus (JCPV)
- *Carbovirus tapeti* — southwest carpet python virus (SWCPV)

Cultervirus
- *Cultervirus hemicultri* — Wǔhàn sharpbelly bornavirus (WhSBV)

Orthobornavirus
- *Orthobornavirus alphapsittaciforme* — parrot bornavirus 1 (PaBV1), parrot bornavirus 2 (PaBV2), parrot bornavirus 3 (PaBV3), parrot bornavirus 4 (PaBV4), parrot bornavirus 7 (PaBV7)
- *Orthobornavirus avisaquaticae* — aquatic bird bornavirus 1 (ABBV1), aquatic bird bornavirus 2 (ABBV2)
- *Orthobornavirus betapsittaciforme* — parrot bornavirus 5 (PaBV5)
- *Orthobornavirus bornaense* — Borna disease virus 1 (BoDV1), Borna disease virus 2 (BoDV2)
- *Orthobornavirus caenophidiae* — Caribbean watersnake bornavirus (CWBV), Mexican black-tailed rattlesnake bornavirus (MRBV)
- *Orthobornavirus elapsoideae* — Loveridge's garter snake virus 1 (LGSV1)
- *Orthobornavirus estrildidae* — estrildid finch bornavirus 1 (EsBV1)
- *Orthobornavirus sciuri* — variegated squirrel bornavirus 1 (VSBV1)
- *Orthobornavirus serini* — canary bornavirus 1 (CnBV1), canary bornavirus 2 (CnBV2), canary bornavirus 3 (CnBV3)

Filoviridae

Cuevavirus
- *Cuevavirus lloviuense* — Lloviu virus (LLOV)

Dianlovirus
- *Dianlovirus menglaense* — Mènglà virus (MLAV)

Oblavirus
- *Oblavirus percae* — Oberland virus (OBLV)

Orthoebolavirus
- *Orthoebolavirus bombaliense* — Bombali virus (BOMV)
- *Orthoebolavirus bundibugyoense* — Bundibugyo virus (BDBV)
- *Orthoebolavirus restonense* — Reston virus (RESTV)
- *Orthoebolavirus sudanense* — Sudan virus (SUDV)
- *Orthoebolavirus taiense* — Taï Forest virus (TAFV)
- *Orthoebolavirus zairense* — Ebola virus (EBOV)

Orthomarburgvirus
- *Orthomarburgvirus marburgense* — Marburg virus (MARV), Ravn virus (RAVV)

Striavirus
- *Striavirus antennarii* — Xīlǎng virus (XILV)

Tapjovirus
- *Tapjovirus bothropis* — Tapajós virus (TAPV)

Thamnovirus
- *Thamnovirus kanderense* — Kander virus (KNDV)
- *Thamnovirus percae* — Fiwi virus (FIWIV)
- *Thamnovirus thamnaconi* — Huángjiāo virus (HUJV)

Lispiviridae

Acridvirus
- *Acridvirus hangzhouense* — Hángzhōu acrida cinerea lispivirus 1 (HzACLV1)

Aleyavirus
- *Aleyavirus fuyangense* — Bemisia tabaci arlivirus 1 (BtAV1)

Aleybvirus
- *Aleybvirus fuyangense* — Bemisia tabaci arlivirus 2 (BtAV2)

Anicalvirus
- *Anicalvirus hangzhouense* — Anisopteromalus calandrae negative-strand RNA virus 2 (AcNSRV2)

Anidravirus
- *Anidravirus hangzhouense* — Anisopteromalus calandrae negative-strand RNA virus 1 (AcNSRV1)

Aranavirus
- *Aranavirus guiyangense* — Guìyáng lispivirus 1 (GyLV1)

Aranbvirus
- *Aranbvirus guiyangense* — Guìyáng lispivirus 2 (GyLV2)

Arlivirus
- *Arlivirus arachnae* — Lìshì spider virus 2 (LsSV2)
- *Arlivirus hangzhouense* — Hángzhōu Scotinophara lurida lispivirus 1 (HzSLLV1)
- *Arlivirus ningboense* — Níngbō tick virus 1 (NbuSBV1)

Avesvirus
- *Avesvirus sinense* — Arlivirus sp. virus (ALV)

Copasivirus
- *Copasivirus ixindoense* — isopteran arli-related virus OKIAV103 (IARV103)
- *Copasivirus manlyvaleense* — Jimsystemes virus (JIMV)

Cybintervirus
- *Cybintervirus niederense* — coleopteran arli-related virus OKIAV107 (CARV107)

Damravirus
- *Damravirus dentatis* — Húběi odonate virus 10 (HbOV10)
- *Damravirus fushunense* — Fǔshùn Ischnura senegalensis lispivirus 1 (FsISLV1)

Ganiavirus
- *Ganiavirus tachengense* — Tǎchéng tick virus 6 (TcTV6)

Hemipivirus
- *Hemipivirus scuti* — Hángzhōu Eysarcoris guttigerus lispivirus 1 (HzEGLV1)
- *Hemipivirus veri* — Hángzhōu Cletus punctiger lispivirus 1 (HzCPLV1)

Leocovirus
- *Leocovirus coleopteris* — Húběi rhabdo-like virus 3 (HbRLV3)

Nematovirus
- *Nematovirus wuchangense* — Wǔchāng romanomermis nematode virus 2 (WcRNV2)

Phelinovirus
- *Phelinovirus aphidis* — hymenopteran arli-related virus OKIAV98 (HARV98)

Rivapovirus
- *Rivapovirus aleyrodidae* — hemipteran arli-related virus OKIAV94 (HARV94)

Sanstrivirus
- *Sanstrivirus gerridis* — Sānxiá water strider virus 4 (SxWSV4)

Stylovirus
- *Stylovirus niederense* — strepsipteran arli-related virus OKIAV104 (SARV104)

Supelovirus
- *Supelovirus thailandense* — blattodean arli-related virus OKIAV102 (BARV102)

Synelinevirus
- *Synelinevirus bonnense* — hymenopteran arli-related virus OKIAV98 (HARV98)
- *Synelinevirus paranaense* — Linepithema humile rhabdo-like virus 1 (LhuRLV1)

Usmuvirus
- *Usmuvirus newyorkense* — Amsterdam virus (AMSV)

Xenophyvirus
- *Xenophyvirus mathesonense* — hemipteran arli-related virus OKIAV95 (HARV95)

Mymonaviridae

Auricularimonavirus
- *Auricularimonavirus armillariae* — Armillaria mellea negative strand RNA virus 2 (AmNSRV2)
- *Auricularimonavirus auriculariae* — Auricularia heimuer negative-stranded RNA virus 1 (AhNRSV1)
- *Auricularimonavirus bondarzewiae* — Bondarzewia berkeleyi negative-strand RNA virus 1 (BbNSRV1)

Botrytimonavirus
- *Botrytimonavirus alphabotrytidis* — Botrytis cinerea negative-stranded RNA virus 5 (BcNSRV5)
- Sclerotinia sclerotiorum negative-stranded RNA virus 10 (SsNSRV10)
- *Botrytimonavirus botrytidis* — Botrytis cinerea negative-stranded RNA virus 7 (BcNSRV7)
- Sclerotinia sclerotiorum negative-stranded RNA virus 11 (SsNSRV11)
- *Botrytimonavirus glycinis* — soybean leaf-associated negative-stranded RNA virus 3 (SLaNSRV3)
- *Botrytimonavirus sclerotiniae* — Sclerotinia sclerotiorum negative-stranded RNA virus 2 (SsNSRV2)
- Sclerotinia sclerotiorum negative-stranded RNA virus 2-A (SsNSRV2A)
- Sclerotinia sclerotiorum negative-stranded RNA virus 4 (SsNSRV4)
- Sclerotinia sclerotiorum negative-stranded RNA virus 4-A (SsNSRV4A)

Hubramonavirus
- *Hubramonavirus fusarii* — Fusarium oxysporum mymonavirus 1 (FoMyV1)
- *Hubramonavirus golovinomycesae* — Golovinomyces cichoracearum negative-stranded RNA virus (GcNSRV1)
- *Hubramonavirus hubeiense* — Húběi rhabdo-like virus 4 (HbRLV4)
- *Hubramonavirus terrae* — H2BulkLitter1223 virus (H2BLV)
- *Hubramonavirus vitis* — grapevine-associated mononega-like virus 3 (GaMLV3)

Lentimonavirus
- *Lentimonavirus armillariae* — Armillaria mellea negative-stranded RNA virus 1 (AmNSRV1)
- *Lentimonavirus lentinulae* — Lentinula edodes negative-stranded RNA virus 1 (LeNSRV1)

Penicillimonavirus
- *Penicillimonavirus alphaerysiphe* — Erysiphe necator associated negative-stranded RNA virus 2 (EnaNSRV2)
- *Penicillimonavirus alphapenicillii* — Penicillium adametzioides negative-stranded RNA virus 1 (PaNsRV1)
- *Penicillimonavirus alphaplasmoparae* — Plasmopara viticola lesion associated mononegaambi virus 1 (PvaMV1)
- *Penicillimonavirus betaerysiphe* — Erysiphe necator associated negative-stranded RNA virus 5 (EnaNSRV5)
- *Penicillimonavirus betapenicillii* — Penicillium glabrum negative-stranded RNA virus 1 (PgRV1)
- *Penicillimonavirus betaplasmoparae* — Plasmopara viticola lesion associated mononegaambi virus 2 (PvaMV2)
- Plasmopara viticola lesion associated mononegaambi virus 4 (PvaMV4)
- *Penicillimonavirus deltaplasmoparae* — Plasmopara viticola lesion associated mononegaambi virus 5 (PvaMV5)
- *Penicillimonavirus epsilonplasmoparae* — Plasmopara viticola lesion associated mononegaambi virus 6 (PvaMV6)
- *Penicillimonavirus etaplasmoparae* — Plasmopara viticola lesion associated mononegaambi virus 9 (PvaMV9)
- *Penicillimonavirus gammaerysiphe* — Erysiphe necator associated negative-stranded RNA virus 6 (EnaNSRV6)
- *Penicillimonavirus gammaplasmoparae* — Plasmopara viticola lesion associated mononegaambi virus 3 (PvaMV3)
- *Penicillimonavirus kilnbarnense* — Kiln Barn virus (KBV)
- *Penicillimonavirus magnaporthe* — Magnaporthe oryzae mononegaambi virus 1 (MoMV1)
- *Penicillimonavirus zetaplasmoparae* — Plasmopara viticola lesion associated mononegaambi virus 7 (PvaMV7)

Phyllomonavirus
- *Phyllomonavirus gysingense* — Gysinge virus (GYSV)
- *Phyllomonavirus phyllosphere* — soybean leaf-associated negative-stranded RNA virus 4 (SLaNSRV4)

Plasmopamonavirus
- *Plasmopamonavirus erysiphe* — Erysiphe necator associated negative-stranded RNA virus 23 (EnaNSRV23)
- *Plasmopamonavirus plasmoparae* — Plasmopara viticola lesion associated mononegaambi virus 8 (PvaMV8)

Rhizomonavirus
- *Rhizomonavirus mali* — apple virus B (APVB)

Sclerotimonavirus
- *Sclerotimonavirus alphabotrytidis* — Botrytis cinerea negative-stranded RNA virus 3 (BcNSRV3)
- Sclerotinia sclerotiorum negative-stranded RNA virus 9 (SsNSRV9)
- *Sclerotimonavirus alphasclerotiniae* — Sclerotinia homoeocarpa TSA contig 1 (ShTSA-1)
- *Sclerotimonavirus alphashiwa* — Sclerotinia homoeocarpa TSA contig 2 (ShTSA-2)
- *Sclerotimonavirus alphaplasmoparae* — Plasmopara viticola lesion associated mymonavirus 1 (PvaMM1)
- *Sclerotimonavirus alternariae* — Alternaria tenuissima negative-stranded RNA virus 1 (AtNsRV1)
- *Sclerotimonavirus asiafusarii* — Fusarium graminearum negative-stranded RNA virus 1 (FgNSRV1)
- *Sclerotimonavirus betabotrytidis* — Botrytis cinerea negative-stranded RNA virus 4 (BcNSRV4)
- *Sclerotimonavirus betaclarireedia* — Sclerotinia homoeocarpa TSA contig 3 (ShTSA-3)
- *Sclerotimonavirus betaplasmoparae* — Plasmopara viticola lesion associated mononega virus 2 (PvaMV2)
- *Sclerotimonavirus botrytidis* — Botrytis cinerea mymonavirus 1 (BcMyV1)
- Sclerotinia sclerotiorum negative-stranded RNA virus 6 (SsNSRV6)
- *Sclerotimonavirus cryphonectriae* — Cryphonectria parasitica sclerotimonavirus 1 (CpSV1)
- *Sclerotimonavirus fusarii* — Fusarium graminearum negative-stranded RNA virus 2 (FgNSRV2)
- soybean leaf-associated negative-stranded RNA virus 2 (SLaNSRV2)
- *Sclerotimonavirus illinoisense* — soybean leaf-associated negative-stranded RNA virus 1 (SLaNSRV1)
- *Sclerotimonavirus penicillii* — Penicillium cainsense negative-stranded RNA virus 1 (PcNsRV1)
- *Sclerotimonavirus sclerotiniae* — Sclerotinia sclerotiorum negative-stranded RNA virus 1 (SsNSRV1)
- Sclerotinia sclerotiorum negative-stranded RNA virus 1-A (SsNSRV1A)
- Sclerotinia sclerotiorum negative-stranded RNA virus 3 (SsNSRV3)
- Sclerotinia sclerotiorum negative-stranded RNA virus 5 (SsNSRV5)
- *Sclerotimonavirus terrae* — H4BulkLitter234 virus (H4BLV)
- *Sclerotimonavirus xinjiangense* — Xīnjiāng mymona-like virus 2 (XjMLV2)
- *Sclerotimonavirus yunnanense* — Yúnnán mymona-like virus 1 (YnMLV1)

Nyamiviridae

Berhavirus
- *Berhavirus beihaiense* — Běihǎi rhabdo-like virus 4 (BhRLV4)
- *Berhavirus radialis* — Běihǎi rhabdo-like virus 5 (BhRLV5)
- *Berhavirus sipunculi* — Běihǎi rhabdo-like virus 3 (BhRLV3)

Crustavirus
- *Crustavirus beihaiense* — Běihǎi rhabdo-like virus 6 (BhRLV6)
- *Crustavirus wenlingense* — Wēnlǐng crustacean virus 12 (WlCV12)
- *Crustavirus wenzhouense* — Wēnzhōu crab virus 1 (WzCV1)

Formivirus
- *Formivirus angliae* — Formica fusca virus 1 (FfusV1)
- *Formivirus chalybii* — hymenopteran orino-related virus OKIAV87 (HORV87)
- *Formivirus finnoniae* — Formica exsecta virus 4 (FeV4)
- *Formivirus gorytis* — hymenopteran orino-related virus OKIAV85 (HORV85)
- *Formivirus pollinis* — Xiāngshān nyami-like virus (XNyV)
- *Formivirus solenopsi* — Solenopsis invicta virus 15 (SolINV15)

Nyavirus
- *Nyavirus argatis* — Sekira virus (SEKRV)
- *Nyavirus midwayense* — Midway virus (MIDWV)
- *Nyavirus nyamaniniense* — Nyamanini virus (NYMV)
- *Nyavirus sanjacintoense* — San Jacinto virus (SJCV)
- *Nyavirus sierranevadaense* — Sierra Nevada virus (SNVV)
- *Nyavirus somateriae* — Jeremy Point nyavirus (JPNV)

Orinovirus
- *Orinovirus pasiphilae* — Orinoco virus (ONCV)
- *Orinovirus sanyae* — Sanya nyamivirus 1 (SaNyV1)

Socyvirus
- *Socyvirus heteroderae* — soybean cyst nematode virus 1 (SbCNV1)

Tapwovirus
- *Tapwovirus cesti* — Wēnzhōu tapeworm virus 1 (WzTWV1)

Paramyxoviridae

Avulavirinae

Metaavulavirus
- *Metaavulavirus delawarense* — avian paramyxovirus 8 (APMV8)
- *Metaavulavirus falklandense* — avian paramyxovirus 10 (APMV10)
- *Metaavulavirus gallinense* — avian paramyxovirus 11 (APMV11)
- *Metaavulavirus hongkongense* — avian paramyxovirus 6 (APMV6)
- *Metaavulavirus japanense* — avian paramyxovirus 14 (APMV14)
- *Metaavulavirus kazakhstanense* — avian paramyxovirus 20 (APMV20)
- *Metaavulavirus kunitachiense* — avian paramyxovirus 5 (APMV5)
- *Metaavulavirus peiense* — avian paramyxovirus 15 (APMV15)
- *Metaavulavirus taiwanense* — avian paramyxovirus 22 (APMV22)
- *Metaavulavirus tennesseense* — avian paramyxovirus 7 (APMV7)
- *Metaavulavirus yucaipaense* — avian paramyxovirus 2 (APMV2)

Orthoavulavirus
- *Orthoavulavirus borisense* — Antarctic penguin virus A (APVA)
- *Orthoavulavirus italiense* — avian paramyxovirus 12 (APMV12)
- *Orthoavulavirus japanense* — avian paramyxovirus 1 (APMV1)[1]
- *Orthoavulavirus javaense* — avian paramyxovirus 1 (APMV1)[1]
- *Orthoavulavirus kopaicense* — Antarctic penguin virus B (APVB)
- *Orthoavulavirus koreaense* — avian paramyxovirus 21 (APMV21)
- *Orthoavulavirus newyorkense* — avian paramyxovirus 9 (APMV9)
- *Orthoavulavirus oneillense* — Antarctic penguin virus C (APVC)
- *Orthoavulavirus upoense* — avian paramyxovirus 16 (APMV16)

Paraavulavirus
- *Paraavulavirus hongkongense* — avian paramyxovirus 4 (APMV4)
- *Paraavulavirus wisconsinense* — avian paramyxovirus 3 (APMV3)

Metaparamyxovirinae

Synodonvirus
- *Synodonvirus synodi* — Wēnlǐng triplecross lizardfish paramyxovirus (WTLPV)

Orthoparamyxovirinae

Aquaparamyxovirus
- *Aquaparamyxovirus oregonense* — Pacific salmon paramyxovirus (PSPV)
- *Aquaparamyxovirus salmonis* — Atlantic salmon paramyxovirus (AsaPV)

Ferlavirus
- *Ferlavirus reptilis* — fer-de-lance virus (FDLV)

Henipavirus
- *Henipavirus cedarense* — Cedar virus (CedV)
- *Henipavirus ghanaense* — Ghana virus (GhV)
- *Henipavirus hendraense* — Hendra virus (HeV)
- *Henipavirus mojiangense* — Mòjiāng virus (MojV)
- *Henipavirus nipahense* — Nipah virus (NiV)

Jeilongvirus
- *Jeilongvirus anhuiense* — Miniopterus schreibersii paramyxovirus (MisPV)
- *Jeilongvirus apodemi* — rodent paramyxovirus (RoPV)
- *Jeilongvirus beilongi* — Beilong virus (BeiV)
- *Jeilongvirus comorosense* — bat paramyxovirus 16797 (BatPV1)
- *Jeilongvirus erinacei* — belerina virus (BelV)
- *Jeilongvirus felis* — feline paramyxovirus (FPaV)
- *Jeilongvirus lophuromys* — Mount Mabu Lophuromys virus 1 (MMLV1)
- *Jeilongvirus mabuense* — Mount Mabu Lophuromys virus 2 (MMLV2)
- *Jeilongvirus madagascarense* — bat paramyxovirus 17770 (BatPV2)
- *Jeilongvirus miniopteri* — Shaan virus (ShaV)
- *Jeilongvirus murinae* — bat paramyxovirus (BatPV3)
- *Jeilongvirus myodesis* — Pohorje Myodes paramyxovirus 1 (PMPV1)
- *Jeilongvirus queenslandense* — J virus (JV)
- *Jeilongvirus rungweense* — ruloma virus (RulV)
- *Jeilongvirus tailamense* — Tailam virus (TlmV)

Morbillivirus
- *Morbillivirus canis* — canine distemper virus (CDV)
- *Morbillivirus caprinae* — peste-des-petits-ruminants virus (PPRV)
- *Morbillivirus ceti* — cetacean morbillivirus (CeMV)
- *Morbillivirus felis* — feline morbillivirus (FeMV)
- *Morbillivirus hominis* — measles virus (MeV)
- *Morbillivirus pecoris* — rinderpest virus (RPV)
- *Morbillivirus phocae* — phocine distemper virus (PDV)

Narmovirus
- *Narmovirus mossmanense* — Mossman virus (MossV)
- *Narmovirus myodesis* — bank vole virus 1 (BaV1)
- *Narmovirus nariva* — Nariva virus (NarV)
- *Narmovirus tupaiae* — Tupaia paramyxovirus (TupV)

Respirovirus
- *Respirovirus bovis* — bovine parainfluenza virus 3 (BPIV3)
- *Respirovirus caprae* — caprine parainfluenza virus 3 (CPIV3)
- *Respirovirus laryngotracheitidis* — human parainfluenza virus 1 (HPIV1)
- *Respirovirus muris* — Sendai virus (SeV)
- *Respirovirus pneumoniae* — human parainfluenza virus 3 (HPIV3)
- *Respirovirus rotufae* — giant squirrel virus (GSqV)
- *Respirovirus suis* — porcine parainfluenza virus 1 (PPIV1)

Salemvirus
- *Salemvirus salemense* — Salem virus (SaV)

Rubulavirinae

Orthorubulavirus
- *Orthorubulavirus alstonvillense* — Alston virus (AlsV)
- *Orthorubulavirus hominis* — human parainfluenza virus 4a (HPIV4a), human parainfluenza virus 4b (HPIV4b)
- *Orthorubulavirus laryngotracheitidis* — human parainfluenza virus 2 (HPIV2)
- *Orthorubulavirus mammalis* — parainfluenza virus 5 (PIV5)
- *Orthorubulavirus mapuerae* — Mapuera virus (MapV)
- *Orthorubulavirus parotitidis* — mumps virus (MuV)
- *Orthorubulavirus simiae* — simian virus 41 (SV41)
- *Orthorubulavirus suis* — La Piedad Michoacán Mexico virus (LPMV)

Pararubulavirus
- *Pararubulavirus accraense* — Achimota virus 2 (AchPV2)
- *Pararubulavirus achimotaense* — Achimota virus 1 (AchPV1)
- *Pararubulavirus cantonense* — Tuhoko virus 2 (ThkPV2)
- *Pararubulavirus guangdongense* — Tuhoko virus 1 (ThkPV1)
- *Pararubulavirus herveyense* — Hervey virus (HerV)
- *Pararubulavirus hongkongi* — Tuhoko virus 3 (ThkPV3)
- *Pararubulavirus menangleense* — Menangle virus (MenPV)
- *Pararubulavirus sosugaense* — Sosuga virus (SOSV)
- *Pararubulavirus teviotense* — Teviot virus (TevPV)
- *Pararubulavirus tiomanense* — Tioman virus (TioPV)

Unassigned

Cynoglossusvirus
- *Cynoglossusvirus cynoglossi* — Wēnlǐng tonguesole paramyxovirus (WTSPV)

Hoplichthysvirus
- *Hoplichthysvirus hoplichthysis* — Wēnlǐng hoplichthys paramyxovirus (WHPV)

Scoliodonvirus
- *Scoliodonvirus scoliodontis* — Wēnzhōu pacific spadenose shark paramyxovirus (WPSSPV)

Pneumoviridae

Metapneumovirus
- *Metapneumovirus avis* — avian metapneumovirus (AMPV)
- *Metapneumovirus hominis* — human metapneumovirus (HMPV)

Orthopneumovirus
- *Orthopneumovirus bovis* — bovine respiratory syncytial virus (BRSV)
- *Orthopneumovirus hominis* — human respiratory syncytial virus (HRSV)
- *Orthopneumovirus muris* — murine pneumonia virus (MPV)

Rhabdoviridae

Alpharhabdovirinae

Almendravirus
- *Almendravirus almendras* — Puerto Almendras virus (PTAMV)
- *Almendravirus arboretum* — Arboretum virus (ABTV)
- *Almendravirus balsa* — Balsa virus (BALV)
- *Almendravirus chico* — Rio Chico virus (RCHV)
- *Almendravirus cootbay* — Coot Bay virus (CBV)
- *Almendravirus menghai* — Menghai virus (MRV)
- *Almendravirus xianshan* — Xiangshan rhabdo-like virus 1 (XsRLV1)

Alphanemrhavirus
- *Alphanemrhavirus bangkok* — Rattus tanezumi rhabdovirus 1 (RtaRV1)
- *Alphanemrhavirus sodak* — Sodak rhabdovirus 1 (SDRV1)
- *Alphanemrhavirus xingshan* — Xingshan nematode virus 4 (XsNV4)
- *Alphanemrhavirus xinzhou* — Xinzhou nematode virus 4 (XzNV4)

Alphapaprhavirus
- *Alphapaprhavirus hubei* — Hubei lepidoptera virus 2 (HbLV2)
- *Alphapaprhavirus parrarge* — Pararge aegeria rhabdovirus (PaeRV)

Alpharicinrhavirus
- *Alpharicinrhavirus blanchseco* — Blanchseco virus (BCOV)
- *Alpharicinrhavirus bole* — Bole tick virus 2 (BTV2)
- *Alpharicinrhavirus hubei* — Hubei tick rhabdovirus 1 (HbTRV1)
- *Alpharicinrhavirus wuhan* — Wuhan tick virus 1 (WhTV1)

Amplylivirus
- *Amplylivirus cineres* — frog lyssa-like virus 1 (FLLV1)

Arurhavirus
- *Arurhavirus aruac* — Aruac virus (ARUV)
- *Arurhavirus inhangapi* — Inhangapi virus (INHV)
- *Arurhavirus santabarbara* — Santa Barbara virus (SBAV)
- *Arurhavirus xiburema* — Xiburema virus (XIBV)

Barhavirus
- *Barhavirus bahia* — Bahia Grande virus (BGV)
- *Barhavirus harlingen* — Harlingen virus (HARV)
- *Barhavirus muir* — Muir Springs virus (MSV)

Caligrhavirus
- *Caligrhavirus caligus* — Caligus rogercresseyi rhabdovirus (CRogRV)
- *Caligrhavirus lepeophtheirus* — Lepeophtheirus salmonis rhabdovirus 127 (LSalRV127)
- *Caligrhavirus salmonlouse* — Lepeophtheirus salmonis rhabdovirus 9 (LSalRV9)

Cetarhavirus
- *Cetarhavirus lagenorhynchus* — dolphin rhabdovirus (DRV)
- *Cetarhavirus phocoena* — harbour porpoise rhabdovirus (HPRV)

Curiovirus
- *Curiovirus curionopolis* — Curionopolis virus (CURV)
- *Curiovirus iriri* — Iriri virus (IRIRV)
- *Curiovirus itacaiunas* — Itacaiunas virus (ITAV)
- *Curiovirus rochambeau* — Rochambeau virus (RBUV)

Ephemerovirus
- *Ephemerovirus adelaide* — Adelaide River virus (ARV)
- *Ephemerovirus berrimah* — Berrimah virus (BRMV)
- *Ephemerovirus febris* — bovine ephemeral fever virus (BEFV)
- *Ephemerovirus guangdong* — porcine ephemerovirus 2 (PoEV2)
- *Ephemerovirus hayes* — Hayes Yard virus (HYV)
- *Ephemerovirus henan* — porcine ephemerovirus 1 (PoEV1)
- *Ephemerovirus kent* — New Kent County virus (NKCV)
- *Ephemerovirus kimberley* — Kimberley virus (KIMV)
- Malakal virus (MALV)
- *Ephemerovirus koolpinyah* — Koolpinyah virus (KOOLV)
- *Ephemerovirus kotonkan* — kotonkan virus (KOTV)
- *Ephemerovirus obodhiang* — Obodhiang virus (OBOV)
- *Ephemerovirus puchong* — Puchong virus (PUCV)
- *Ephemerovirus yata* — Yata virus (YATV)

Hapavirus
- *Hapavirus bangoran* — Bangoran virus (BGNV)
- *Hapavirus flanders* — Flanders virus (FLAV)
- *Hapavirus gray lodge* — Gray Lodge virus (GLOV)
- *Hapavirus hartpark* — Hart Park virus (HPV)
- *Hapavirus holmes* — Holmes Jungle virus (HOJV)
- *Hapavirus joinjakaka* — Joinjakaka virus (JOIV)
- *Hapavirus kamese* — Kamese virus (KAMV)
- *Hapavirus lajoya* — La Joya virus (LJV)
- *Hapavirus landjia* — Landjia virus (LANV = LJAV)
- *Hapavirus manitoba* — Manitoba virus (MANV = MNTBV)
- *Hapavirus marco* — Marco virus (MCOV)
- *Hapavirus mosqueiro* — Mosqueiro virus (MQOV)
- *Hapavirus mossuril* — Mossuril virus (MOSV)
- *Hapavirus ngaingan* — Ngaingan virus (NGAV)
- *Hapavirus ord* — Ord River virus (ORV)
- *Hapavirus parry* — Parry Creek virus (PCV)
- *Hapavirus porton* — Porton virus (PORV)
- *Hapavirus wongabel* — Wongabel virus (WONV)

Ledantevirus
- *Ledantevirus barur* — Barur virus (BARV)
- *Ledantevirus bughendera* — Bughendera virus (BUGV)
- *Ledantevirus elgon* — Mount Elgon bat virus (MEBV)
- *Ledantevirus fikirini* — Fikirini virus (FKRV)
- *Ledantevirus fukuoka* — Fukuoka virus (FUKV)
- *Ledantevirus kanyawara* — Kanyawara virus (KYAV)
- *Ledantevirus kern* — Kern Canyon virus (KCV)
- *Ledantevirus keuraliba* — Keuraliba virus (KEUV)
- *Ledantevirus kolente* — Kolente virus (KOLEV)
- *Ledantevirus kumasi* — Kumasi rhabdovirus (KRV)
- *Ledantevirus ledantec* — Le Dantec virus (LDV)
- *Ledantevirus longquan* — Longquan Niviventer coninga ledantevirus 1 (LNcoLV1)
- *Ledantevirus nishimuro* — Nishimuro virus (NISV)
- *Ledantevirus nkolbisson* — Nkolbisson virus (NKOV)
- *Ledantevirus oita* — Oita virus (OITAV)
- *Ledantevirus taiyi* — Taiyi bat virus (TYBV)
- *Ledantevirus vaprio* — Vaprio virus (VAPV)
- *Ledantevirus wenzhou* — Wenzhou Rhinolophus pusillus ledantevirus 1 (WRpuLV1)
- *Ledantevirus wuhan* — Wuhan louse fly virus 5 (WLFV5)
- *Ledantevirus yongjia* — Yongjia tick virus 2 (YJTV2)

Lostrhavirus
- *Lostrhavirus hyalomma* — Hyalomma tick rhabdovirus (XjTRV)
- *Lostrhavirus lonestar* — lone star tick rhabdovirus (LSTRV)

Lyssavirus
- *Lyssavirus aravan* — Aravan virus (ARAV)
- *Lyssavirus australis* — Australian bat lyssavirus (ABLV)
- *Lyssavirus bokeloh* — Bokeloh bat lyssavirus (BBLV)
- *Lyssavirus caucasicus* — West Caucasian bat virus (WCBV)
- *Lyssavirus duvenhage* — Duvenhage virus (DUVV)
- *Lyssavirus formosa* — Taiwan bat lyssavirus (TWBLV)
- *Lyssavirus gannoruwa* — Gannoruwa bat lyssavirus (GBLV)
- *Lyssavirus hamburg* — European bat lyssavirus 1 (EBLV1)
- *Lyssavirus helsinki* — European bat lyssavirus 2 (EBLV2)
- *Lyssavirus ikoma* — Ikoma lyssavirus (IKOV)
- *Lyssavirus irkut* — Irkut virus (IRKV)
- *Lyssavirus khujand* — Khujand virus (KHUV)
- *Lyssavirus lagos* — Lagos bat virus (LBV)
- *Lyssavirus lleida* — Lleida bat lyssavirus (LLEBV)
- *Lyssavirus mokola* — Mokola virus (MOKV)
- *Lyssavirus rabies* — rabies virus (RABV)
- *Lyssavirus shimoni* — Shimoni bat virus (SHIBV)

Merhavirus
- *Merhavirus merida* — Merida virus (MERDV)
- *Merhavirus tritaeniorhynchus* — Culex tritaeniorhynchus rhabdovirus (CTRV)

Mousrhavirus
- *Mousrhavirus moussa* — Moussa virus (MOUV)

Ohlsrhavirus
- *Ohlsrhavirus angeles* — Culex rhabdo-like virus Los Angeles (CRLVLA)
- *Ohlsrhavirus culex* — Culex rhabdo-like virus (CRLV)
- *Ohlsrhavirus lobeira* — Lobeira virus (LOBV)
- *Ohlsrhavirus northcreek* — North Creek virus (NORCV)
- *Ohlsrhavirus ohlsdorf* — Ohlsdorf virus (OHLDV)
- *Ohlsrhavirus pseudovishnui* — Culex pseudovishnui rhabdo-like virus (CpRLV)
- *Ohlsrhavirus riverside* — Riverside virus (RISV)
- *Ohlsrhavirus tongilchon* — Tongilchon virus 1 (TCHV1)

Perhabdovirus
- *Perhabdovirus anguilla* — eel virus American (EVA), eel virus European X (EVEX)
- *Perhabdovirus lerman* — Lerman virus (LeRV)
- *Perhabdovirus perca* — perch rhabdovirus (PRV)
- *Perhabdovirus trutta* — lake trout rhabdovirus (LTRV)

Replylivirus
- *Replylivirus ollugus* — anole lyssa-like virus 1 (ALLV1)

Sawgrhavirus
- *Sawgrhavirus connecticut* — Connecticut virus (CNTV)
- *Sawgrhavirus longisland* — Long Island tick rhabdovirus (LITRV)
- *Sawgrhavirus minto* — New Minto virus (NMV)
- *Sawgrhavirus sawgrass* — Sawgrass virus (SAWV)

Scophrhavirus
- *Scophrhavirus chanodychthys* — Wuhan redfin culter dimarhabdovirus (WhRFCRV)
- *Scophrhavirus maximus* — Scophthalmus maximus rhabdovirus (SMRV)

Sigmavirus
- *Sigmavirus affinis* — Drosophila affinis sigmavirus (DAffSV)
- *Sigmavirus ananassae* — Drosophila ananassae sigmavirus (DAnaSV)
- *Sigmavirus capitata* — Ceratitis capitata sigmavirus (CCapSV)
- *Sigmavirus domestica* — Wuhan fly virus 2 (WhFV2)
- *Sigmavirus hippoboscid* — Wuhan louse fly virus 9 (WhLFV9)
- *Sigmavirus hubei* — Hubei diptera virus 9 (HbDV9)
- *Sigmavirus immigrans* — Drosophila immigrans sigmavirus (DImmSV)
- *Sigmavirus lousefly* — Wuhan louse fly virus 10 (WhLFV10)
- *Sigmavirus melanogaster* — Drosophila melanogaster sigmavirus (DMelSV)
- *Sigmavirus muscina* — Muscina stabulans sigmavirus (MStaSV)
- *Sigmavirus myga* — Hubei diptera virus 10 (HbDV10)
- *Sigmavirus obscura* — Drosophila obscura sigmavirus (DObsSV)
- *Sigmavirus shayang* — Shayang fly virus 2 (SyFV2)
- *Sigmavirus sturtevanti* — Drosophila sturtevanti sigmavirus (DStuSV)
- *Sigmavirus tristis* — Drosophila tristis sigmavirus (DTriSV)
- *Sigmavirus tuta* — Aksy-Durug Melophagus sigmavirus (ADMSV)
- *Sigmavirus wuhan* — Wuhan house fly virus 1 (WhHFV1)
- *Sigmavirus yxing* — Hubei dimarhabdovirus 1 (HbDRV1)
- *Sigmavirus yushu* — Yushu rhabdovirus (YsRV)
- *Sigmavirus aphid* — Apis rhabdovirus 3 (ApRV3)

Siniperhavirus
- *Siniperhavirus chuatsi* — Chinese rice-field eel rhabdovirus (CrERV), hybrid snakehead rhabdovirus (HSHRV), Siniperca chuatsi rhabdovirus (SCRV), Micropterus salmoides rhabdovirus (MSRV)
- *Siniperhavirus zoarces* — eelpout rhabdovirus (EPRV)

Sprivivirus
- *Sprivivirus cyprinus* — spring viremia of carp virus (SVCV)
- *Sprivivirus esox* — grass carp rhabdovirus (GrCRV), pike fry rhabdovirus (PFRV), tench rhabdovirus (TenRV)

Sripuvirus
- *Sripuvirus almpiwar* — Almpiwar virus (ALMV)
- *Sripuvirus chaco* — Chaco virus (CHOV)
- *Sripuvirus charleville* — Charleville virus (CHVV)
- *Sripuvirus cuiaba* — Cuiabá virus (CUIV)
- *Sripuvirus hainan* — Hainan black-spectacled toad rhabdovirus (HnBSTRV)
- *Sripuvirus madureira* — Sena Madureira virus (SMV)
- *Sripuvirus niakha* — Niakha virus (NIAV)
- *Sripuvirus sripur* — Sripur virus (SRIV)

Sunrhavirus
- *Sunrhavirus alexandria* — Burg el Arab virus (BEAV)
- *Sunrhavirus bimbo* — Bimbo virus (BBOV)
- *Sunrhavirus boteke* — Boteke virus (BTKV)
- *Sunrhavirus dillard* — Dillard's Draw virus (DDRV)
- *Sunrhavirus garba* — Garba virus (GARV)
- *Sunrhavirus harrison* — Harrison Dam virus (HARDV)
- *Sunrhavirus kolongo* — Kolongo virus (KOLV)
- *Sunrhavirus kwatta* — Kwatta virus (KWAV)
- *Sunrhavirus matariya* — Matariya virus (MTYV)
- *Sunrhavirus nasoule* — Nasoule virus (NASV)
- *Sunrhavirus oakvale* — Oak Vale virus (OVV)
- *Sunrhavirus ouango* — Ouango virus (OUAV)
- *Sunrhavirus sandjimba* — Sandjimba virus (SJAV)
- *Sunrhavirus sunguru* — Sunguru virus (SUNV)
- *Sunrhavirus walkabout* — Walkabout Creek virus (WACV)

Thriphrhavirus
- *Thriphrhavirus intonsa* — Hangzhou Frankliniella intonsa rhabdovirus 1 (HFIRV1)
- *Thriphrhavirus tabaci* — Thrips tabaci associated dimarhabdovirus 1 (TtaDRV1)

Tibrovirus
- *Tibrovirus alphaekpoma* — Ekpoma virus 1 (EKV1)
- *Tibrovirus beatrice* — Beatrice Hill virus (BHV)
- *Tibrovirus betaekpoma* — Ekpoma virus 2 (EKV2)
- *Tibrovirus coastal* — Coastal Plains virus (CPV)
- *Tibrovirus congo* — Bas-Congo virus (BASV)
- *Tibrovirus mundri* — Mundri virus (MUNV)
- *Tibrovirus sweetwater* — Sweetwater Branch virus (SWBV)
- *Tibrovirus tibrogargan* — Bivens Arm virus (BAV), Tibrogargan virus (TIBV)

Tupavirus
- *Tupavirus durham* — Durham virus (DURV)
- *Tupavirus klamath* — Klamath virus (KLAV)
- *Tupavirus lingker* — Wenzhou Myotis laniger tupavirus 1 (WMlaTV1)
- *Tupavirus pearsonii* — Wufeng Rhinolophus pearsonii tupavirus 1 (WRpeTV1)
- *Tupavirus tupaia* — tupaia rhabdovirus (TUPV)

Vesiculovirus
- *Vesiculovirus alagoas* — vesicular stomatitis Alagoas virus (VSAV)
- *Vesiculovirus bogdanovac* — Yug Bogdanovac virus (YBV)
- *Vesiculovirus carajas* — Carajás virus (CJSV)
- *Vesiculovirus chandipura* — Chandipura virus (CHPV)
- *Vesiculovirus cocal* — Cocal virus (COCV)
- *Vesiculovirus eptesicus* — American bat vesiculovirus (ABVV)
- *Vesiculovirus indiana* — vesicular stomatitis Indiana virus (VSIV)
- *Vesiculovirus isfahan* — Isfahan virus (ISFV)
- *Vesiculovirus jurona* — Jurona virus (JURV)
- *Vesiculovirus malpais* — Malpais Spring virus (MSPV)
- *Vesiculovirus maraba* — Maraba virus (MARAV)
- *Vesiculovirus mediterranean* — Mediterranean bat virus (MBV)
- *Vesiculovirus mejal* — Mejal virus (MEJV)
- *Vesiculovirus morreton* — Morreton virus (MORV)
- *Vesiculovirus newjersey* — vesicular stomatitis New Jersey virus (VSNJV)
- *Vesiculovirus perinet* — Perinet virus (PERV)
- *Vesiculovirus piry* — Piry virus (PIRYV)
- *Vesiculovirus radi* — Radi virus (RADV)
- *Vesiculovirus rhinolophus* — Jinghong bat virus (JhBV), Qiongzhong bat virus (QZBV)
- *Vesiculovirus wufeng* — Wufeng Myotis altarium vesiculovirus 1 (WMAlVV1)
- *Vesiculovirus yinshui* — Yinshui bat virus (YSBV)

Zarhavirus
- *Zarhavirus zahedan* — Zahedan rhabdovirus (ZARV)

Betarhabdovirinae

Alphanucleorhabdovirus
- *Alphanucleorhabdovirus agavis* — Agave tequilana virus 1 (ATV1)
- *Alphanucleorhabdovirus artemisiae* — Artemisia capillaris nucleorhabdovirus 1 (ArtCaNV1)
- *Alphanucleorhabdovirus colocasiae* — taro vein chlorosis virus (TaVCV)
- *Alphanucleorhabdovirus constrictae* — constricta yellow dwarf virus (CYDV)
- *Alphanucleorhabdovirus joa* — joa yellow blotch associated virus (JYBaV)
- *Alphanucleorhabdovirus maydis* — maize mosaic virus (MMV)
- *Alphanucleorhabdovirus melongenae* — eggplant mottled dwarf virus (EMDV)
- *Alphanucleorhabdovirus morogoromaydis* — Morogoro maize-associated virus (MMaV)
- *Alphanucleorhabdovirus oryzae* — rice transitory yellowing virus (RTYV), rice yellow stunt virus (RYSV)
- *Alphanucleorhabdovirus physostegiae* — Physostegia chlorotic mottle virus (PhCMoV)
- *Alphanucleorhabdovirus pruni* — peach virus 1 (PeV1)
- *Alphanucleorhabdovirus tritici* — wheat yellow striate virus (WYSV)
- *Alphanucleorhabdovirus tuberosum* — potato yellow dwarf virus (PYDV)
- *Alphanucleorhabdovirus zeairanense* — maize Iranian mosaic virus (MIMV)

Betanucleorhabdovirus
- *Betanucleorhabdovirus alstroemeriae*
- *Betanucleorhabdovirus asclepiadis* — Asclepias syriaca virus 2 (AscSyV2)
- *Betanucleorhabdovirus bacopae* — Bacopa monnieri virus 2 (BmV2)
- *Betanucleorhabdovirus cardamomi* — cardamom vein clearing virus (CdVCV)
- *Betanucleorhabdovirus cnidii* — cnidium virus 1 (CnV1)
- *Betanucleorhabdovirus daturae* — datura yellow vein virus (DYVV)
- *Betanucleorhabdovirus loti* — birds-foot trefoil-associated virus (BFTV)
- *Betanucleorhabdovirus mali* — apple rootstock virus A (ApRVA)
- *Betanucleorhabdovirus medicagonis* — alfalfa-associated nucleorhabdovirus (AaNV)
- *Betanucleorhabdovirus plectranthi* — Plectranthus aromaticus virus 1 (PlAroV1)
- *Betanucleorhabdovirus retesonchi* — Sonchus yellow net virus (SYNV)
- *Betanucleorhabdovirus rhododendri* — Rhododendron delavayi virus 1 (RhoDeV1)
- *Betanucleorhabdovirus ribes* — blackcurrant-associated rhabdovirus (BCaRV)
- *Betanucleorhabdovirus venasonchi* — sowthistle yellow vein virus (SYVV)
- *Betanucleorhabdovirus zanthoxyli* — Zhuye pepper nucleorhabdovirus (ZPNRV)

Cytorhabdovirus
- *Cytorhabdovirus actinidiae* — Actinidia virus D (AcVD)
- *Cytorhabdovirus alphafrifolii* — Trifolium pratense virus A (TpVA)
- *Cytorhabdovirus alphawuhaninsectum* — Wuhan insect virus 4 (WuIV4)
- *Cytorhabdovirus anthurii* — Anthurium amnicola virus 1 (AntAmV1)
- *Cytorhabdovirus asclepiadis* — Asclepias syriaca virus 1 (AscSyV1)
- *Cytorhabdovirus bacopae* — Bacopa monnieri virus 1 (BmV1)
- *Cytorhabdovirus bemisiae* — Bemisia tabaci-associated virus 1 (BeTaV1)
- *Cytorhabdovirus betatrifolii* — Trifolium pratense virus B (TpVB)
- *Cytorhabdovirus betawuhaninsectum* — Wuhan insect virus 5 (WuIV5)
- *Cytorhabdovirus brassicae* — broccoli necrotic yellows virus (BNYV)
- *Cytorhabdovirus brassicolae* — cabbage cytorhabdovirus 1 (CCyV1)
- *Cytorhabdovirus brassicaeoleraceae* — paper mulberry mosaic associated virus (PMuMaV)
- *Cytorhabdovirus caricae* — papaya virus E (PpVE)
- *Cytorhabdovirus chrysanthemi* — chrysanthemum yellow dwarf associated virus (CYDaV)
- *Cytorhabdovirus colocasiae* — Colocasia bobone disease-associated virus (CBDaV)
- *Cytorhabdovirus cucurbitae* — cucurbit cytorhabdovirus 1 (CuCV1)
- *Cytorhabdovirus festucae* — Festuca leaf streak virus (FLSV)
- *Cytorhabdovirus flavibarbamate* — yerba mate chlorosis-associated virus (YmCaV)
- *Cytorhabdovirus fragariae* — strawberry-associated virus 1 (SaV1)
- strawberry virus 1 (StrV1)
- *Cytorhabdovirus fragariarugosus* — strawberry crinkle virus (SCV)
- *Cytorhabdovirus gammawuhaninsectum* — Wuhan insect virus 6 (WuIV6)
- *Cytorhabdovirus glehniae* — Glehnia littoralis virus 1 (GlLV1)
- *Cytorhabdovirus gramineae* — northern cereal mosaic virus (NCMV)
- *Cytorhabdovirus hordei* — barley yellow striate mosaic virus (BYSMV)
- *Cytorhabdovirus kenyatuberosum* — Kenyan potato cytorhabdovirus (KePCyV)
- *Cytorhabdovirus lactucamaculante* — lettuce yellow mottle virus (LYMoV)
- *Cytorhabdovirus lactucanecante* — lettuce necrotic yellows virus (LNYV)
- *Cytorhabdovirus lycopersici* — tomato yellow mottle-associated virus (TYMaV)
- *Cytorhabdovirus maydis* — maize-associated cytorhabdovirus (MaCV)
- *Cytorhabdovirus maysflavistriatis* — maize yellow striate virus (MYSV)
- *Cytorhabdovirus medicaginis* — alfalfa dwarf virus (ADV)
- *Cytorhabdovirus nymphaeae* — Nymphaea alba virus 1 (NymAV1)
- *Cytorhabdovirus orchidaceae* — Gymnadenia densiflora virus 1 (GymDenV1)
- *Cytorhabdovirus oryzae* — rice stripe mosaic virus (RSMV)
- *Cytorhabdovirus persimmon* — persimmon virus A (PeVA)
- *Cytorhabdovirus rosae* — rose virus R (RVR)
- *Cytorhabdovirus rubus* — raspberry vein chlorosis virus (RVCV)
- *Cytorhabdovirus sonchi* — Sonchus virus (SonV)
- *Cytorhabdovirus tagetis* — Tagetes erecta virus 1 (TaEV1)
- *Cytorhabdovirus trachyspermi* — Trachyspermum ammi virus 1 (TrAV1)
- *Cytorhabdovirus trichosanthei* — Trichosanthes associated rhabdovirus 1 (TrARV1)
- *Cytorhabdovirus tritici* — wheat American striate mosaic virus (WASMV)
- *Cytorhabdovirus yerbamate* — yerba mate virus A (YmVA)

Dichorhavirus
- *Dichorhavirus citri* — citrus chlorotic spot virus (CiCSV)
- *Dichorhavirus clerodendri* — clerodendrum chlorotic spot virus (ClCSV)
- *Dichorhavirus coffeae* — coffee ringspot virus (CoRSV)
- *Dichorhavirus leprosis* — citrus leprosis virus N (CiLVN)
- *Dichorhavirus orchidaceae* — orchid fleck virus (OFV)

Gammanucleorhabdovirus
- *Gammanucleorhabdovirus maydis* — maize fine streak virus (MFSV)

Varicosavirus
- *Varicosavirus alli* — Allium angulosum virus 1 (AAnV1)
- *Varicosavirus alopecuri* — Alopecurus myosuroides varicosavirus 1 (AMVV1)
- *Varicosavirus brassicae* — Brassica rapa virus 1 (BrRV1)
- *Varicosavirus ipomoeae* — morning glory varicosavirus (MGVV)
- *Varicosavirus lactucae* — lettuce big-vein associated virus (LBVaV)
- *Varicosavirus lolii* — Lolium perenne virus 1 (LoPV1)
- *Varicosavirus melampyri* — Melampyrum roseum virus 1 (MelRoV1)
- *Varicosavirus pini* — Pinus flexilis virus 1 (PiFleV1)
- *Varicosavirus trifolii* — red clover associated varicosavirus (RCaVV)
- *Varicosavirus vitis* — Vitis varicosavirus (VVV)
- *Varicosavirus xinjiangense* — Xinjiang varicosavirus (XVV)
- *Varicosavirus zosterae* — Zostera-associated varicosavirus 1 (ZaVV1)

Gammarhabdovirinae

Novirhabdovirus
- *Novirhabdovirus hirame* — hirame rhabdovirus (HIRRV = HIRV)
- *Novirhabdovirus piscine* — viral hemorrhagic septicemia virus (VHSV)
- *Novirhabdovirus salmonid* — infectious hematopoietic necrosis virus (IHNV)
- *Novirhabdovirus snakehead* — snakehead rhabdovirus (SHRV)

Unassigned

Alphacrustrhavirus
- *Alphacrustrhavirus wenling* — Wenling crustacean virus 10 (WlCV10)
- *Alphacrustrhavirus zhejiang* — Wenling crustacean virus 11 (WlCV11)

Alphadrosrhavirus
- *Alphadrosrhavirus hubei* — Wuhan house fly virus 2 (WhHFV2)
- *Alphadrosrhavirus shayang* — Shayang fly virus 3 (SyFV3)

Alphahymrhavirus
- *Alphahymrhavirus cinereus* — hymenopteran rhabdo-related virus 38 (HyRRV38)
- *Alphahymrhavirus hirtum* — hymenopteran rhabdo-related virus 109 (HyRRV109)
- *Alphahymrhavirus neglectus* — Lasius neglectus virus 2 (LnegV2)
- *Alphahymrhavirus radians* — hymenopteran rhabdo-related virus 46 (HyRRV46)

Betahymrhavirus
- *Betahymrhavirus austriaca* — hymenopteran rhabdo-related virus 23 (HyRRV23)
- *Betahymrhavirus heterodontonyx* — hymenopteran rhabdo-related virus 24 (HyRRV24)

Betanemrhavirus
- *Betanemrhavirus hubei* — Hubei rhabdo-like virus 9 (HbRLV9)
- *Betanemrhavirus shayang* — Shayang ascaridia galli virus 2 (SyAGV2)

Betapaprhavirus
- *Betapaprhavirus frugiperda* — Spodoptera frugiperda rhabdovirus (SfRV)
- *Betapaprhavirus sylvia* — lepidopteran rhabdo-related virus 34 (LeRRV34)

Betaricinrhavirus
- *Betaricinrhavirus chimay* — Chimay rhabdovirus (CRV)
- *Betaricinrhavirus scapularis* — blacklegged tick rhabdovirus 1 (BLTRV1)

Sunviridae

Sunshinevirus
- *Sunshinevirus reptilis* — Sunshine Coast virus (SunCV)

Xinmoviridae

Alasvirus
- *Alasvirus muscae* — Húběi diptera virus 11 (HbDV11)

Anphevirus
- *Anphevirus xinchengense* — Xīnchéng mosquito virus (XcMV)

Doupovirus
- *Doupovirus australiaense* — Culex mononega-like virus 2 (CMLV2)

Draselvirus
- *Draselvirus dentati* — Húběi rhabdo-like virus 7 (HbRLV7)

Drunivirus
- *Drunivirus chambonense* — Drosophila unispina virus 1 (DunIV1)

Gambievirus
- *Gambievirus bolahunense* — Bolahun virus (BLHV)
- *Gambievirus senegalense* — Gambie virus (GAMV)

Gylbovirus
- *Gylbovirus aegypti* — Aedes anphevirus (AeAV)

Hoptevirus
- *Hoptevirus orthopteris* — Húběi orthoptera virus 5 (HbOV5)

Madalivirus
- *Madalivirus amapaense* — Anopheles marajoara virus (AnMV)
- *Madalivirus amazonense* — Anopheles darlingi virus (AnDV)

Pelmivirus
- *Pelmivirus eymattense* — hymenopteran anphe-related virus OKIAV71 (HARV71)

Trinivirus
- *Trinivirus yonagoense* — Culex tritaeniorhynchus anphevirus (CTAV)

Ulegvirus
- *Ulegvirus freckenfeldense* — odonatan anphe-related virus OKIAV59 (OARV59)

Phylum
Subphylum
Class
Order
Family
Subfamily
Genus
Species
Virus (abbreviation)

[1]Includes: Newcastle disease virus (NDV)

2023 taxonomy of phylum *Negarnaviricota* (*Riboviria: Orthornavirae*): subphylum *Polyploviricotina* and unassigned

Kuhn JH *et al.* (2023) 2023 taxonomic update of phylum *Negarnaviricota (Riboviria: Orthornavirae)*, including the large orders *Bunyavirales* and *Mononegavirales*.

Negarnaviricota

Haploviricotina — *Polyploviricotina* — Unassigned

Ellioviricetes

Bunyavirales

Arenaviridae
- *Antennavirus*
  - *Antennavirus hirsutum* — Wēnlǐng frogfish arenavirus 2 (WlFAV2)
  - *Antennavirus salmonis* — salmon pescarenavirus 1 (SPAV1); salmon pescarenavirus 2 (SPAV2)
  - *Antennavirus striale* — Wēnlǐng frogfish arenavirus 1 (WlFAV1)
- *Hartmanivirus*
  - *Hartmanivirus brazilense* — SetPatVet virus 1 (SPVV1)
  - *Hartmanivirus haartmani* — Haartman Institute snake virus 1 (HISV1); Haartman Institute snake virus 2 (HISV2)
  - *Hartmanivirus helvetiae* — Dante Muikkunen virus 1 (DaMV1)
  - *Hartmanivirus patriae* — andere Heimat virus 1 (aHeV1)
  - *Hartmanivirus quadrati* — big electron-dense squares virus 1 (BESV1)
  - *Hartmanivirus scholae* — old schoolhouse virus 1 (OScV1); old schoolhouse virus 2 (OScV2)
  - *Hartmanivirus turici* — veterinary pathology Zurich virus 1 (VPZV1); veterinary pathology Zurich virus 2 (VPZV2)
  - *Hartmanivirus unni* — Universidad Nacional virus 1 (UnNV1)
- *Innmovirus*
  - *Innmovirus hailarense* — Hailar virus (HLRV)
- *Mammarenavirus*
  - *Mammarenavirus alashanense* — Alxa virus (ALXV)
  - *Mammarenavirus allpahuayoense* — Allpahuayo virus (ALLV)
  - *Mammarenavirus amaparienese* — Amapari virus (AMAV)
  - *Mammarenavirus aporeense* — Aporé virus (APOV)
  - *Mammarenavirus bearense* — Bear Canyon virus (BCNV)
  - *Mammarenavirus bituense* — Bitu virus (BITV)
  - *Mammarenavirus brazilense* — Sabiá virus (SBAV)
  - *Mammarenavirus caliense* — Pichindé virus (PICHV)
  - *Mammarenavirus caroneense* — souris virus (SOUV)
  - *Mammarenavirus chapareense* — Chapare virus (CHAPV)
  - *Mammarenavirus choriomeningitidis* — Dandenong virus (DANV); lymphocytic choriomeningitis virus (LCMV)
  - *Mammarenavirus cupixiense* — Cupixi virus (CUPXV)
  - *Mammarenavirus dhati-welelense* — Dhati Welel virus (DHWV)
  - *Mammarenavirus flexalense* — Flexal virus (FLEXV)
  - *Mammarenavirus gairoense* — Gairo virus (GAIV)
  - *Mammarenavirus guanaritoense* — Guanarito virus (GTOV)
  - *Mammarenavirus ippyense* — Ippy virus (IPPYV)
  - *Mammarenavirus juninense* — Junín virus (JUNV)
  - *Mammarenavirus kitaleense* — Kitale virus (KTLV)
  - *Mammarenavirus kwanzaense* — Kwanza virus (KWAV)
  - *Mammarenavirus lassaense* — Lassa virus (LASV)
  - *Mammarenavirus latinum* — Latino virus (LATV)
  - *Mammarenavirus lijiangense* — Lìjiāng virus (LIJV)
  - *Mammarenavirus loeiense* — Loei River virus (LORV)
  - *Mammarenavirus lujoense* — Lujo virus (LUJV)
  - *Mammarenavirus lunaense* — Lull virus (LLUV); Luna virus (LUAV)
  - *Mammarenavirus lunkense* — Lunk virus (LNKV)
  - *Mammarenavirus machupoense* — Machupo virus (MACV)
  - *Mammarenavirus marientalense* — Mariental virus (MRLV)
  - *Mammarenavirus merinoense* — Merino Walk virus (MRWV)
  - *Mammarenavirus mopeiaense* — Mopeia virus (MPOV); Morogoro virus (MORV)
  - *Mammarenavirus okahandjaense* — Okahandja virus (OKAV)
  - *Mammarenavirus oliverosense* — Oliveros virus (OLVV)
  - *Mammarenavirus paranaense* — Paraná virus (PRAV)
  - *Mammarenavirus piritalense* — Pirital virus (PIRV)
  - *Mammarenavirus praomyidis* — mobala virus (MOBV)
  - *Mammarenavirus ryukyuense* — Ryukyu virus (RYKV)
  - *Mammarenavirus solweziense* — Solwezi virus (SOLV)
  - *Mammarenavirus tacaribeense* — Tacaribe virus (TCRV)
  - *Mammarenavirus tamiamiense* — Tamiami virus (TMMV)
  - *Mammarenavirus wenzhouense* — Wēnzhōu virus (WENV)
  - *Mammarenavirus whitewaterense* — Big Brushy Tank virus (BBRTV); Catarina virus (CTNV); Skinner Tank virus (SKTV); Tonto Creek virus (TTCV); Whitewater Arroyo virus (WWAV)
  - *Mammarenavirus xapuriense* — Xapuri virus (XAPV)
- *Reptarenavirus*
  - *Reptarenavirus aurei* — Golden Gate virus (GOGV)
  - *Reptarenavirus californiae* — CAS virus (CASV)
  - *Reptarenavirus commune* — tavallinen suomalainen mies virus 2 (TSMV2)
  - *Reptarenavirus giessenae* — University of Giessen virus 1 (UGV1); University of Giessen virus 2 (UGV2); University of Giessen virus 3 (UGV3)
  - *Reptarenavirus rotterdamense* — ROUT virus (ROUTV); University of Helsinki virus 1 (UHV1)

Cruliviridae
- *Lincruvirus*
  - *Lincruvirus europense* — European shore crab virus 1 (EscV1)
  - *Lincruvirus sinense* — Chinese mitten crab virus 1 (CmcV1)
  - *Lincruvirus wenlingense* — Wēnlǐng crustacean virus 9 (WlCV9)

Discoviridae
- *Orthodiscovirus*
  - *Orthodiscovirus coniellae* — Coniothyrium diplodiella negative-stranded RNA virus 1 (CdNSRV1); Plasmopara viticola lesion associated mycobunyaviralés-like virus 8 (PvLAM-LV8)
  - *Orthodiscovirus hispaniae* — Plasmopara viticola lesion associated mycobunyaviralés-like virus 9 (PvLAM-LV9)
  - *Orthodiscovirus iberiae* — Plasmopara viticola lesion associated mycobunyaviralés-like virus 4 (PvLAM-LV4)
  - *Orthodiscovirus missouriense* — Penicillium discovirus (PDV)
  - *Orthodiscovirus penicillii* — Penicillium roseopurpureum negative stranded RNA virus 1 (PrNssV1)

Fimoviridae
- *Emaravirus*
  - *Emaravirus aceris* — maple mottle-associated virus (MaMaV)
  - *Emaravirus actinidiae* — Actinidia chlorotic ringspot-associated virus (AcCRaV)
  - *Emaravirus cajani* — pigeonpea sterility mosaic virus 1 (PPSMV1)
  - *Emaravirus camelliae* — Camellia japonica-associated virus 1 (CJaV1)
  - *Emaravirus cercidis* — redbud yellow ringspot-associated virus (RYRaV)
  - *Emaravirus chrysanthemi* — chrysanthemum mosaic-associated virus (ChMaV)
  - *Emaravirus cordylinae* — ti ringspot-associated virus (TiRSaV)
  - *Emaravirus corynocarpi* — karaka Ōkahu purepure emaravirus (KOPV)
  - *Emaravirus fici* — fig mosaic virus (FMV)
  - *Emaravirus fraxini* — ash shoestring-associated virus (ASaV)
  - *Emaravirus idaeobati* — raspberry leaf blotch virus (RLBV)
  - *Emaravirus illicii* — Japanese star anise ringspot-associated virus (JSARaV)
  - *Emaravirus kiwii* — Actinidia virus 2 (AcV2)
  - *Emaravirus parkinsoniae* — palo verde broom virus (PVBV)
  - *Emaravirus perillae* — perilla mosaic virus (PerMV)
  - *Emaravirus pistaciae* — Pistacia virus B (PiVB)
  - *Emaravirus populi* — aspen mosaic-associated virus (AsMaV)
  - *Emaravirus pyri* — pear chlorotic leaf spot-associated virus (PCLSaV)
  - *Emaravirus quercus* — common oak ringspot-associated virus (CORaV)
  - *Emaravirus rosae* — rose rosette virus (RRV)
  - *Emaravirus rubi* — blackberry leaf mottle-associated virus (BLMaV)
  - *Emaravirus sorbi* — European mountain ash ringspot-associated virus (EMARaV)
  - *Emaravirus syringae* — lilac chlorotic ringspot-associated virus (LiCRaV)
  - *Emaravirus toordali* — pigeonpea sterility mosaic virus 2 (PPSMV2)
  - *Emaravirus tritici* — High Plains wheat mosaic virus (HPWMoV)
  - *Emaravirus verbanni* — Camellia japonica-associated virus 2 (CjaV2)
  - *Emaravirus viscі* — Arceuthobium sichuanense-associated virus 1 (ArSaV1)
  - *Emaravirus vitis* — Vitis emaravirus (VEV)
  - *Emaravirus ziziphi* — jujube yellow mottle-associated virus (JYMaV)

Hantaviridae
- *Actantavirinae*
  - *Actinovirus*
    - *Actinovirus bernense* — Bern perch virus (BRPV)
    - *Actinovirus halieutaeae* — Wēnlǐng minipizza batfish virus (WEMBV)
    - *Actinovirus lophii* — Wēnlǐng yellow goosefish virus (WEYGV)
    - *Actinovirus triacanthodis* — Wēnlǐng red spikefish virus (WERSV)
- *Agantavirinae*
  - *Agnathovirus*
    - *Agnathovirus eptatreti* — Wēnlǐng hagfish virus (WEHV)
- *Mammantavirinae*
  - *Loanvirus*
    - *Loanvirus brunaense* — Brno virus (BRNV)
    - *Loanvirus longquanense* — Lóngquán virus (LQUV)
  - *Mobatvirus*
    - *Mobatvirus laibinense* — Láibīn virus (LAIV)
    - *Mobatvirus lenaense* — Lena virus (LENV)
    - *Mobatvirus novaense* — Nova virus (NVAV)
    - *Mobatvirus quezonense* — Quezon virus (QZNV)
    - *Mobatvirus xuansonense* — Xuân Sơn virus (XSV)
  - *Orthohantavirus*
    - *Orthohantavirus andesense* — Andes virus (ANDV); Castelo dos Sonhos virus (CASV); Lechiguanas virus (LECV = LECHV); Orán virus (ORNV)
    - *Orthohantavirus asamaense* — Asama virus (ASAV)
    - *Orthohantavirus asikkalaense* — Asikkala virus (ASIV)
    - *Orthohantavirus bayoui* — bayou virus (BAYV); Catacamas virus (CATV)
    - *Orthohantavirus boweense* — Bowé virus (BOWV)
    - *Orthohantavirus brugesense* — Bruges virus (BRGV)
    - *Orthohantavirus caobangense* — Cao Bằng virus (CBNV); Liánghé virus (LHEV)
    - *Orthohantavirus chocloense* — Choclo virus (CHOV)
    - *Orthohantavirus dabieshanense* — Dàbiéshān virus (DBSV)
    - *Orthohantavirus delgaditoense* — Caño Delgadito virus (CADV)
    - *Orthohantavirus dobravaense* — Dobrava virus (DOBV); Kurkino virus (KURV); Saaremaa virus (SAAV); Sochi virus (SOCV)
    - *Orthohantavirus fugongense* — Fúgòng virus (FUGV)
    - *Orthohantavirus fusongense* — Fǔsōng virus (FUSV)
    - *Orthohantavirus hantanense* — Amur virus (AMRV); Hantaan virus (HTNV); Soochong virus (SOOV)
    - *Orthohantavirus jejuense* — Jeju virus (JJUV)
    - *Orthohantavirus kenkemeense* — Kenkeme virus (KKMV)
    - *Orthohantavirus khabarovskense* — Khabarovsk virus (KHAV); Topografov virus (TOPV)
    - *Orthohantavirus luxiense* — Lúxī virus (LUXV)
    - *Orthohantavirus maporalense* — Maporal virus (MAPV)
    - *Orthohantavirus montanoense* — Montaño virus (MTNV)
    - *Orthohantavirus moroense* — Carrizal virus (CARV); El Moro Canyon virus (ELMCV); Huitzilac virus (HUIV)
    - *Orthohantavirus necoclíense* — Necoclí virus (NECV)
    - *Orthohantavirus negraense* — Laguna Negra virus (LANV); Maripa virus (MARV); Rio Mamoré virus (RIOMV)
    - *Orthohantavirus nigrorivense* — Black Creek Canal virus (BCCV)
    - *Orthohantavirus oxbowense* — Oxbow virus (OXBV)
    - *Orthohantavirus prospectense* — Prospect Hill virus (PHV)
    - *Orthohantavirus puumalaense* — Hokkaido virus (HOKV); Muju virus (MUJV); Puumala virus (PUUV)
    - *Orthohantavirus robinaense* — Robina virus (ROBV)
    - *Orthohantavirus rockportense* — Rockport virus (RKPV)
    - *Orthohantavirus sangassouense* — Sangassou virus (SANGV)
    - *Orthohantavirus seewisense* — Seewis virus (SWSV)
    - *Orthohantavirus seoulense* — gōu virus (GOUV); Seoul virus (SEOV)
    - *Orthohantavirus sinnombreense* — New York virus (NYV); Sin Nombre virus (SNV)
    - *Orthohantavirus tatenalense* — Tatenale virus (TATV)
    - *Orthohantavirus thailandense* — Anjozorobe virus (ANJZV); Serang virus (SERV); Thailand virus (THAIV)
    - *Orthohantavirus tigrayense* — Tigray virus (TIGV)
    - *Orthohantavirus tulaense* — Adler virus (ADLV); Tula virus (TULV)
    - *Orthohantavirus yakeshiense* — Yákèshí virus (YKSV)
- *Repantavirinae*
  - *Reptillovirus*
    - *Reptillovirus hemidactyli* — Hǎinán oriental leaf-toed gecko virus (HOLGV)
- *Thottimvirus*
  - *Thottimvirus imjinense* — Imjin virus (MJNV)
  - *Thottimvirus thottapalayamense* — Thottapalayam virus (TPMV)

Leishbuviridae
- *Shilevirus*
  - *Shilevirus leptomonadis* — Leptomonas moramango virus (LEPMV)

Mypoviridae
- *Hubavirus*
  - *Hubavirus myriapedis* — Húběi myriapoda virus 5 (HbMV5)

Nairoviridae
- *Norwavirus*
  - *Norwavirus beijiense* — Běijī nairovirus (BJNV)
  - *Norwavirus grotenhoutense* — Grotenhout virus (GRHV)
- *Ocetevirus*
  - *Ocetevirus paratemnopterygis* — red goblin roach virus 1 (RGRV1)
- *Orthonairovirus*
  - *Orthonairovirus abuhammadense* — Abū Hammād virus (AHV)
  - *Orthonairovirus abuminaense* — Abū Mīnā virus (AMV)
  - *Orthonairovirus amblyommae* — lupe virus (KUPEV)
  - *Orthonairovirus artashatense* — Artashat virus (ARTSV)
  - *Orthonairovirus australiaense* — Vinegar Hill virus (VINHV)
  - *Orthonairovirus avalonense* — Avalon virus (AVAV)
  - *Orthonairovirus bandiaense* — Bandia virus (BDAV)
  - *Orthonairovirus buranaense* — Burana virus (BURV)
  - *Orthonairovirus bushkeyense* — Caspiy virus (CASV); Farallon virus (FARV); Great Saltee virus (GRSV); Hughes virus (HUGV); Raza virus (RAZAV)
  - *Orthonairovirus chimense* — Chim virus (CHIMV)
  - *Orthonairovirus clomorense* — Clo Mor virus (CMV = CLMV)
  - *Orthonairovirus dermacentoris* — Pacific Coast tick nairovirus (PCTNV)
  - *Orthonairovirus dugbeense* — Dugbe virus (DUGV)
  - *Orthonairovirus erveense* — Erve virus (ERVEV)
  - *Orthonairovirus esteroense* — Estero Real virus (ERV)
  - *Orthonairovirus gossasense* — Gossas virus (GOSV)
  - *Orthonairovirus haemorrhagiae* — Crimean-Congo hemorrhagic fever virus (CCHFV)
  - *Orthonairovirus hazaraense* — Hazara virus (HAZV)
  - *Orthonairovirus huangpiense* — Huángpí tick virus 1 (HpTV1)
  - *Orthonairovirus issykkulense* — Issyk-kul virus (ISKV)
  - *Orthonairovirus japonicum* — tofla virus (TFLV)
  - *Orthonairovirus kasokeroense* — Kasokero virus (KASV = KASOV)
  - *Orthonairovirus keterahense* — Keterah virus (KTRV); Uzun-Agach virus (UZAV)
  - *Orthonairovirus khani* — Dera Ghazi Khan virus (DGKV)
  - *Orthonairovirus lusakaense* — Leopards Hill virus (LPHV)
  - *Orthonairovirus macquariense* — Taggert virus (TAGV)
  - *Orthonairovirus meramense* — Meram virus (MEMV)
  - *Orthonairovirus nairobiense* — Nairobi sheep disease virus (NSDV)
  - *Orthonairovirus parahaemorrhagiae* — Aigai virus (AIGV)
  - *Orthonairovirus peruense* — Punta Salinas virus (PSV)
  - *Orthonairovirus qalyubense* — Geran virus (GERV); Qalyub virus (QYBV)
  - *Orthonairovirus randolense* — Sapphire II virus (SAPV)
  - *Orthonairovirus sakhalinense* — Sakhalin virus (SAKV); Tillamook virus (TILLV)
  - *Orthonairovirus soldadoense* — Soldado virus (SOLV)
  - *Orthonairovirus songlingense* — Sōnglǐng virus (SGLV)
  - *Orthonairovirus sulinaense* — Sulina virus (SULV)
  - *Orthonairovirus tachengense* — Tǎchéng tick virus 1 (TcTV1)
  - *Orthonairovirus thiaforaense* — Thiafora virus (TFAV)
  - *Orthonairovirus tamdiense* — Tamdy virus (TAMV)
  - *Orthonairovirus tunisense* — Tunis virus (TUNV)
  - *Orthonairovirus wenzhouense* — Wēnzhōu tick virus (WzTV)
  - *Orthonairovirus yezoense* — Yezo virus (YEZV)
  - *Orthonairovirus yogueense* — Yogue virus (YOGV)
  - *Orthonairovirus zirkuense* — Zirqa virus (ZIRV)
- *Sabavirus*
  - *Sabavirus americanum* — South Bay virus (SBV)
- *Shaspivirus*
  - *Shaspivirus aranei* — Shāyáng spider virus 1 (SySV1)
- *Striwavirus*
  - *Striwavirus saxnsiense* — Sānxiá water strider virus 1 (SxWSV1)
- *Xinspivirus*
  - *Xinspivirus xinzhouense* — Xīnzhōu spider virus (XSV)

Peribunyaviridae
- *Herbevirus*
  - *Herbevirus herberti* — Herbert virus (HEBV)
  - *Herbevirus kibaleense* — Kibale virus (KIBV)
  - *Herbevirus taiense* — Tai virus (TAIV)
- *Khurdivirus*
  - *Khurdivirus volgaense* — Khurdun virus (KURV)
- *Lakivirus*
  - *Lakivirus lakamhaense* — Lakamha virus (LAKV)
- *Lambavirus*
  - *Lambavirus wisconsinense* — largemouth bass bunyavirus (LBBV)
- *Orthobunyavirus*
  - *Orthobunyavirus abrasense* — Abras virus (ABRV)
  - *Orthobunyavirus acaraense* — Acará virus (ACAV); Benevides virus (BVS = BNV)
  - *Orthobunyavirus achiotei* — Achiote virus (ACHOV); Trivittatus virus (TVTV)
  - *Orthobunyavirus ainoense* — Aino virus (AINOV); Kaikalur virus (KAIV)
  - *Orthobunyavirus akabaneense* — Akabane virus (AKAV); Tinaroo virus (TINV)
  - *Orthobunyavirus anadyrense* — Anadyr virus (ANADV)
  - *Orthobunyavirus ananindeuaense* — Ananindeua virus (ANUV)
  - *Orthobunyavirus angeloense* — San Angelo virus (SAV)
  - *Orthobunyavirus anhembiense* — Anhembi virus (AMBV)
  - *Orthobunyavirus apeuense* — Apeú virus (APEUV)
  - *Orthobunyavirus baakalense* — Baakal virus (BKAV)
  - *Orthobunyavirus bakaueense* — Bakau virus (BAKV); Tanjong Rabok virus (TRV)
  - *Orthobunyavirus balagodense* — Balagodu virus (BLGV)
  - *Orthobunyavirus bataiense* — Batai virus (BATV)
  - *Orthobunyavirus batamaense* — Batama virus (BMAV)
  - *Orthobunyavirus belemense* — Belém virus (BLMV)
  - *Orthobunyavirus bellavistaense* — Antequera virus (ANTV); Barranqueras virus (BQSV); Bellavista virus (BELLV); Resistencia virus (RTAV)
  - *Orthobunyavirus benficaense* — Benfica virus (BENV = BNFV)
  - *Orthobunyavirus bertiogaense* — Bertioga virus (BERV); Cananéia virus (CNAV)
  - *Orthobunyavirus biraoense* — Birao virus (BIRV)
  - *Orthobunyavirus bobayaense* — Bobaya virus (BOBV)
  - *Orthobunyavirus boraceiaense* — Boracéia virus (BORV)
  - *Orthobunyavirus botambiense* — Botambi virus (BOTV)
  - *Orthobunyavirus bozoense* — Bozo virus (BOZOV)
  - *Orthobunyavirus brazoranense* — brazoran virus (BRAZV)
  - *Orthobunyavirus bruconhaense* — Bruconha virus (BRUV)
  - *Orthobunyavirus buffaloense* — Buffalo Creek virus (BUCV); Murrumbidgee virus (MURBV); Mitchell River virus (MRV) [illegible]
  - *Orthobunyavirus bunyamweraense* — Bunyamwera virus (BUNV); Germiston virus (GERV); Mboké virus (MBOV); Ngari virus (NRIV); Stanfield virus (STAV); Xingu virus (XINV)
  - *Orthobunyavirus bushbushense* — Bushbush virus (BSBV); Juan Díaz virus (JDV) [illegible]
  - *Orthobunyavirus buttonwillowense* — Buttonwillow virus (BUTV)
  - *Orthobunyavirus bwambaense* — Bwamba virus (BWAV); Pongola virus (PGAV)
  - *Orthobunyavirus cacheense* — Cache Valley virus (CVV); Cholul virus (CHLV); Playas virus (PLAV); Tlacotalpan virus (TLAV)
  - *Orthobunyavirus capimense* — Capim virus (CAPV)
  - *Orthobunyavirus caraparuense* — Caraparú virus (CARV); El Huayo virus (EHUV); Itaquí virus (ITQV); Itaya virus (ITYV); Ossa virus (OSSAV)
  - *Orthobunyavirus catqueense* — Cat Qué virus (CQV); Oya virus (OYAV)
  - *Orthobunyavirus catuense* — Catú virus (CATUV); Moju virus (MOJUV)
  - *Orthobunyavirus cuchilloense* — Anopheles B virus (ANBV)
  - *Orthobunyavirus ebiense* — Ebinur Lake virus (EBIV)
  - *Orthobunyavirus encephalitidis* — California encephalitis virus (CEV); Morro Bay virus (MBV)
  - *Orthobunyavirus enseadaense* — Enseada virus (ENSV)
  - *Orthobunyavirus faceyense* — Facey's paddock virus (FPV)
  - *Orthobunyavirus gamboaense* — Alajuela virus (ALJV); Brus Laguna virus (BLAV); Calchaqui virus (CQIV); Gamboa virus (GAMV); Soberanía virus (SOBV)
  - *Orthobunyavirus ganganense* — Gan Gan virus (GGV); Salt Ash virus (SASHV)
  - *Orthobunyavirus guajaraense* — Guajará virus (GJAV)
  - *Orthobunyavirus guamaense* — Bimiti virus (BIMV); Guamá virus (GMAV)
  - *Orthobunyavirus guaratubaense* — Guaratuba virus (GTBV); Itimirim virus (ITIV)
  - *Orthobunyavirus guaroaense* — Guaroa virus (GROV)
  - *Orthobunyavirus gumbolimboense* — Gumbo Limbo virus (GLV)
  - *Orthobunyavirus heptaybaense* — Yaba-7 virus (Y7V)
  - *Orthobunyavirus horizonteense* — Anopheles A virus (ANAV); Arumateua virus (ARTV = ARMTV); Caraipé virus (CPEV = CRPV); Trombetas virus (TRMV); Tucuruí virus (TUCV = TUCRV)
  - *Orthobunyavirus iacoense* — Iaco virus (IACOV)
  - *Orthobunyavirus ileshaense* — Ilesha virus (ILEV)
  - *Orthobunyavirus infirmati* — Infirmatus virus (INFV)
  - *Orthobunyavirus ingwavumaense* — Ingwavuma virus (INGV)
  - *Orthobunyavirus insulae* — kairi virus (KRIV)
  - *Orthobunyavirus jamestownense* — Inkoo virus (INKV); Jamestown Canyon virus (JCV); Jerry Slough virus (JSV); South River virus (SORV)
  - *Orthobunyavirus jatobalense* — Jatobal virus (JATV)
  - *Orthobunyavirus juandiazense* — Juan Díaz virus (JDV)
  - *Orthobunyavirus kaengkhoiense* — Kaeng Khoi virus (KKV)
  - *Orthobunyavirus keystoneense* — Lokern virus (LOKV); Main Drain virus (MDV); Santa Rosa virus (SARV)
  - *Orthobunyavirus ketapangense* — Ketapang virus (KETV)
  - *Orthobunyavirus keystoneense* — Keystone virus (KEYV)
  - *Orthobunyavirus khatangaense* — Khatanga virus (KHATV); snowshoe hare virus (SSHV)
  - *Orthobunyavirus koongolense* — koongol virus (KOOV); wongal virus (WONV)
  - *Orthobunyavirus kowanyamaense* — Kowanyama virus (KOWV)
  - *Orthobunyavirus lacrosseense* — La Crosse virus (LACV)
  - *Orthobunyavirus lasmaloyasense* — Las Maloyas virus (LMV)
  - *Orthobunyavirus leanyerense* — Leanyer virus (LEAV)
  - *Orthobunyavirus lednicense* — Lednice virus (LEDV)
  - *Orthobunyavirus lukuniense* — Lukuni virus (LUKV)
  - *Orthobunyavirus lumboense* — Lumbo virus (LUMV)
  - *Orthobunyavirus macauaense* — Macauã virus (MCAV)
  - *Orthobunyavirus madridense* — Madrid virus (MADV); Vinces virus (VINV)
  - *Orthobunyavirus maguariense* — Maguari virus (MAGV)
  - *Orthobunyavirus mahoganyense* — Mahogany Hammock virus (MHV)
  - *Orthobunyavirus manzanillaense* — Manzanilla virus (MANV); Inini virus (INIV)
  - *Orthobunyavirus mapriкense* — Maprik virus (MPKV)
  - *Orthobunyavirus marituбaense* — Marituba virus (MTBV); Zungarococha virus (ZUNV); Murutucu virus (MURV) [illegible]
  - *Orthobunyavirus matruhense* — Matruh virus (MTRV)
  - *Orthobunyavirus melaojoense* — Melao virus (MELV)
  - *Orthobunyavirus mermetense* — Mermet virus (MERV)
  - *Orthobunyavirus minatitlanense* — Minatitlán virus (MNTV); Palestina virus (PLSV)
  - *Orthobunyavirus mirimense* — Mirim virus (MIRV)
  - *Orthobunyavirus mitchellense* — Mapputta virus (MAPV)
  - *Orthobunyavirus mpokoense* — M'Poko virus (MPOV); Yaba-1 virus (Y1V)
  - *Orthobunyavirus navioense* — Serra do Navio virus (SDNV)
  - *Orthobunyavirus nepuyoense* — Nepuyo virus (NEPV)
  - *Orthobunyavirus nessozionaense* — Ness Ziona virus (NZV)
  - *Orthobunyavirus nolaense* — Nola virus (NOLAV)
  - *Orthobunyavirus northwayense* — Northway virus (NORV)
  - *Orthobunyavirus nyandoense* — Eretmapodites virus (ERETV); Mojuí dos Campos virus (MDCV); Nyando virus (NDV)
  - *Orthobunyavirus okolaense* — Okola virus (OKOV)
  - *Orthobunyavirus olifantsvleiense* — Bobia virus (BIAV); Dabakala virus (DABV); Olifantsvlei virus (OLIV); Oubi virus (OUBIV)
  - *Orthobunyavirus oribocaense* — Murutucú virus (MURV); Oriboca virus (ORIV); Restan virus (RESV)
  - *Orthobunyavirus oropoucheense* — Iquitos virus (IQTV); Madre de Dios virus (MDDV); Oropouche virus (OROV); Perdões virus (PDEV); Pintupo virus (PINTV)
  - *Orthobunyavirus oyoense* — Oyo virus (OYOV)
  - *Orthobunyavirus pacoraense* — Pacora virus (PCAV)
  - *Orthobunyavirus patoisense* — Babahoya virus (BABV); Pahayokee virus (PAHV); Patois virus (PATV)
  - *Orthobunyavirus peachesterense* — peaton virus (PEAV)
  - *Orthobunyavirus porteiraense* — Cachoeira Porteira virus (CPOV)
  - *Orthobunyavirus potosiense* — Potosi virus (POTV)
  - *Orthobunyavirus saboense* — Sabo virus (SABOV)
  - *Orthobunyavirus sangoense* — Sango virus (SANV)
  - *Orthobunyavirus sanjuanense* — Pueblo Viejo virus (PVV); San Juan virus (SJV)
  - *Orthobunyavirus schmallenbergense* — Douglas virus (DOUV); Sathuperi virus (SATV); Schmallenberg virus (SBV); Shamonda virus (SHAV)
  - *Orthobunyavirus sedlecense* — Sedlec virus (SEDV)
  - *Orthobunyavirus shermanense* — Fort Sherman virus (FSV); Laguna Larga virus (LLV)
  - *Orthobunyavirus shokweense* — Shokwe virus (SHOV)
  - *Orthobunyavirus shuniense* — Shuni virus (SHUV)
  - *Orthobunyavirus simbuense* — Para virus (PARAV); Simbu virus (SIMV)
  - *Orthobunyavirus sororocaense* — Sororoca virus (SORV)
  - *Orthobunyavirus squalofluvii* — Shark River virus (SRV)
  - *Orthobunyavirus tacaiumaense* — CoAr 1071 virus (CA1071V); CoAr 3627 virus (CA3627V); Tacaiuma virus (TCMV); Virgin River virus (VRV)
  - *Orthobunyavirus tahynaense* — Ťahyňa virus (TAHV)
  - *Orthobunyavirus tangaense* — Tanga virus (TANV)
  - *Orthobunyavirus tataguineense* — Tataguine virus (TATV)
  - *Orthobunyavirus teloкense* — Telok Forest virus (TFV)
  - *Orthobunyavirus tensawense* — Tensaw virus (TENV)
  - *Orthobunyavirus termeilense* — Termeil virus (TERV)
  - *Orthobunyavirus teteense* — Bahig virus (BAHV); Tete virus (TETEV); Tsuruse virus (TSUV); Weldona virus (WELV)
  - *Orthobunyavirus thimiriense* — Thimiri virus (THIV)
  - *Orthobunyavirus timboteuaense* — Timboteua virus (TBTV)
  - *Orthobunyavirus triniti*ense — Triniti virus (TNTV)
  - *Orthobunyavirus turlockense* — Turlock virus (TURV)
  - *Orthobunyavirus umbreense* — Umbre virus (UMBV)
  - *Orthobunyavirus utingaense* — Utinga virus (UTIV)
  - *Orthobunyavirus witwatersrandense* — Witwatersrand virus (WITV)
  - *Orthobunyavirus wolkbergense* — Wolkberg virus (WBV)
  - *Orthobunyavirus wyeomyiae* — Rio Pracupí virus; Taiassuí virus (TAIAV); Tucunduba virus (TUCV); Wyeomyia virus (WYOV)
- *Pacuvirus*
  - *Pacuvirus caimitoense* — Caimito virus (CAIV)
  - *Pacuvirus chilibreense* — Chilibre virus (CHIV)
  - *Pacuvirus evaenseе* — Rio Preto da Eva virus (RPEV)
  - *Pacuvirus pacuiense* — Pacuí virus (PACV)
  - *Pacuvirus tapirapeense* — Tapirapé virus (TAPV)
- *Shangavirus*
  - *Shangavirus shuangaoense* — Shuāngáo insect virus 1 (SgIV1)

Phasmaviridae
- *Cicadellivirus*
  - *Cicadellivirus scaphoideі* — Scaphoideus titanus bunya-like virus 1 (StBV)
- *Feravirus*
  - *Feravirus ferakinum* — Ferak virus (FRKV)
  - *Feravirus guaguaense* — Guaguá virus (GUAV)
  - *Feravirus hemipterus* — hemipteran phasma-related virus OKIAV247 (HePV)
  - *Feravirus neuropterus* — neuropteran phasma-related virus OKIAV248 (NePV)
- *Hymovirus*
  - *Hymovirus chrysis* — hymenopteran phasma-related virus OKIAV250 (HyHV2)
  - *Hymovirus chrysurae* — hymenopteran phasma-related virus OKIAV252 (HyHV1)
- *Jonvirus*
  - *Jonvirus eboris* — Jonchet virus (JONV)
- *Orthophasmavirus*
  - *Orthophasmavirus aedis* — Wǔhàn mosquito virus 2 (WhMV2)
  - *Orthophasmavirus anophelae* — Anopheles triannulatus orthophasmavirus (ATOPV)
  - *Orthophasmavirus barstukasense* — Barstukas virus (BARV)
  - *Orthophasmavirus chrysis* — hymenopteran phasma-related virus OKIAV227 (HyOV2)
  - *Orthophasmavirus coleopteri* — coleopteran phasma-related virus OKIAV235 (CPRV)
  - *Orthophasmavirus coredoense* — Coredo virus (CORV)
  - *Orthophasmavirus culicis* — Culex phasma-like virus (CPLV)
  - *Orthophasmavirus flenense* — Flen virus (FLNV)
  - *Orthophasmavirus fushuense* — Fúshǔn phasmavirus 1 (FsPV1)
  - *Orthophasmavirus gandeense* — Ganda bee virus (GBEEV)
  - *Orthophasmavirus hubeiense* — Húběi odonate virus 9 (HbOV9)
  - *Orthophasmavirus kiguiaense* — Kigluaik phantom virus (KIGV)
  - *Orthophasmavirus miglotaense* — Miglotas virus (MIGV)
  - *Orthophasmavirus niuklukense* — Niukluk phantom virus (NUKV)
  - *Orthophasmavirus odonatus* — Húběi odonate virus 8 (HbOV8)
  - *Orthophasmavirus philoctetis* — hymenopteran phasma-related virus OKIAV228 (HyOV1)
  - *Orthophasmavirus sogatellae* — Fúshǔn phasmavirus 2 (FsnPV2)
  - *Orthophasmavirus wuchangense* — Wǔchāng cockroach virus 1 (WcCV1)
  - *Orthophasmavirus wuhanense* — Wǔhàn mosquito virus 1 (WhMV1)
- *Sawastrivirus*
  - *Sawastrivirus sanxiaense* — Sānxiá water strider virus 2 (SxWSV2)
- *Wuhivirus*
  - *Wuhivirus insecti* — Wǔhàn insect virus 2 (WhIV2)

Phenuiviridae
- *Bandavirus*
  - *Bandavirus albatrossense* — Albatross Island virus (ABIV)
  - *Bandavirus amblyommae* — lone star virus (LSV)
  - *Bandavirus bhanjanagarense* — Bhanja virus (BHAV)
  - *Bandavirus dabieense* — severe fever with thrombocytopenia syndrome virus (SFTSV)
  - *Bandavirus guertuense* — Guertu virus (GTV)
  - *Bandavirus heartlandense* — Heartland virus (HRTV)
  - *Bandavirus kismayoense* — Kismayo virus (KISV)
  - *Bandavirus razdanense* — Razdan virus (RAZV)
  - *Bandavirus zwieselense* — Zwiesel bat bandavirus (ZbbV)
- *Beidivirus*
  - *Beidivirus muscae* — Húběi diptera virus 3 (HbDV3)
- *Citricivirus*
  - *Citricivirus chongqingense* — Aphis citricidus bunyavirus (ACBV)
- *Coguvirus*
  - *Coguvirus chinense* — Brassica campestris chinensis coguvirus 1 (BCCoV1)
  - *Coguvirus citri* — citrus concave gum-associated virus (CCGaV)
  - *Coguvirus citrulli* — watermelon crinkle leaf-associated virus 1 (WCLaV1)
  - *Coguvirus eburi* — citrus virus A (CiVA)
  - *Coguvirus henanense* — watermelon crinkle leaf-associated virus 2 (WCLaV2)
  - *Coguvirus viticulum* — grapevine associated cogu-like virus 1 (GaCLV1)
  - *Coguvirus yunnanense* — Yúnnán Paris negative-stranded virus (YPNSV)
- *Entovirus*
  - *Entovirus entoleucae* — Entoleuca phenui-like virus 1 (EnPLV1)
- *Goukovirus*
  - *Goukovirus aphalarae* — Aphalara polygoni bunya-like virus (ApBLV)
  - *Goukovirus cerapheri* — Cerapheron bunya-like virus (CerBLV)
  - *Goukovirus cumutoense* — Cumuto virus (CUMV)
  - *Goukovirus gouleakoense* — Gouléako virus (GOLV)
  - *Goukovirus yichangense* — Yíchāng insect virus (YcIV)
- *Horwuvirus*
  - *Horwuvirus fitzroyense* — Fitzroy Crossing tenui-like virus 1 (FCTenV1)
  - *Horwuvirus solenopsidis* — Solenopsis invicta virus 14 (SINV14)
  - *Horwuvirus wuhanense* — Wǔhàn horsefly virus (WhHV)
- *Hudivirus*
  - *Hudivirus muscae* — Húběi diptera virus 4 (HbDV4)
- *Hudovirus*
  - *Hudovirus lepidopteris* — Húběi lepidoptera virus 1 (HbLV1)
- *Ixovirus*
  - *Ixovirus heckscherense* — blacklegged tick virus 1 (BLTV1)
  - *Ixovirus ixodis* — blacklegged tick virus 3 (BLTV3)
  - *Ixovirus norvegiae* — Fairhair virus (FHAV)
- *Laulavirus*
  - *Laulavirus alphaviticulum* — grapevine associated cogu-like virus 2 (GaCLV2)
  - *Laulavirus betaviticulum* — grapevine associated cogu-like virus 3 (GaCLV3)
  - *Laulavirus gammaviticulum* — grapevine associated cogu-like virus 4 (GaCLV4)
  - *Laulavirus laurelense* — Laurel Lake virus (LLV)
  - *Laulavirus wardellense* — Wardell virus (WRDV)
- *Lentinuvirus*
  - *Lentinuvirus lentinulae* — Lentinula edodes negative-strand RNA virus 2 (LeNSRV2)
- *Mechlorovirus*
  - *Mechlorovirus cucumeris* — melon chlorotic spot virus (MeCSV)
- *Mobuvirus*
  - *Mobuvirus arnae* — Euproctis pseudoconspersa bunyavirus (EPBV)
  - *Mobuvirus mothrae* — Mothra virus (MTHV)
  - *Mobuvirus narangueense* — Narangue virus (NRGV)
  - *Mobuvirus stephanocirci* — Browner virus (BRWV)
- *Phasivirus*
  - *Phasivirus baduense* — Badu virus (BADUV)
  - *Phasivirus guadeloupeense* — Guadeloupe mosquito phasivirus (GMPV)
  - *Phasivirus hubeiense* — Húběi diptera virus 5 (HbDV5)
  - *Phasivirus parryense* — Parry's Creek phasivirus 1 (PCPhasV1)
  - *Phasivirus phasiense* — Phasi Charoen-like virus (PCLV)
  - *Phasivirus wuhanense* — Wǔhàn fly virus 1 (WhFV1)
  - *Phasivirus wutaiense* — Wūtái mosquito virus (WtMV)
- *Phlebovirus*
  - *Frijoles phlebovirus* — frijoles virus (FRIV); Joá virus (JOAV)
  - *Phlebovirus adanaense* — Adana virus (ADAV)
  - *Phlebovirus aguacateense* — Aguacate virus (AGUV)
  - *Phlebovirus alcubeense* — Alcube virus (ACBV)
  - *Phlebovirus alenquerense* — Alenquer virus (ALEV)
  - *Phlebovirus almendrasense* — Tres Almendras virus (TRAV)
  - *Phlebovirus ambeense* — Ambé virus (ABEV)
  - *Phlebovirus anhangaense* — Anhangá virus (ANHV)
  - *Phlebovirus arumowotense* — Arumowot virus (AMTV)
  - *Phlebovirus bogoriaense* — Bogoria virus (BGRV)
  - *Phlebovirus buenaventuraense* — Buenaventura virus (BUEV)
  - *Phlebovirus bujaruense* — Bujaru virus (BUJV)
  - *Phlebovirus cacaoense* — Cacao virus (CACV)
  - *Phlebovirus campanaense* — Campana virus (CMAV)
  - *Phlebovirus candiruense* — Ariquemes virus (ARQV); Candiru virus (CDUV); Jacundá virus (JCNV); Morumbi virus (MRMBV); Mucura virus (MCRV/MRAV); Serra Norte virus (SRNV)
  - *Phlebovirus chagresense* — Chagres virus (CHGV)
  - *Phlebovirus clarorense* — Rio Claro virus (RICV)
  - *Phlebovirus cocleense* — Coclé virus (CCLV)
  - *Phlebovirus corfouense* — Corfou virus (CFUV)
  - *Phlebovirus dashliense* — Dāshlī virus (DASV)
  - *Phlebovirus duraniaense* — Durania virus (DRNV)
  - *Phlebovirus echarateense* — Echarate virus (ECHV)
  - *Phlebovirus embossosense* — Embossos virus (EMBV)
  - *Phlebovirus florisense* — Saint-Floris virus (SAFV)
  - *Phlebovirus gabekense* — Gabek forest virus (GFV)
  - *Phlebovirus gloriaense* — La Gloria virus (LAGV)
  - *Phlebovirus gordilense* — Gordil virus (GORV)
  - *Phlebovirus hedienese* — Hedi virus (HEDV)
  - *Phlebovirus icoaraciense* — Icoaraci virus (ICOV)
  - *Phlebovirus itaitubaense* — Itaituba virus (ITAV)
  - *Phlebovirus itaporangaense* — Itaporanga virus (ITPV)
  - *Phlebovirus ixcanalense* — Ixcanal virus (IXCV)
  - *Phlebovirus karimabadense* — Karimabad virus (KARV)
  - *Phlebovirus kiborgochense* — Kiborgoch virus (KBGV)
  - *Phlebovirus leticiaense* — Leticia virus (LTCV)
  - *Phlebovirus maldonadoense* — Maldonado virus (MLOV)
  - *Phlebovirus massiliaense* — Massilia virus (MASV)
  - *Phlebovirus medjerdaense* — Medjerda Valley virus (MVV)
  - *Phlebovirus monagritaense* — Mona Grita virus (MOGV)
  - *Phlebovirus mukawaense* — Mukawa virus (MKWV)
  - *Phlebovirus mungubaense* — Munguba virus (MUNV)
  - *Phlebovirus napoliense* — Arrábida virus (ARRV); Balkan virus (BALKV); Fermo virus (FERV); Granada virus (GRV = GRAV); Saddaguia virus (SADV); sandfly fever Naples virus (SFNV)
  - *Phlebovirus niqueense* — Nique virus (NIQV)
  - *Phlebovirus ntepesense* — Ntepes virus (NTPV)
  - *Phlebovirus odrenisrouense* — Odrénisrou virus (ODRV)
  - *Phlebovirus oriximinaense* — Oriximiná virus (ORXV)
  - *Phlebovirus pantanalense* — viola virus (VIOV)
  - *Phlebovirus penablancaense* — Peña Blanca virus (PEBV)
  - *Phlebovirus penshurtense* — Penshurt virus (PEHV)
  - *Phlebovirus perkeraense* — Perkerra virus (PKEV)
  - *Phlebovirus punique*ense — Punique virus (PUNV)
  - *Phlebovirus riftense* — Rift Valley fever virus (RVFV)
  - *Phlebovirus riograndeense* — Rio Grande virus (RGV)
  - *Phlebovirus salangaense* — Salanga virus (SLGV)
  - *Phlebovirus salehabadense* — Adria virus (ADRV); Arbia virus (ARBV); Bregalaka virus (BREV); Olbia virus (OLBV); Salehabad virus (SALV); Zaba virus (ZABAV)
  - *Phlebovirus saloboense* — Salobo virus (SLBOV)
  - *Phlebovirus siciliaense* — sandfly fever Sicilian virus (SFSV)
  - *Phlebovirus tapuraense* — Tapará virus (TPRV)
  - *Phlebovirus tehranense* — Tehran virus (THEV)
  - *Phlebovirus ticoense* — Tico virus (TICV)
  - *Phlebovirus toroense* — Capira virus (CAPIV); Punta Toro virus (PTV)
  - *Phlebovirus torosense* — Toros virus (TORV)
  - *Phlebovirus toscanaense* — Toscana virus (TOSV)
  - *Phlebovirus turunaense* — Turuna virus (TUAV)
  - *Phlebovirus uriuranaense* — Uriurana virus (URIV)
  - *Phlebovirus urucuriense* — Urucuri virus (URUV)
  - *Phlebovirus zerdaliense* — Zerdali virus (ZERV)
- *Pidchovirus*
  - *Pidchovirus pidgei* — Pidgey virus (PGYV)
  - *Pidchovirus stethori* — coleopteran phenui-related virus 308 (CoPhV308)
- *Rubodvirus*
  - *Rubodvirus argentinaense* — grapevine Muscat rose virus (GMRV)
  - *Rubodvirus armeniaense* — grapevine Garan dmak virus (GGDV)
  - *Rubodvirus mali* — apple rubbery wood virus 1 (ARWV1)
  - *Rubodvirus prosserense* — apple rubbery wood virus 2 (ARWV2)
- *Tanzavirus*
  - *Tanzavirus daressalaamense* — Dar es Salaam virus (DeSV)
- *Tenuivirus*
  - *Tenuivirus echinochloae* — Echinochloa hoja blanca virus (EHBV)
  - *Tenuivirus eurotritici* — European wheat striate mosaic virus (EWSMV)
  - *Tenuivirus oryzabrevis* — rice grassy stunt virus (RGSV)
  - *Tenuivirus oryzaclavatae* — rice stripe virus (RSV = RStV)
  - *Tenuivirus oryzalbae* — rice hoja blanca virus (RHBV)
  - *Tenuivirus persotritici* — Iranian wheat stripe virus (IWSV)
  - *Tenuivirus urochloae* — Urochloa hoja blanca virus (UHBV)
  - *Tenuivirus zeae* — maize stripe virus (MSV = MStV = MSpV)
- *Uukuvirus*
  - *Uukuvirus dabieshanense* — Dàbiéshān tick virus (DbsTV = DBTV = DTV)
  - *Uukuvirus dermacentoris* — American dog tick virus (ADV)
  - *Uukuvirus grandarbaudense* — Grand Arbaud virus (GAV)
  - *Uukuvirus hoplandense* — Pacific coast tick virus (PACV)
  - *Uukuvirus huangpiense* — Huángpí tick virus 2 (HpTV2)
  - *Uukuvirus kabutoense* — Kabuto mountain virus (KAMV)
  - *Uukuvirus kaisodiense* — Kaisodi virus (KASDV)
  - *Uukuvirus lihanense* — Lǐhàn tick virus (LITV)
  - *Uukuvirus mocquariense* — Precarious Point virus (PPV)
  - *Uukuvirus rukutamaense* — Rukutama virus (RUKV)
  - *Uukuvirus schmidti* — Nile warbler virus (NWV)
  - *Uukuvirus silverwaterense* — Silverwater virus (SILV)
  - *Uukuvirus tachengense* — Tǎchéng tick virus 2 (TcTV2)
  - *Uukuvirus toyoense* — Tōyo virus (TOYV)
  - *Uukuvirus tyulenyense* — Zaliv Terpeniya virus (ZTV)
  - *Uukuvirus uriae* — murre virus (MURV)
  - *Uukuvirus uukuniemiense* — Chizé virus (CHZV); Fin V 707 virus (FINV); Oceanside virus (OCV = OCEV); Ponteves virus (PTVV); St. Abbs Head virus (SAHV); Uukuniemi virus (UUKV)
  - *Uukuvirus yongjiaense* — Yǒngjiā tick virus (YONV)
- *Wenrivirus*
  - *Wenrivirus penaei* — Mourilyan virus (MoV)

Tospoviridae
- *Orthotospovirus*
  - *Orthotospovirus alstroemerflavi* — Alstroemeria yellow spot virus (AYSV)
  - *Orthotospovirus alstroemerinecrosis* — Alstroemeria necrotic streak virus (ANSV)
  - *Orthotospovirus arachianuli* — groundnut ringspot virus (GRSV)
  - *Orthotospovirus arachiflavamaculae* — groundnut yellow spot virus (GYSV)
  - *Orthotospovirus arachiflavi* — groundnut chlorotic fan-spot virus (GCFSV)
  - *Orthotospovirus arachinecrosis* — groundnut bud necrosis virus (GBNV)
  - *Orthotospovirus calloflavi* — calla lily chlorotic spot virus (CCSV)
  - *Orthotospovirus capsiciflavi* — Capsicum chlorosis virus (CaCV)
  - *Orthotospovirus capsicimaculaflavi* — pepper chlorotic spot virus (PCSV)
  - *Orthotospovirus chrysanthinecrocaulis* — Chrysanthemum stem necrosis virus (CSNV)
  - *Orthotospovirus citrullomaculosi* — watermelon silver mottle virus (WSMoV)
  - *Orthotospovirus citrullonecrosis* — watermelon bud necrosis virus (WBNV)
  - *Orthotospovirus cucurbichlorosis* — zucchini lethal chlorosis virus (ZLCV)
  - *Orthotospovirus glycininecrovenae* — soybean vein necrosis virus (SVNV)
  - *Orthotospovirus hippeflavi* — Hippeastrum chlorotic spot virus (HCRV)
  - *Orthotospovirus impatiensnecromaculae* — impatiens necrotic spot virus (INSV)
  - *Orthotospovirus iridimaculaflavi* — iris yellow spot virus (IYSV)
  - *Orthotospovirus meloflavi* — melon yellow spot virus (MYSV)
  - *Orthotospovirus melotessellati* — melon severe mosaic virus (MSMV)
  - *Orthotospovirus morivenae* — mulberry vein banding-associated virus (MVBaV)
  - *Orthotospovirus phaseolinecrotessellati* — bean necrotic mosaic virus (BeNMV)
  - *Orthotospovirus polygonianuli* — Polygonum ringspot virus (PolRSV)
  - *Orthotospovirus tomatoanuli* — tomato yellow ring virus (TYRV)
  - *Orthotospovirus tomatoflavi* — tomato chlorotic spot virus (TCSV)
  - *Orthotospovirus tomatomaculae* — tomato spotted wilt virus (TSWV)
  - *Orthotospovirus tomatozonae* — tomato zonate spot virus (TZSV)

Tulasviridae
- *Orthotulasvirus*
  - *Orthotulasvirus tulasnellae* — Tulasnella bunyaviralés-like virus 1 (TB-LV)

Wupedeviridae
- *Wumivirus*
  - *Wumivirus millepedae* — Wǔhàn millipede virus 2 (WhMV2)

Insthoviricetes

Articulavirales

Amnoonviridae
- *Tilapinevirus*
  - *Tilapinevirus tilapiae* — tilapia lake virus (TiLV)

Orthomyxoviridae
- *Alphainfluenzavirus*
  - *Alphainfluenzavirus influenzae* — influenza A virus (FLUAV)
- *Betainfluenzavirus*
  - *Betainfluenzavirus influenzae* — influenza B virus (FLUBV)
- *Deltainfluenzavirus*
  - *Deltainfluenzavirus influenzae* — influenza D virus (FLUDV)
- *Gammainfluenzavirus*
  - *Gammainfluenzavirus influenzae* — influenza C virus (FLUCV)
- *Isavirus*
  - *Isavirus salaris* — infectious salmon anemia virus (ISAV)
- *Mykissvirus*
  - *Mykissvirus tructae* — rainbow trout orthomyxovirus (RbtOV); steelhead trout orthomyxovirus (StOV1)
- *Quaranjavirus*
  - *Quaranjavirus araguariense* — Araguari virus (ARAV)
  - *Quaranjavirus chadense* — Lake Chad virus (LKCV)
  - *Quaranjavirus johnstonense* — Johnston Atoll virus (JAV)
  - *Quaranjavirus quaranfilense* — Quaranfil virus (QRFV)
  - *Quaranjavirus tyulekense* — Tyulek (Tiulek) virus (TLKV)
  - *Quaranjavirus wellfleetense* — Wellfleet Bay virus (WFBV)
- *Sardinovirus*
  - *Sardinovirus pilchardi* — pilchard orthomyxovirus (POMV)
- *Thogotovirus*
  - *Thogotovirus bourbonense* — Bourbon virus (BRBV)
  - *Thogotovirus dhoriense* — Dhori virus (DHOV)
  - *Thogotovirus josense* — Jos virus (JOSV)
  - *Thogotovirus ozense* — Oz virus (OZV)
  - *Thogotovirus sinuense* — Sinu virus (SINUV)
  - *Thogotovirus thailandense* — Thailand tick thogotovirus (TT-THOV)
  - *Thogotovirus thogotoense* — Thogoto virus (THOV)
  - *Thogotovirus upoluense* — Upolu virus (UPOV)

Unassigned

Tosoviridae
- *Fraservirus*
  - *Fraservirus testudinis* — turtle fraservirus 1 (TFV1)

Legend: *Phylum* | *Subphylum* | *Class* | *Order* | *Family* | *Subfamily* | *Genus* | *Species* | Virus (abbreviation)
